# Based on untargeted metabolomics and metagenomics: a study on the mechanism of Miao ethnomedicine *Zingiber mioga* (Thunb.) Rosc. in treating slow transit constipation

**DOI:** 10.3389/fmicb.2026.1751739

**Published:** 2026-02-27

**Authors:** Yutao Du, Le Chen, Xia Zhang, Jiali Zeng, Chenggang Hu

**Affiliations:** Department of Pharmacy, School of Pharmaceutical Sciences, Guizhou University of Traditional Chinese Medicine, Guiyang, Guizhou, China

**Keywords:** gut microbiota, metabolomics, metagenomics, slow transit constipation, *Zingiber mioga* (Thunb.) Rosc.

## Abstract

**Introduction:**

Slow transit constipation (STC) is a prevalent gastrointestinal disorder characterized by impaired intestinal motility, metabolic dysregulation, and gut microbial dysbiosis. *Zingiber mioga* (Thunb.) Rosc. (RH), a traditional medicinal-edible plant, is empirically used to alleviate gastrointestinal dysfunction, but its therapeutic mechanisms in STC remain unclear. Herein, we investigated the laxative efficacy and mechanism of RH in a rat STC model via integrated untargeted metabolomic and metagenomic analyses, providing experimental evidence for its clinical use.

**Methods:**

A rat STC model was established by intragastric loperamide hydrochloride (5 mg/kg) for 35 consecutive days. Thirty-six SD rats were randomly divided into six groups (*n* = 6): normal control, STC model, mosapride-positive control (2 mg/kg), and low- (1350 mg/kg), medium- (2700 mg/kg), high-dose (3400 mg/kg) RH groups, with concurrent drug intervention. Serum concentrations of SP, MTL, and GAS (key gastrointestinal motility regulators) were quantified. Colonic pathological damage was histopathologically evaluated, and intestinal propulsive rate was measured. Untargeted serum metabolomics and fecalmetagenomics identified differential metabolites and gut microbiota alterations.

**Results:**

Compared with the STC model, RH significantly reduced serum SP (intestinal motility inhibitor) and increased MTL/GAS (motility promoters). It also dose-dependently ameliorated colonic lesions and improved intestinal propulsive rate. Serum metabolomics identified 15 differential metabolites, mainly enriched in nitrogen metabolism, neuroactive ligand–receptor interaction, and amino acid metabolism. Fecal metagenomics showed RH restored the Eubacteriales/Lachnospirales ratio (a STC dysbiosis marker) and increased beneficial genera (e.g., *Ruminococcus* sp., *Eubacterium* sp.).

**Discussion and conclusion:**

Our findings show RH effectively ameliorates colonic injury and gastrointestinal motility in STC rats, associated with regulating gastrointestinal hormone secretion. Its benefits are likely mediated by improving dysregulated amino acid/nitrogen metabolism and modulating gut microbiota composition. This study provides mechanistic evidence for RH as a natural functional agent for STC management, laying a foundation for exploring its active components and clinical translation.

## 1 Introduction

Functional constipation is a common and prevalent clinical condition, classified into normal transit, slow transit, defecatory disorder, and mixed subtypes based on pathophysiological characteristics. Among these, STC is a non-organic subtype primarily characterized by gastrointestinal transit dysfunction, with intestinal hypoperistalsis as a salient feature (Fu et al., 2022). Clinical manifestations of STC include decreased defecation frequency, difficult defecation, and dry, hard stools; severe cases may even trigger cardiovascular and cerebrovascular complications. To date, the pathogenesis of STC remains incompletely elucidated, but it is widely acknowledged to be closely associated with impaired intestinal motility, enteric neuronal loss, intestinal inflammation, enteric nerve dysfunction, and increased apoptosis of interstitial cells of Cajal (Drossman and Hasler, 2016; Ren et al., 2022; Wen-yan et al., 2022; Wu et al., 2020; Zeyue et al., 2022; Zheng et al., 2021). Mounting evidence suggests that strains of the genera *Bifidobacterium* and *Lactobacillus* can modulate intestinal 5-hydroxytryptamine (5-HT) levels via the production of γ-aminobutyric acid (GABA), thereby enhancing intestinal motility in patients with STC (Chen et al., 2022; Schwörer et al., 1989; Strandwitz, 2018; Wang et al., 2022).

RH, also known as “tuli kaihua,” “yanghuo,” “yanghuojiang,” or “yanhuo,” is widely used in traditional Chinese medicine (TCM) practice. It exhibits pharmacological activities including resolving phlegm and relieving cough, promoting blood circulation and regulating menstruation, and detoxifying and reducing swelling, and is mainly indicated for the treatment of cough, asthma, irregular menstruation, and abdominal distension and pain (Editorial Committee Of Chinese Materia Medica, 1999). As a perennial medicinal herb belonging to the Zingiberaceae family, RH is distributed in Zhejiang, Jiangsu, Guangdong, Guizhou, and other regions of China. In ethnic minority areas of Guizhou Province, it is commonly employed as a folk remedy and is called “jab jenl mail” by the Miao people (Junhua et al., 2002; Kemin and Furong, 2006; Shunzhi and Wenfen, 2007). Besides its medicinal value, the tender leaves and inflorescences of RH are edible as vegetables; the inflorescences are frequently incorporated into antitussive formulations, and its roots contain nutrients (e.g., proteins, fats, dietary fiber, trace elements, and essential amino acids) as well as bioactive constituents such as flavonoids and polysaccharides (Jinping et al., 2013). Numerous studies have shown that RH extracts and metabolites possess diverse biological activities, such as antimicrobial, antitumor, hypoglycemic, antioxidant, anti-obesity, skin-moisturizing, whitening, and anti-wrinkle properties (Abe et al., 2006; Abe et al., 2004; Jo et al., 2016). Notably, studies by Fangli et al. (2022) on the aqueous extract, alcohol-soluble fraction, and alcohol-precipitated fraction of RH have revealed that the aqueous extract of RH exerts a potent laxative effect.

Emerging evidence has indicated that the gut microbiota and their metabolites undergo substantial alterations in constipated patients, and modulation of gut microbiota composition may serve as a key therapeutic strategy for STC (Xu et al., 2022). Metabolomics allows for high-throughput identification and analysis of metabolic changes, thereby facilitating the screening of differential metabolites during disease progression or treatment (Cui et al., 2018). In recent years, metabolomics has been successfully employed to explore biomarkers and mechanisms underlying the TCM-based treatment of STC (Kang, 2024; Pengjun and Bin, 2023; Zeyang, 2023). The gut microbiota, consisting of diverse microorganisms inhabiting the intestinal tract, plays an indispensable role in regulating host metabolism (Agus et al., 2021). Investigating disease pathogenesis from the perspective of the gut microbiota has emerged as a novel research focus, especially for metabolic-related disorders like STC (Han et al., 2024). Compared with traditional 16S rRNA gene sequencing, metagenomics offers more comprehensive insights into microbial communities, allowing for further exploration of functional genes, metabolic pathways, and functional potential (Kim et al., 2024).

Therefore, the integration of metabolomics and metagenomics is anticipated to yield novel insights into the pathogenesis of STC and the therapeutic mechanisms of TCM. In the present study, an STC rat model was established to evaluate the therapeutic potential of the aqueous extract of RH, a Miao ethnomedicine. By integrating metabolomic and metagenomic approaches, we aimed to comprehensively elucidate the underlying mechanisms by which RH regulates metabolism and the intestinal microbiota in STC, thereby providing a scientific basis for a deeper understanding of its therapeutic efficacy and opening new avenues for the management of STC.

## 2 Materials and methods

### 2.1 Materials

**Figure 1:**
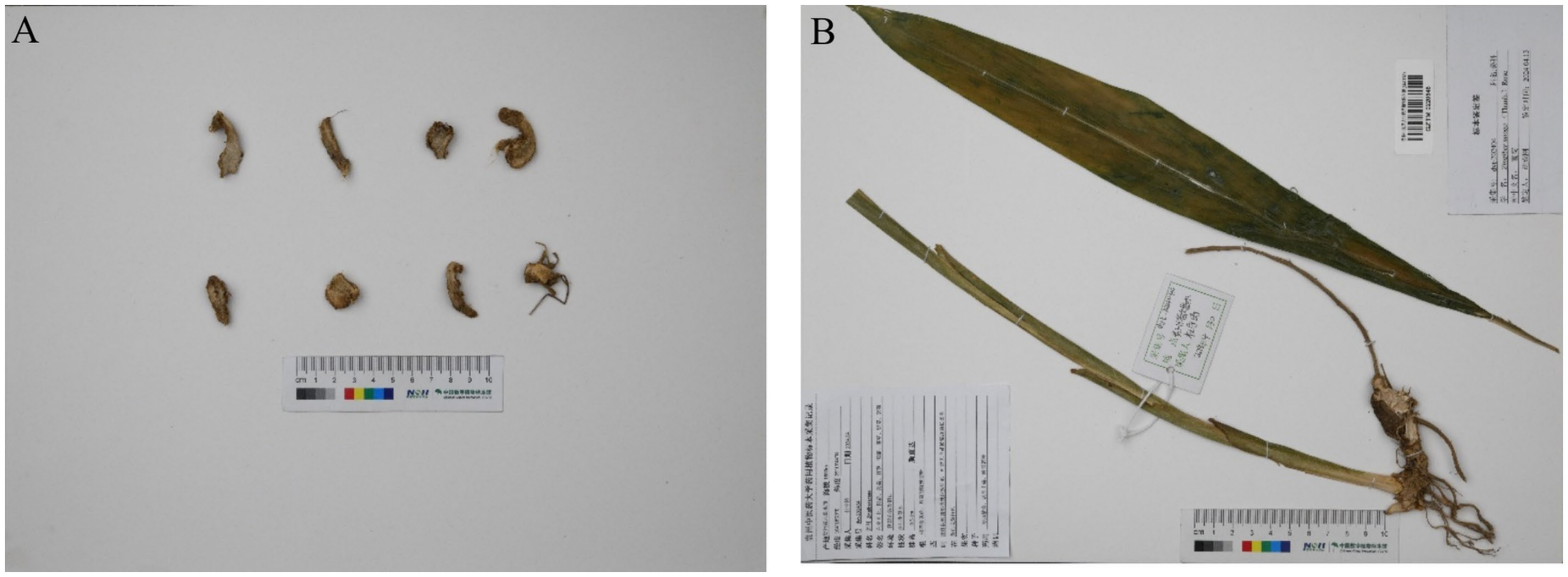
Authentic voucher specimens of RH. **(A)** Dried rhizomes of RH; **(B)** whole plant voucher specimen.

Rhizomes of RH, a natural medicinal herb, were purchased from Liupanshui, Guiyang, China. A voucher specimen (voucher number: dyt-20240410) was identified by Professor Chenggang Hu from Guizhou University of TCM (Figure 1).

Aqueous extract of RH was prepared via decoction: 6000 mL of ultrapure water was heated to boiling, and 600 g of RH rhizomes (solid-to-liquid ratio 1:10, w/v) was added for decoction. After boiling for 3 min, the mixture was filtered through 8 layers of 200-mesh nylon fabric. The residue was re-decocted with 600 mL of boiling water three times. Filtrates from all decoctions were combined, and the residue was discarded. The combined filtrate was concentrated under reduced pressure at 50 °C to a relative density of 2.000, followed by freeze-drying for 3 consecutive days. The resulting extract was stored long-term at −80 °C for animal experiments; prior to administration, it was diluted to the required concentration and stored short-term at 4 °C.

Loperamide hydrochloride was purchased from Sigma-Aldrich (St. Louis, MO, USA; batch number: L4762-5G). Ultra-high performance liquid chromatography-tandem Fourier transform mass spectrometry (UHPLC-Orbitrap Exploris 240) was acquired from Thermo Fisher Scientific (Waltham, MA, USA). An HSS T3 chromatographic column (100 mm × 2.1 mm i.d., 1.8 μm) was purchased from Waters Corporation (Milford, MA, USA).

Instrumentation included: a JXDC-20 nitrogen evaporator (Shanghai Jingxin Industrial Development Co., Ltd., Shanghai, China); an LNG-T88 desktop rapid centrifugal concentrator (Taicang Huamei Biochemical Instrument Factory, Taicang, China); a Wonbio-96c high-throughput tissue homogenizer (Shanghai Wanbai Biotechnology Co., Ltd., Shanghai, China); an SBL-10DT ultrasonic cleaner (300 W, 10 L; Ningbo Xinzhi Biotechnology Co., Ltd., Ningbo, China); a Centrifuge 5430R high-speed refrigerated centrifuge (Eppendorf AG, Hamburg, Germany); and a NewClassic MF MS105DU electronic balance (Mettler Toledo, Greifensee, Switzerland), used as per the manufacturer’s specifications.

UPLC-grade methanol, propanol, water, acetonitrile, and formic acid were purchased from Fisher Scientific (Waltham, MA, USA). ELISA kits for rat Substance P (SP), Motilin (MTL), and Gastrin (GAS) (all batch number: 202505) were purchased from Changzhou Chenjun Chemical Co., Ltd. (Changzhou, China).

### 2.2 Animals and groups

Thirty-six specific pathogen-free (SPF) male Sprague–Dawley (SD) rats, weighing 180–220 g, were purchased from Spence (Beijing) Biotechnology Co., Ltd. (license number: SCXK (Jing) 2024-0001). This experiment was approved by the Experimental Animal Management and Ethics Committee of Guizhou University of TCM (approval number: 20251111002). Rats were housed individually in a controlled environment with a temperature of 22 ± 1 °C, relative humidity of 63 ± 5%, and a 12 h light/dark cycle, with ad libitum access to food and water. After 2 weeks of acclimatization, STC was induced in all groups except the control group by intragastric administration of loperamide hydrochloride (5 mg/kg) twice daily for 1 week, followed by once daily for an additional 4 weeks. The rats were randomly divided into 6 groups (*n* = 6 per group): Normal group (Control): No loperamide hydrochloride; Model group (Model): Loperamide hydrochloride (5 mg/kg); Positive control group (Positive): Mosapride citrate capsules (2 mg/kg) + loperamide hydrochloride (5 mg/kg); Low-dose RH group (Low): RH (aqueous extract of RH) (1,350 mg/kg) + loperamide hydrochloride (5 mg/kg); Medium-dose RH group (Middle): RH (2,700 mg/kg) + loperamide hydrochloride (5 mg/kg); High-dose RH group (High): RH (5,400 mg/kg) + loperamide hydrochloride (5 mg/kg).

### 2.3 Preparation of drugs

To quantitatively evaluate the improvement of intestinal motility by RH in STC rats, an activated carbon suspension was prepared as an intestinal propulsion tracer. Briefly, 2 g of sodium carboxymethylcellulose was dissolved in 100 mL of sterile water and heated to boiling until the solution became transparent. Subsequently, 5 g of activated carbon was added to the mixture, and the suspension was reboiled three times to ensure uniform dispersion. After cooling to room temperature, the volume was adjusted to a final volume of 100 mL with sterile water, yielding a 50 g/L activated carbon suspension. The prepared suspension was stored at 4 °C until use.

### 2.4 Identification of components in aqueous extract of RH

To systematically characterize the chemical composition of the RH aqueous extract and identify potential bioactive components (to support subsequent investigations into its material basis and mechanism of regulating intestinal microecology in STC), ultra-high performance liquid chromatography-Fourier transform mass spectrometry (UHPLC-QExactive) was employed for qualitative and quantitative analysis.

Briefly, 100 mg of the extract prepared in Section 2.1 was placed in a 2 mL centrifuge tube containing one 6 mm-diameter grinding bead. Subsequently, 300 μL of extraction solution (methanol:water = 4:1, v/v) supplemented with four internal standards (including L-2-chlorophenylalanine at 0.02 mg/mL) was added to extract metabolites. Analyses were performed on a UHPLC-QExactive system (Thermo Fisher Scientific, Waltham, MA, USA) equipped with an HSS T3 chromatographic column (100 mm × 2.1 mm i.d., 1.8 μm; Waters Corporation, Milford, MA, USA). The column temperature was maintained at 40.0 °C, with a flow rate of 0.400 mL min^−1^ using a gradient elution mobile phase consisting of solvent A (water-acetonitrile = 95:5, v/v, containing 0.1% formic acid) and solvent B (acetonitrile-isopropanol-water = 47.5:47.5:5, v/v/v, containing 0.1% formic acid). To ensure system stability and analytical reproducibility, quality control (QC) samples were prepared by pooling equal volumes of all test samples, with one QC sample analyzed every 5–10 test samples to verify the reliability of the analytical workflow.

Mass spectrometry conditions: Samples were ionized via electrospray ionization (ESI), and mass spectral signals were acquired in both positive and negative ion modes. Specific parameters were set as follows: scan range = 70–1,050 m/z; sheath gas flow rate = 50 arb; auxiliary gas flow rate = 13 arb; auxiliary gas heating temperature = 450 °C; capillary temperature = 320 °C; spray voltage = 3,500 V (positive mode) and −3,000 V (negative mode); S-Lens voltage = 40; collision energies = 20, 40, and 60%; full scan (Full MS) resolution = 70,000; and MS^2^ resolution = 17,500. Raw data obtained from both ionization modes were combined for subsequent data processing and multivariate statistical analysis.

### 2.5 Determination of intestinal propulsion rate

Following the completion of modeling and the final administration, rats were fasted for 24 h with ad libitum access to water. Each rat was administered an intragastric dose of 5% activated carbon suspension at 1 mL/100 g body weight. Thirty minutes post-administration, rats were anesthetized via intraperitoneal injection of 5% urethane at 0.6 mL/100 g body weight. Immediately following anesthesia, the abdominal cavity was opened to collect blood from the abdominal aorta, and the entire intestinal tract was rapidly excised. Under tension-free conditions, the total length of the intestinal tract and the propulsive length of the activated carbon were measured. The intestinal propulsion rate was calculated using the following formula:


$$\text{Intestinal propulsion rate}=(\begin{array}{l} \text{Propulsive length of}\;\\ \text{activated carbon}/\\ \text{Total length of the}\;\\ \text{intestinal tract} \end{array})\times 100\%$$

### 2.6 HE staining for observing colonic pathological changes

Following the final administration, colonic tissues were collected from 3 randomly selected rats per group. The tissues were fixed in 4% paraformaldehyde and subsequently subjected to precision dissection, dehydration, embedding, sectioning, hematoxylin–eosin (HE) staining, and mounting in accordance with standard pathological protocols. The sections were observed under a light microscope at different magnifications, and detailed records were made of basic pathological changes and inter-sectional variations.

### 2.7 ELISA determination

Following the completion of all administrations, 5 mL of abdominal blood was collected from each rat. Blood samples were incubated at 37 °C for 30 min to allow coagulation, then centrifuged at 3500 rpm for 10 min. The supernatant (serum) was aspirated and stored at −80 °C for later use.

One aliquot of the serum was used to quantify serum levels of substance P (SP), gastrin (GAS), and motilin (MTL) in 3 rats per group using commercial ELISA kits following the manufacturer’s protocols. The remaining supernatant was reserved for untargeted metabolomics analysis.

### 2.8 Fecal metagenomic detection

Fecal samples were transported to Shanghai Majorbio Bio-pharm Technology Co., Ltd. (Shanghai, China) on dry ice for metagenomic analysis. Total genomic DNA of the microbial community was extracted using the E.Z.N.A.® Soil DNA Kit (Omega Bio-tek, Norcross, GA, USA) following the manufacturer’s protocols. DNA concentration and purity were quantified, and DNA integrity was evaluated via 1% agarose gel electrophoresis.

Genomic DNA was fragmented using a Covaris M220 system (Gene Company, China), and approximately 350 bp fragments were selected for paired-end (PE) library construction with the NEXTFLEX Rapid DNA-Seq kit (Bioo Scientific, USA). Metagenomic sequencing was performed on the Illumina NovaSeq™ X Plus platform (Illumina, San Diego, CA, USA) by Shanghai Majorbio Bio-pharm Technology Co., Ltd.

Bioinformatic analyses, including microbial species annotation, community composition analysis, diversity index calculation, and differential species identification, were performed based on operational taxonomic units (OTUs) to acquire data regarding microbial composition, abundance, and differential alterations. All analytical processes were conducted on the Majorbio Bio-Cloud Platform (https://www.majorbio.com/web/market/activity; Shanghai Majorbio Bio-pharm Technology Co., Ltd.).

### 2.9 Serum untargeted metabolomics analysis

Three rats per group were randomly selected for blood sampling. Blood was collected in heparinized anticoagulant tubes, centrifuged at 3500 rpm for 10 min, and the supernatant (plasma) was collected, stored at −80 °C, and subsequently transported to Shanghai Majorbio Bio-pharm Technology Co., Ltd. (Shanghai, China) on dry ice for metabolomic analysis.

#### 2.9.1 Sample preparation

Precisely transfer 100 μL of plasma into a 1.5 mL centrifuge tube; add 300 μL of extraction solution (methanol:acetonitrile = 1:1, v/v) containing four internal standards, vortex for 30 s, and undergo low-temperature ultrasonic extraction at 5 °C (40 kHz) for 30 min. Samples were incubated at −20 °C for 30 min to precipitate proteins, then centrifuged at 13,000 × *g* for 15 min at 4 °C. The supernatant was transferred and dried under a nitrogen stream, then reconstituted with 100 μL of reconstitution solution (acetonitrile:water = 1:1, v/v), vortexed for 30 s, and subjected to low-temperature ultrasonic extraction at 5 °C (40 kHz) for 5 min. After centrifugation at 13,000 × *g* for 10 min at 4 °C, the supernatant was transferred to an injection vial equipped with an inner liner for instrumental analysis. Additionally, 20 μL of supernatant from each sample was pooled to prepare quality control (QC) samples, which were used to monitor analytical stability.

#### 2.9.2 Chromatographic conditions

An ACQUITY UPLC HSS T3 column (100 mm × 2.1 mm i.d., 1.8 μm; Waters Corporation, Milford, MA, USA) was employed for chromatographic separation. Mobile phase A was composed of 95% water + 5% acetonitrile (containing 0.1% formic acid), and mobile phase B was composed of 47.5% acetonitrile + 47.5% isopropanol + 5% water (containing 0.1% formic acid). The injection volume was 3 μL, and the column temperature was maintained at 40 °C.

#### 2.9.3 Mass spectrometric conditions

Samples were ionized via electrospray ionization (ESI), with mass spectral signals acquired in both positive and negative ion modes. Key parameters were set as follows: scan range = 70–1,050 m/z; sheath gas flow rate = 50 psi; auxiliary gas flow rate = 13 psi; auxiliary gas heating temperature = 425 °C; capillary temperature = 325 °C; ion spray voltage = 3,500 V (positive mode) and −3,500 V (negative mode); cyclic collision energies = 20–40–60 eV; full scan (Full MS) resolution = 60,000; and MS^2^ resolution = 7,500.

#### 2.9.4 Data processing and metabolite identification

Raw LC–MS data were imported into Progenesis QI software (Waters Corporation, Milford, MA, USA) for baseline filtering, peak detection, integration, retention time correction, and peak alignment, generating a data matrix containing retention time, mass-to-charge ratio (m/z), and peak intensity. Metabolite identification was performed by matching MS and MS/MS spectra against the Human Metabolome Database (HMDB, https://hmdb.ca/), Metlin,^1^ and the Majorbio in-house metabolite database.

### 2.10 Correlation analysis between species and metabolites

Spearman correlation analysis was conducted to assess the associations between gut microbiota and differential metabolites. The top 20 microbial species and 15 differential metabolites across all groups were chosen for targeted analysis.

### 2.11 Data analysis

Defecation-related parameters were presented as mean ± standard deviation (SD). Normally distributed data were analyzed using one-way analysis of variance (ANOVA) followed by the Bonferroni *post-hoc* test for multiple group comparisons. The Kruskal–Wallis *H* test was employed to compare microbial abundance at the order and species levels, and functional modules. Statistical significance was set at *p* < 0.05.

## 3 Result

### 3.1 Chemical composition profiling of the aqueous extract of RH via UPLC-Q-Exactive Orbitrap-MS/MS

**Figure 2:**
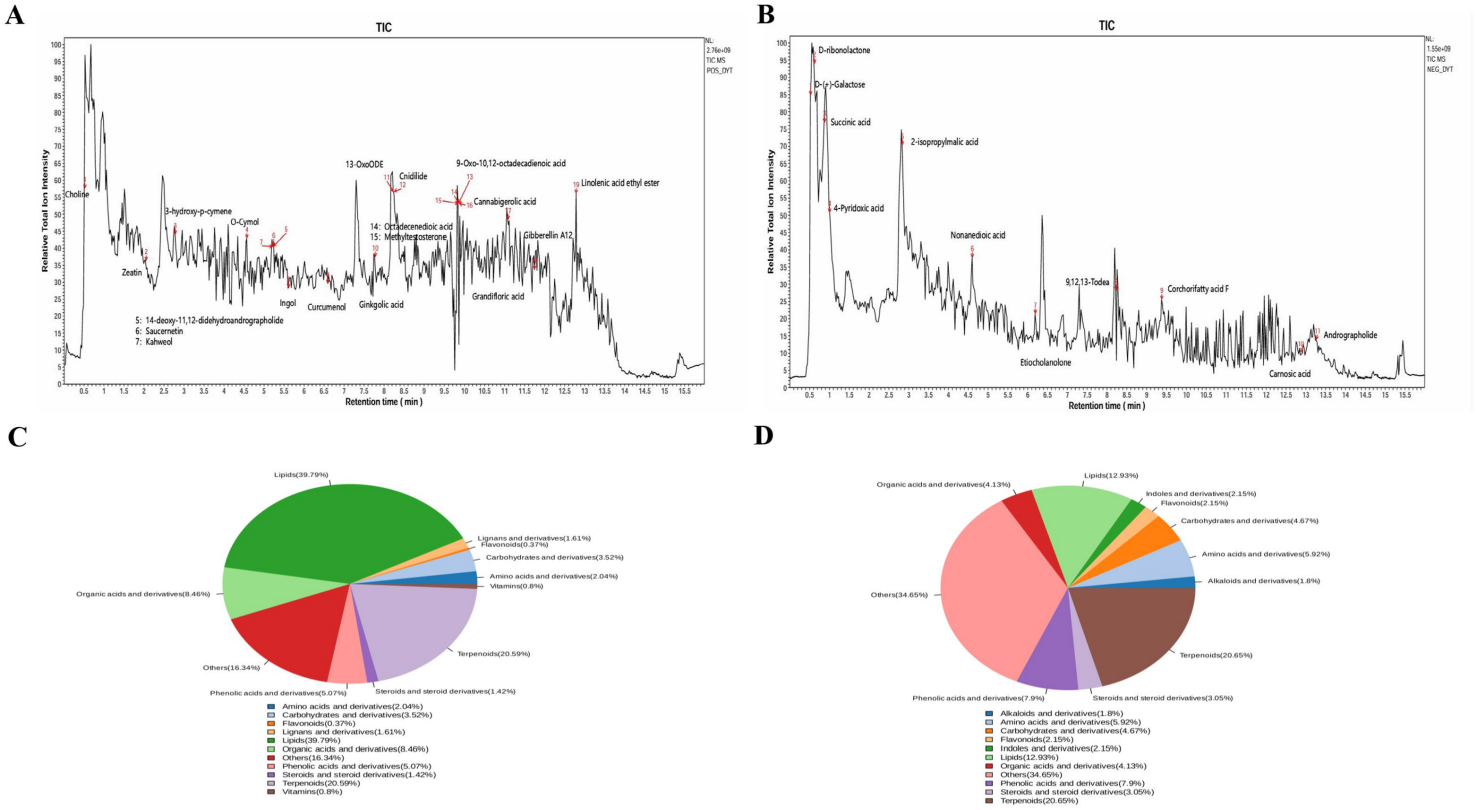
Identification results of the aqueous extract of RH. **(A)** Positive ion mode (POS) TIC, **(B)** negative ion mode (NEG) TIC, **(C)** content distribution by component class of RH ingredients; **(D)** quantity distribution by component class of RH ingredients.

Untargeted metabolic profiling of the RH aqueous extract was conducted via ultra-high performance liquid chromatography-quadrupole-electrostatic field orbitrap tandem mass spectrometry (UPLC-Q-Exactive Orbitrap-MS/MS). The total ion chromatograms (TICs) in positive ion mode (Figure 2A) and negative ion mode (Figure 2B) both displayed robust technical profiles with: in negative ion mode, representative metabolites including D-ribonolactone and succinic acid were successfully detected, whereas positive ion mode yielded higher signal response intensities and identified bioactive substances such as choline and zeatin. Dual-ion mode detection effectively complemented response data for metabolites with varying polarities, furnishing a comprehensive and robust chromatographic-mass spectrometric foundation for the subsequent qualitative identification of 614 distinct compounds.

Based on the component content distribution pie chart (Figure 2C), lipids (39.79%) and terpenoids (20.59%) represented the core dominant constituents of the RH aqueous extract, collectively contributing 51.38% to the total extract content. In contrast, the component quantity distribution pie chart (Figure 2D) demonstrated that the ‘Others’ category exhibited the highest proportion of total compounds (34.65%), with terpenoids ranking second (20.65%); lipids, however, constituted merely 12.99% of the total compound count.

Subsequent cross-analysis of these two distribution profiles indicated that terpenoids were the sole category ranking among the top tiers for both, thus exhibiting both high content abundance and substantial species diversity. Lipids displayed a marked characteristic of —specifically, this category comprised fewer compound species, yet each individual constituent possessed high abundance. The ‘Others’ category, conversely, was characterized by, which indicated the presence of numerous unclassified trace secondary metabolites within this group.

In summary, the RH aqueous extract exhibits a distinct component distribution pattern characterized by. This finding provides a definitive material foundation for subsequent screening of bioactive components, elucidation of the pharmacodynamic material basis, and investigation into the underlying mechanism of action of RH in relevant biological processes.

### 3.2 Detection of intestinal propulsion rate

**Figure 3:**
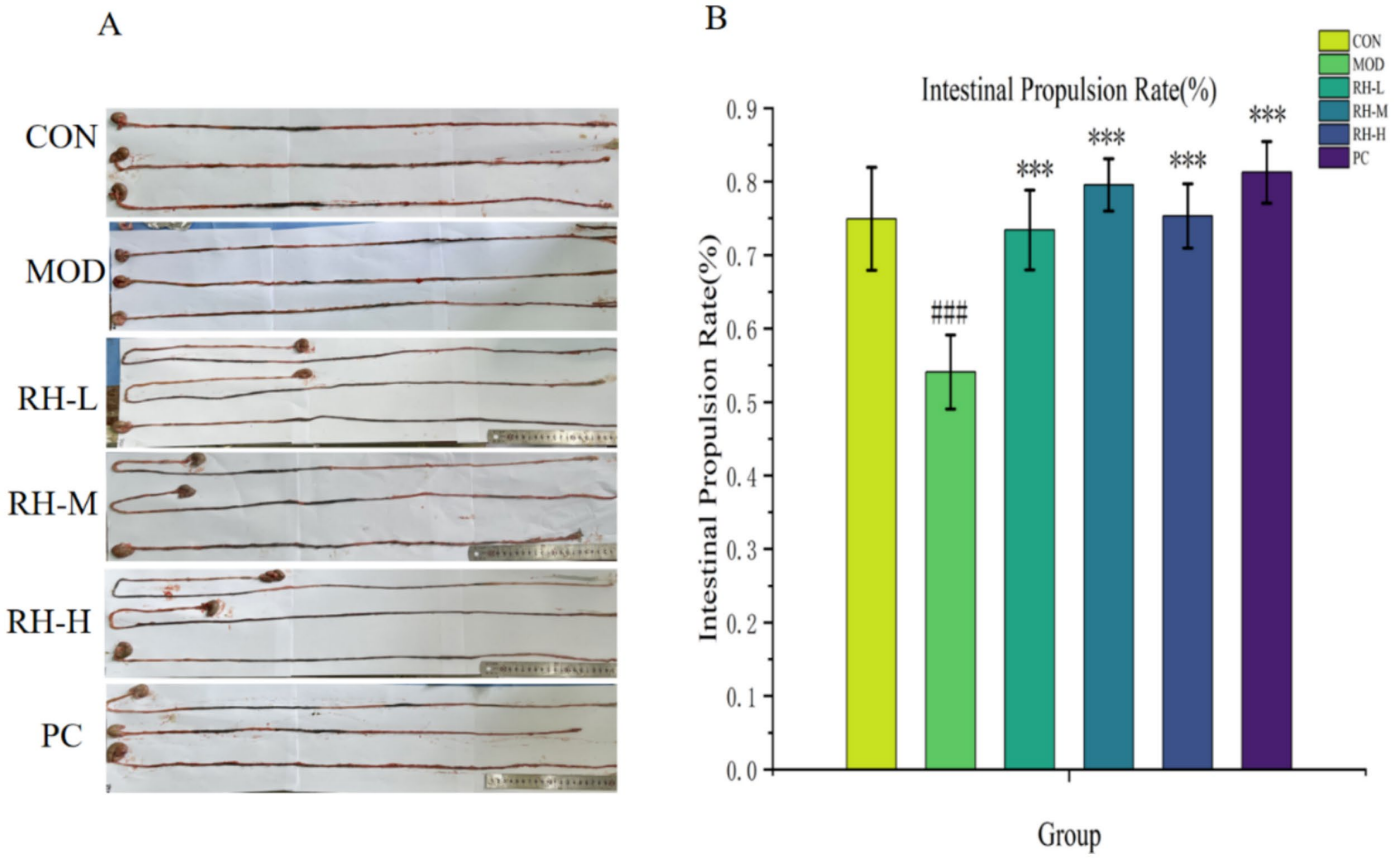
Gastrointestinal transit rate in each group. **(A)** Representative images of intestinal carbon powder propulsion across groups (*n* = 6), **(B)** quantitative analysis of intestinal propulsion rate (*n* = 6). Data are displayed as mean ± SD. ^###^*p* < 0.001 compared to normal group; ^***^p < 0.001 compared to model group.

Intestinal propulsion rates were measured in all groups (Figure 3). A significant reduction in intestinal propulsion rate was observed in the model group compared to the control group (***p < 0.001), confirming the successful establishment of the STC model. In contrast, the RH low-, medium-, and high-dose groups, as well as the positive control group, exhibited significantly higher intestinal propulsion rates compared to the model group (***p < 0.001), demonstrating that RH is effective in enhancing intestinal motility.

### 3.3 Observation of colonic pathological changes by HE staining

In the control group, the colonic mucosal layer of rats was moderately thick and structurally intact, with no atrophy or edema. Epithelial cells were closely and regularly arranged, crypt depth was normal, and the proliferation and differentiation of crypt epithelial cells maintained a dynamic balance. In contrast, the model group exhibited a significant thinning of the colonic mucosal layer (*p* < 0.01) and a reduction in crypt depth (*p* < 0.001), indicating severe pathological damage to the colonic mucosa and confirming successful STC model establishment.

**Figure 4:**
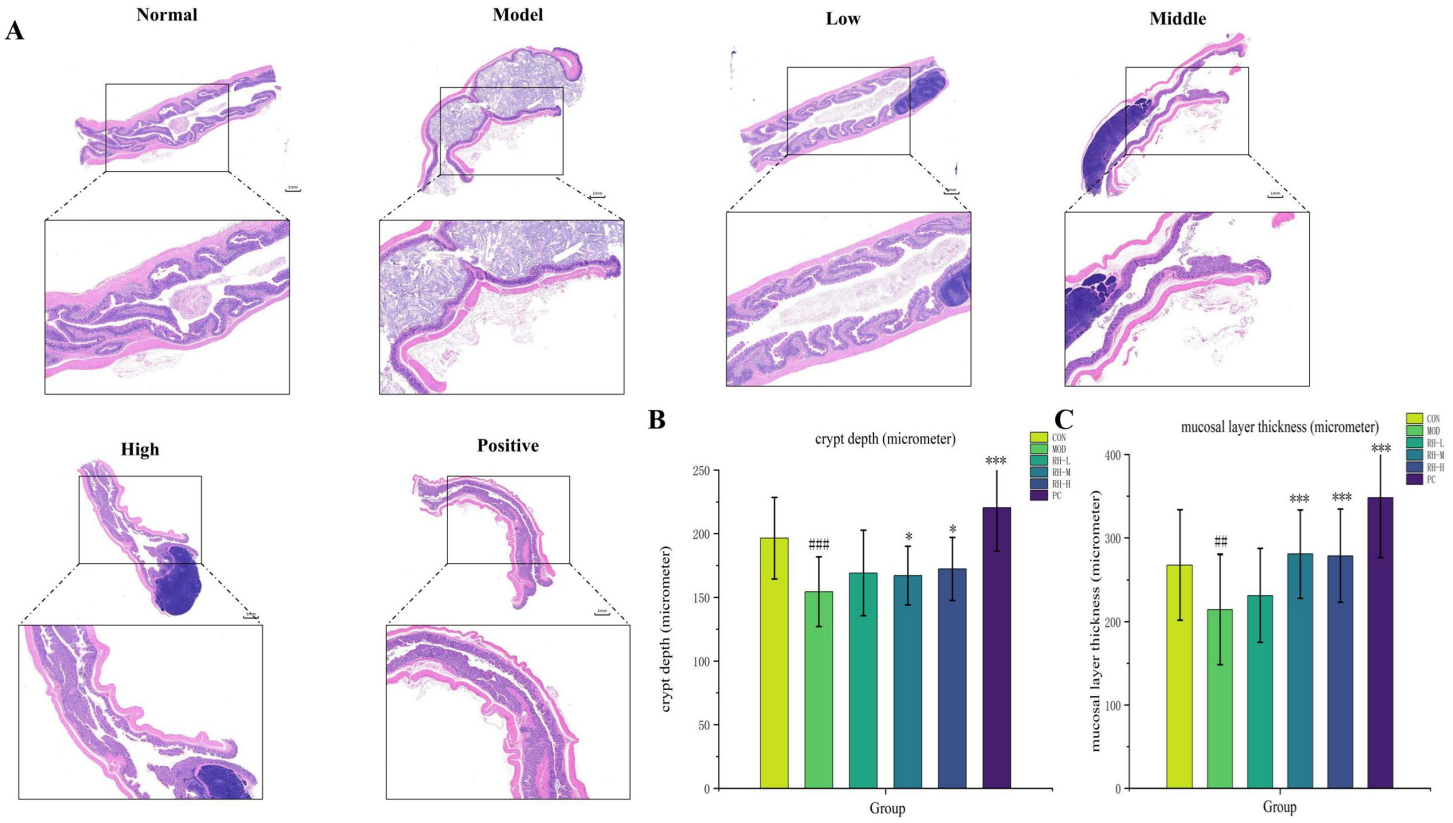
Colonic staining using hematoxylin and eosin. **(A)** H&E staining of rat’s colon tissues (magnification = 5× and 20×; *n* = 3). **(B)** Mucosal layer thickness in the colon of rats of each group (*n* = 3). **(C)** Crypt depth in the colon of rats of each group (*n* = 3). Data are displayed as mean ± SD. Compared to normal group, ^###^*p* < 0.001, ^##^*p* < 0.01, compared to model group, ^*^p < 0.01, ^***^p < 0.001.

Following drug intervention, the RH medium-dose, high-dose, and positive control groups showed significant restoration of mucosal layer thickness (increased) and crypt depth (enhanced), with values approaching those of the control group (Figure 4). Notably, no significant differences in these pathological parameters were detected between the RH low-dose group and the model group, indicating that RH exerts a dose-dependent reparative effect on colonic mucosal injury.

### 3.4 Determination of serum P, GAS, and MTL levels by ELISA

**Figure 5:**
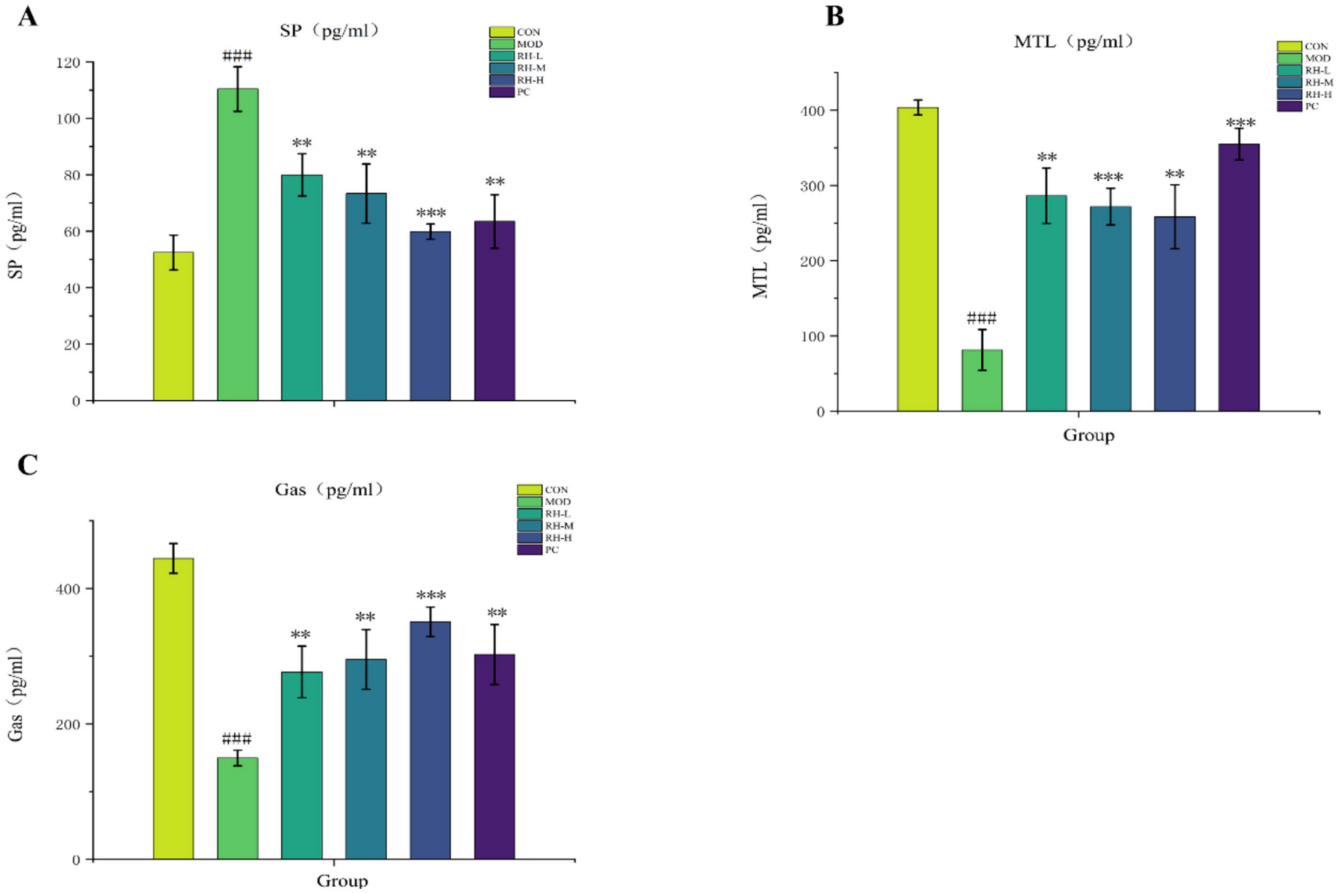
Serum SP, MTL, and GAS levels in each group. **(A)** Serum SP level (*n* = 3, pg/mL), **(B)** serum MTL level (*n* = 3, pg/mL), **(C)** serum GAS level (*n* = 3, pg/mL). Data are displayed as mean ± SD. Compared to normal group, ^###^*p* < 0.001, compared to model group, **p < 0.01, ***p < 0.001.

Following 4 weeks of drug intervention, the positive control group, as well as the RH low-, medium-, and high-dose groups, exhibited a significant increase in serum motilin (MTL) and gastrin (GAS) levels (*p* < 0.05) and a significant decrease in serum substance P (SP) levels (*p* < 0.001) compared to the model group (Figure 5), thereby further validating the laxative effect of RH.

### 3.5 The effect of RH on intestinal flora in STC rats

**Figure 6:**
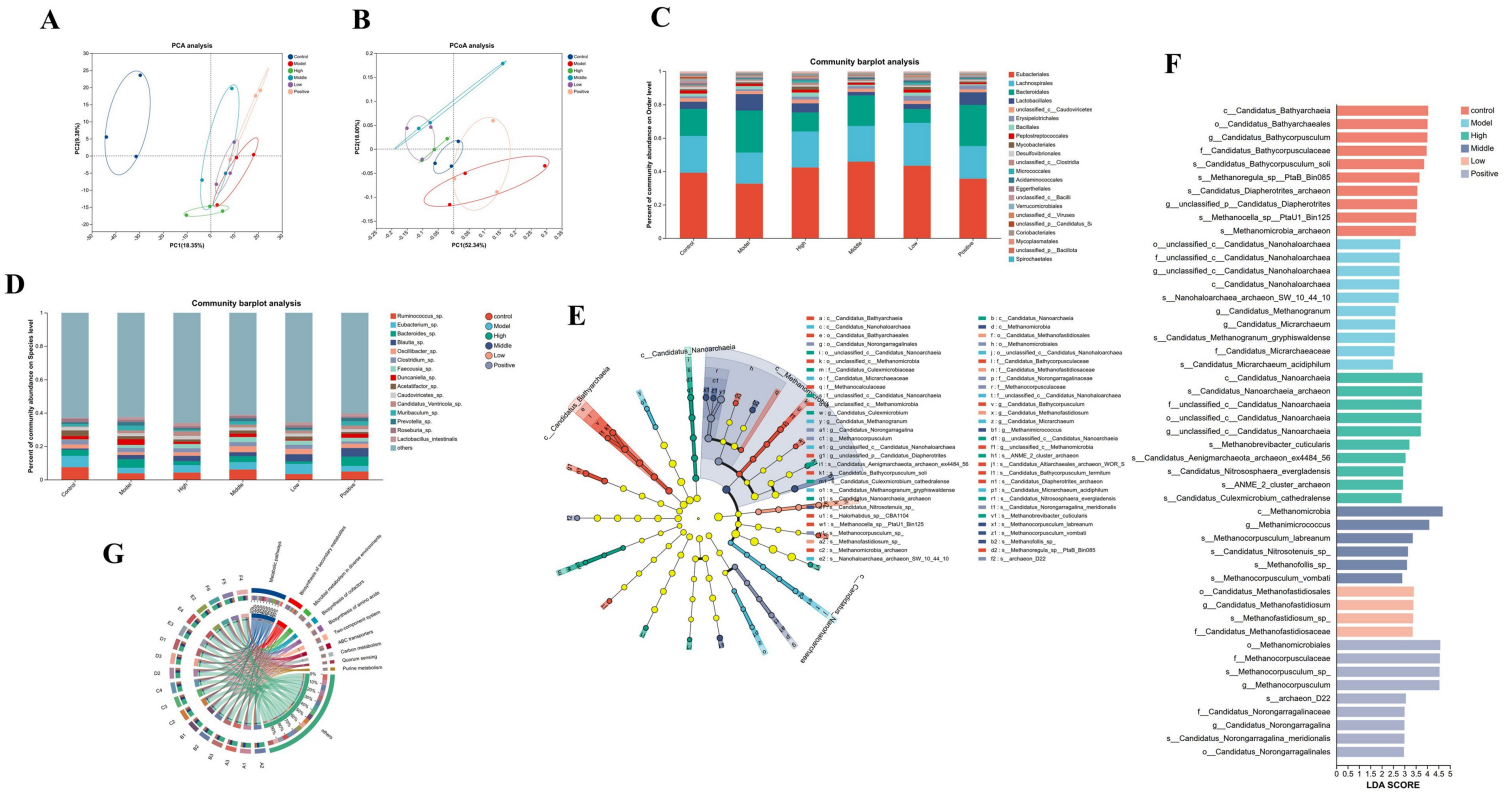
Study on the microbial diversity of RH. **(A)** Beta diversity as determined using PCA plots, **(B)** Beta diversity as determined using PCoA plot, **(C)** Relative abundance at order, **(D)** Relative abundance at species. The *x*-axis represents sample names, and the *y*-axis represents the proportion of each species in the corresponding sample. Bars of different colors correspond to different species, while bar length indicates the magnitude of the species’ proportion. Group labels are displayed above the bars corresponding to each experimental group, **(E)** LEfSe histograms at the species, genus, family, order, class, and phylum levels (LDA score > 4), **(F)** LEfSe LDA value distribution bar chart, displaying differential microbiota effect size, **(G)** Sample-species abundance correlation chord diagram.

Metagenomic analysis, based on high-throughput sequencing of microbial gene sequences, enables precise characterization of gut microbiota composition at the species level. In this study, species annotation of gut microbiota from each group was performed using sequencing-derived gene sequences and relative abundance data. Beta diversity analysis (principal component analysis, PCA; principal coordinate analysis, PCoA) was employed to visualize variations in microbial community structure. Results indicated that microbial clustering in the model group was significantly distinct from that in the control group, demonstrating severe gut microbiota dysbiosis induced by STC modeling. Following RH intervention, microbial clustering in each treatment group gradually approximated that of the control group, confirming that RH effectively reverses gut microbiota imbalance in STC rats (Figures 6A,B).

Further analysis of microbial composition at the order level identified core dominant taxa, including *Eubacteriales*, *Lachnospirales* (spirochete-related groups), *Bacteroidales*, and *Lactobacillales*. Compared to the control group, the model group exhibited significantly decreased relative abundances of *Eubacteriales* and *Lachnospirales*, alongside increased abundances of *Bacteroidales* and *Lactobacillales*. RH intervention increased the relative abundances of *Lachnospirales* and *Bacteroidales*, decreased the abundance of *Lactobacillales*, with the most critical restoration observed for *Eubacteriales* and *Lachnospirales* (Figure 6C). Functionally, *Eubacteriales* and *Lachnospirales* are core producers of intestinal short-chain fatty acids (SCFAs, i.e., butyrate and acetate). Their proportional imbalance in the model group directly led to insufficient SCFA production: butyrate deficiency impaired intestinal epithelial energy supply and barrier function, increased the release of inflammatory factors (e.g., TNF-α), and caused enteric nervous system (ENS) damage; acetate deficiency inhibited GPR43 receptor activation, reduced enteric nerve 5-HT release, and weakened intestinal peristalsis. This SCFA synthesis defect further induced ENS synaptic transmission abnormalities and smooth muscle “contraction-relaxation” imbalance, which, combined with enteric nerve inflammatory damage, formed a vicious cycle of “motility insufficiency–inflammatory injury.” RH intervention (low-, medium-, high-dose, and positive control groups) significantly increased the proportions of *Eubacteriales* and *Lachnospirales*, with the medium-dose group exerting the optimal effect: abundances of both taxa were synchronously elevated to near-normal levels. Restoration of SCFA-producing microbiota reestablished acetic acid and butyric acid synthesis homeostasis: acetic acid promoted 5-HT release via GPR43 receptor activation to enhance peristalsis, while butyric acid repaired the intestinal epithelial barrier and inhibited inflammation to reduce ENS damage, ultimately improving ENS synaptic transmission, rebalancing smooth muscle contraction, and restoring intestinal motility.

At the species level, alterations in the relative abundances of core genera further validated these regulatory trends (Figure 6D). Compared to the control and treatment groups, the model group showed significantly reduced relative abundances of *Ruminococcus* sp., *Eubacterium* sp., *Blautia* sp., and *Oscillibacter* sp. After RH intervention, the abundance of *Ruminococcus* sp. was significantly increased: this genus exhibits active carbohydrate fermentation, degrading complex starch into small molecules and producing SCFAs (e.g., acetate, propionate) to support intestinal peristalsis. The abundance of *Eubacterium* sp. was also elevated: as a core butyrate-producing genus, its metabolite butyrate serves as the primary energy source for intestinal epithelial cells, repairs the intestinal barrier, inhibits inflammation, and promotes 5-HT release via GPR43 receptor activation, synergistically enhancing intestinal motility. Meanwhile, the abundance of *Blautia* sp. was decreased in intervention groups: overproliferation of this genus consumes SCFA precursors (e.g., dietary fiber) or produces motility-inhibiting substances (e.g., branched-chain amino acid derivatives), and its downregulation relieved intestinal motility inhibition, further alleviating constipation.

Linear discriminant analysis (LDA) combined with effect size analysis (LEfSe) was used to screen for microbial biomarkers with biological significance that differed among groups. Core biomarkers were identified via LDA value distribution bar plots and species cladograms (Figures 6E,F). Results showed distinct differentiation of core functional taxa: the control group was dominated by organic matter-degrading *Bathyarchaeia*, with broad microbiota distribution and high diversity; the model group was characterized by unclassified *Nanoarchaeia*; the high-dose group enriched diverse *Nanoarchaeia*; the medium-dose group centered on hydrogenotrophic methanogenic *Methanomicrobia*; the low-dose group favored acetic acid-type methanogenic *Methanofastidiosales*; and the positive control group integrated methanogenic and nitrogen-cycling taxa. Except for the control group, all other groups exhibited specialized microbiota structures due to functional taxon enrichment, with diversity concentrated in specific metabolic pathways, further confirming the targeted regulatory effect of RH on microbiota function. The species association network (Figure 6G) further visualized the co-occurrence patterns of core taxa: the control group showed dense, multi-directional species interactions, while the model group exhibited sparse, unidirectional associations; RH intervention (especially medium-dose) restored the complexity of species co-occurrence networks, consistent with the recovery of microbiota diversity.

### 3.6 Effect of RH on serum metabolomics in STC rats

**Figure 7:**
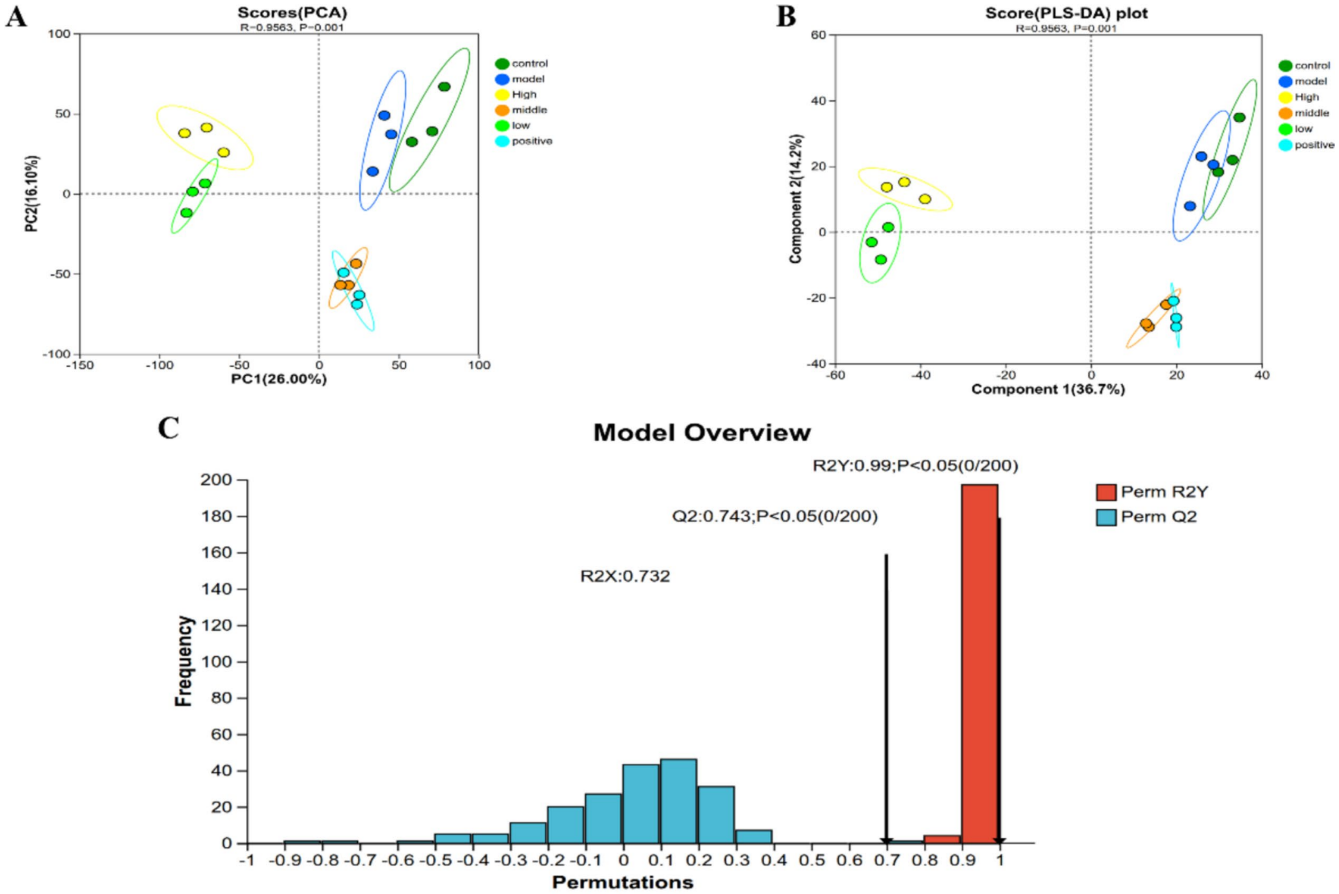
Multivariate statistical analysis of serum metabolomics in STC rats following RH intervention. **(A)** PCA score plot, **(B)** PLS-DA score plot. The *R* value ranges from −1 to 1. A smaller *R* value indicates no significant differences between and within groups, and the *p* value is an indicator of the statistical significance of such differences, **(C)** permutation test results. The *x*-axis represents the accuracy of random models in the permutation test, and the *y*-axis represents the number of random models. Red bars indicate the number of occurrences of the *Q*^2^ value derived from the permutation test, and blue bars indicate the number of occurrences of the *R*^2^*Y* value derived from the permutation test. *R*^2^*X* and *R*^2^*Y* represent the interpretation rates of the constructed model for the *X* and *Y* matrices, respectively; *Q*^2^ denotes the predictive ability of the model. The closer these three metrics are to 1, the more stable and reliable the model is. A *Q*^2^ value >0.5 indicates good predictive ability of the model, while a *Q*^2^ value <0.5 indicates poor predictive ability of the model.

Multivariate statistical analysis, including principal component analysis (PCA), partial least squares-discriminant analysis (PLS-DA), and permutation test, was performed on serum metabolomics data to verify the intervention effect of RH on metabolic disorders in STC rats. Results showed that RH exerted a significant regulatory effect on metabolic perturbations, with the medium-dose group exhibiting the most favorable pharmacological activity (Figure 7).

Unsupervised PCA (Figure 7A) revealed distinct separation of sample clusters between the control and model groups, indicating marked disruption of serum metabolic profiles induced by STC modeling. Sample clusters of all treatment groups showed a tendency to cluster toward the control group with good intra-group aggregation, demonstrating that all RH doses effectively reversed STC-associated metabolic abnormalities. Notably, sample point clouds of the medium-dose RH group and positive control group overlapped substantially, preliminarily suggesting similar metabolic restoring effects between the two groups.

Supervised PLS-DA (Figure 7B) further amplified inter-group metabolic differences: sample point clouds of the model and control groups were completely separated, while the medium-dose RH group and positive control group, though closely clustered, displayed subtle distinctions. This not only confirmed the efficacy of RH intervention but also implied both commonalities and specificities in the metabolic regulatory pathways between medium-dose RH and the positive drug.

Permutation test results (Figure 7C) showed that the PLS-DA model yielded *R*^2^*Y* = 0.99 and *Q*^2^ = 0.743, with *Q*^2^ values of permuted models distributed near 0 or in the negative range. This indicated no model overfitting and confirmed good stability and reliability of the PLS-DA model, providing robust statistical support for interpreting inter-group metabolic differences.

Collectively, these findings demonstrate that RH alleviates STC symptoms through multi-dimensional regulation of serum metabolic profiles, with the medium-dose group achieving metabolic restoration most closely resembling that of the positive drug (thus representing the optimal dose). The subtle differences in metabolic profiles between the medium-dose RH group and positive control group also provide a basis for exploring the unique “multi-target regulatory” mechanism of RH.

#### 3.6.1 Screening of differential metabolites associated with RH’S laxative effect

Differential metabolites were screened based on PLS-DA results using the following criteria: variable importance in projection (VIP) > 1, *p* < 0.05, and fold change (FC) > 1. Serum metabolites were analyzed in both positive and negative ion modes to identify statistically significant differential metabolites among groups.

**Figure 8:**
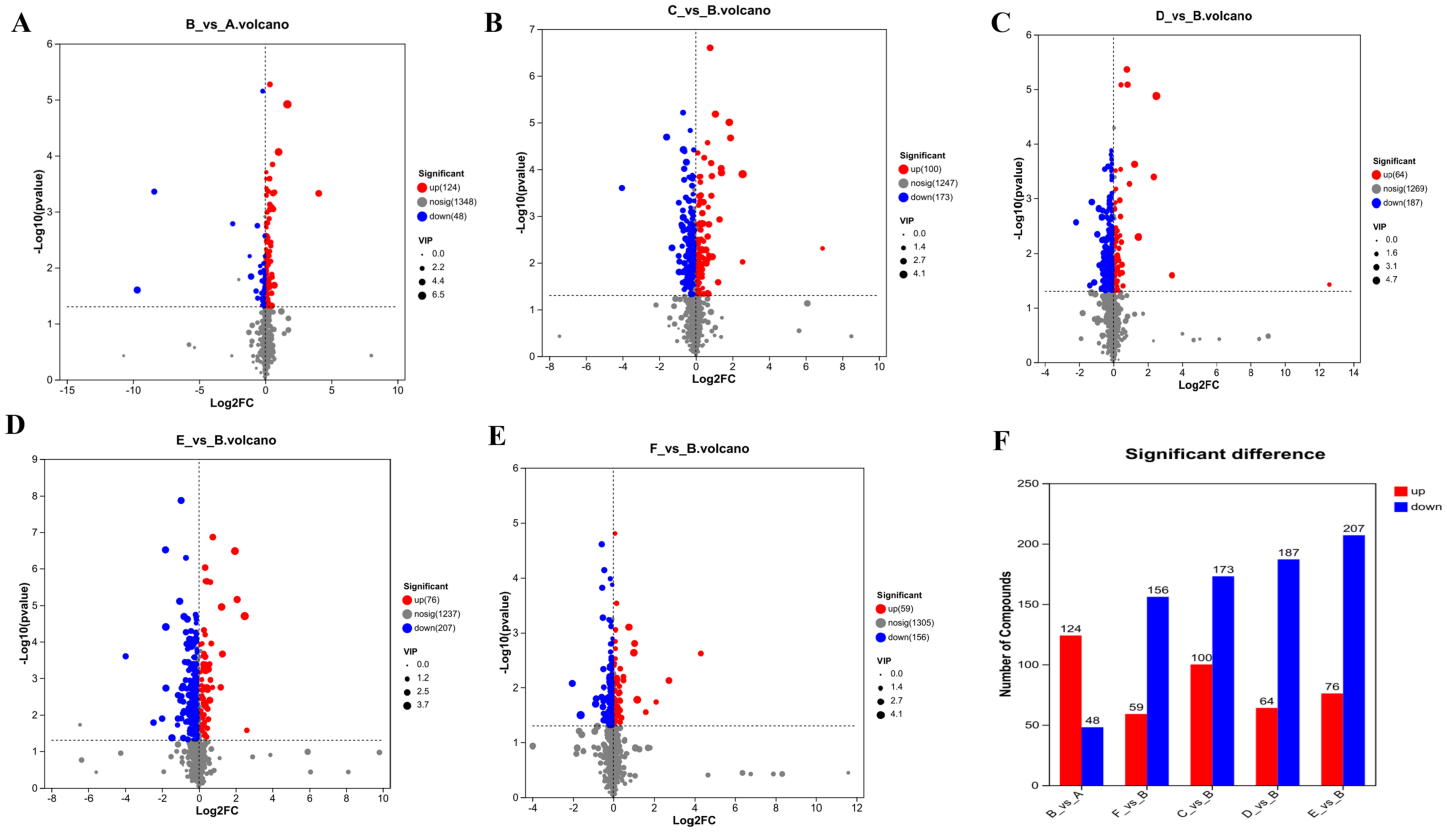
Volcano plot of differential metabolites. **(A)** Model vs. control volcano plot, **(B)** high-dose RH vs. model volcano plot, **(C)** medium-dose RH vs. model volcano plot, **(D)** low-dose RH vs. model volcano plot, **(E)** positive control vs. model volcano plot, **(F)** summary of significant differential metabolites (upregulated/downregulated).

As shown in Figures 8A–F, a total of 172 differential metabolites associated with the laxative effect of RH were identified between the model and control groups (124 upregulated, 48 downregulated); 273 between the high-dose RH group and model group (100 upregulated, 173 downregulated); 251 between the medium-dose RH group and model group (64 upregulated, 187 downregulated); 283 between the low-dose RH group and model group (76 upregulated, 207 downregulated); and 215 between the positive control group and model group (59 upregulated, 156 downregulated).

Among these differential metabolites, 106 were shared among the low-, medium-, and high-dose RH groups, and 45 were common to the positive control group and all RH dose groups. Intersection analysis between these 45 metabolites and those in the control group identified 15 key differential metabolites (Table 1).

**Table 1:** Results of differential metabolites.

Number	Metabolite	Metab ID	Control vs. model	Model vs. High	Model vs. Middle	Model vs. Low	Model vs. Positive
1	N-desmethyl-loperamide	metab_7595	↑###	↓**	↓**	↓**	↓**
2	Loperamide	metab_7419	↑#	↓***	↓**	↓**	↓**
3	3-[4-(sulfooxy)phenyl]propanoic acid	metab_22049	↑#	↓**	↓**	↓**	↓*
4	P-sulfoxy-cinnamic acid	metab_21988	↑#	↓*	↓**	↓**	↓*
5	Estetrol	metab_11528	↓#	↑**	↑*	↑*	↑*
6	Jasmine lactone	metab_1721	↑#	↓*	↓*	↓*	↓*
7	Metkephamid	metab_21210	↓#	↑***	↑***	↑**	↑**
8	Favipiravir	metab_23447	↑#	↓**	↓**	↓**	↓**
9	(3R,4R,7R,8R,12R)-ent-3,4:7,8-Diepoxyverticillan-12-ol	metab_17074	↑#	↓**	↓**	↓***	↓*
10	Ascr#11	metab_22198	↑#	↓*	↓*	↓**	↓*
11	N-Acetylserotonin	metab_9641	↑#	↓**	↓**	↓***	↓**
12	4-Guanidinobutanoic acid	metab_10762	↑#	↓***	↓***	↓***	↓*
13	(+/−)7-HDoHE	metab_4778	↑#	↓**	↓**	↓***	↓*
14	15-Hydroxydehydroabietic acid	metab_3569	↑#	↓***	↓**	↓***	↓*
15	L-Glutamate	metab_485	↑##	↓**	↓*		↓**

### 3.7 Pathway enrichment analysis of regulated differential metabolites

**Figure 9:**
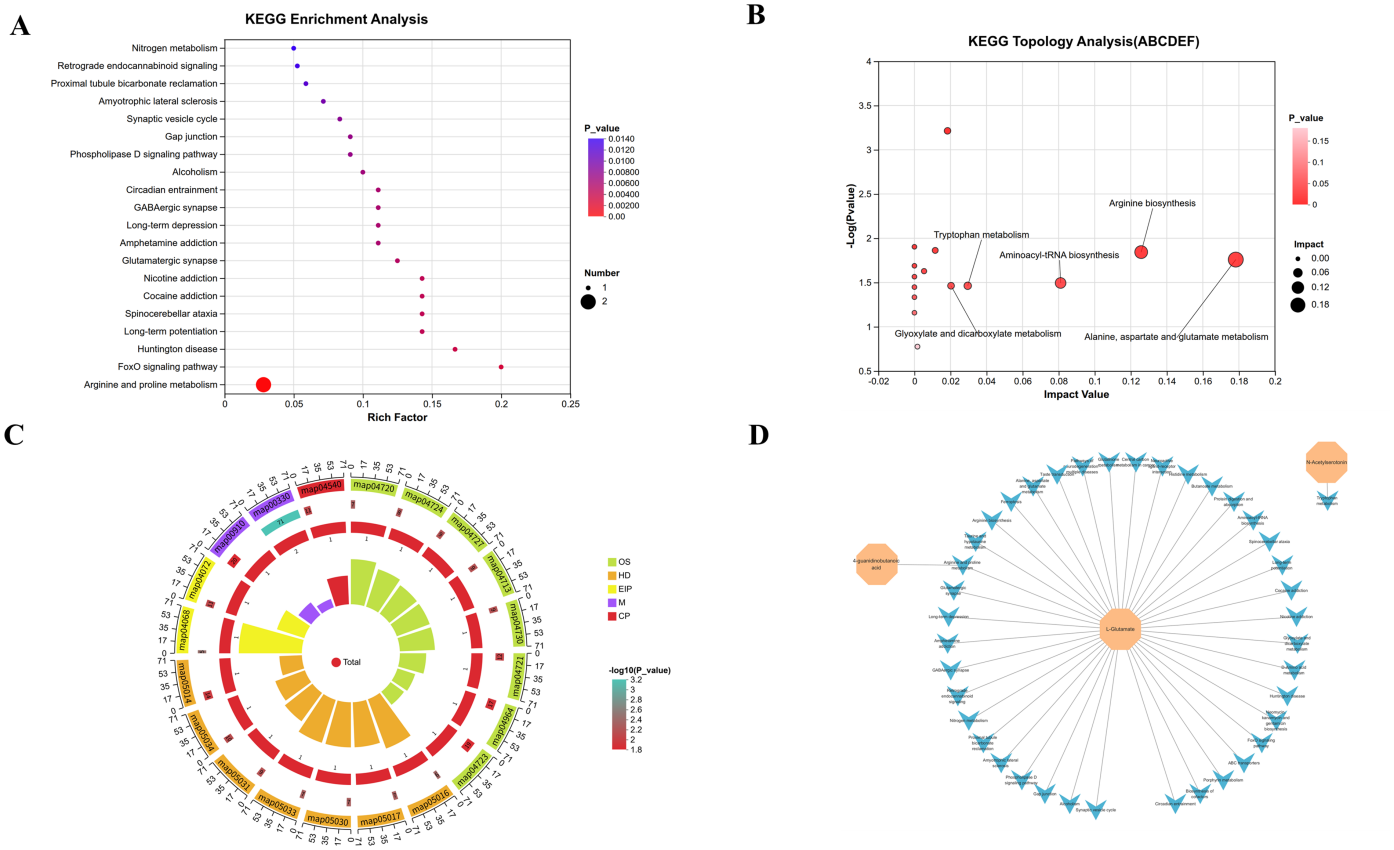
KEGG enrichment and network analysis of serum differential metabolites in STC rats. **(A)** KEGG enrichment bubble plot, **(B)** KEGG enriched pathway circular plot. The *x*-axis represents the enrichment factor (Diff/Background) of different omics in this pathway, while the *y*-axis represents the names of KEGG pathways. The red-blue gradient indicates the degree of enrichment from high to low, as represented by *p*-value. The shape of bubbles represents different omics, and the size of bubbles represents the number of differential metabolites or genes, with larger bubbles indicating a greater quantity, **(C)** KEGG topological analysis scatter plot, **(D)** metabolite-pathway correlation network diagram. Orange octagons represent key differential metabolites, while blue triangles represent KEGG-enriched pathways.

KEGG enrichment analysis of the 15 key differential metabolites identified 40 significantly enriched pathways, with the top 20 pathways visualized using bubble plots (Figures 9A–D). The metabolite-pathway correlation network (Figure 9D) constructed a multi-layered regulatory network centered on L-glutamate, encompassing amino acid metabolism, neurotransmitter signaling, and intestinal motility regulation. As the primary energy substrate for intestinal epithelial cells and a key excitatory neurotransmitter in the ENS, dysregulation of L-glutamate metabolism directly impairs ENS synaptic transmission, leading to intestinal hypoperistalsis. Here, L-glutamate was identified as a central hub linked to alanine-aspartate–glutamate metabolism (map00250), arginine biosynthesis (map00220), and glutamatergic synapse (map04724), highlighting its pivotal role in RH-mediated reversal of STC-associated metabolic dysregulation.

Notably, N-acetylserotonin (a tryptophan metabolite) was associated with tryptophan metabolism (map00380) and serotonergic synapse (map04726), confirming that RH modulates the tryptophan-5-hydroxytryptamine (5-HT) axis to enhance intestinal motility. Given that insufficient 5-HT secretion is a hallmark of STC, RH indirectly elevates intestinal 5-HT levels by facilitating the conversion of tryptophan to N-acetylserotonin, thereby amplifying peristaltic signaling. Furthermore, 4-guanidinobutyric acid was linked to arginine and proline metabolism (map00330), indicating that RH modulates intestinal smooth muscle tone by driving the conversion of arginine to 4-guanidinobutyric acid. As a precursor of γ-aminobutyric acid (GABA), 4-guanidinobutyric acid acts via GABAergic synapses to suppress excessive smooth muscle contraction, alleviating motility retardation in STC rats.

KEGG topological analysis (Figure 9C) prioritized the core regulatory pathways of RH in STC based on pathway centrality (Impact Value) and enrichment significance [−log₁₀(*p*-value)]. Alanine-aspartate–glutamate metabolism emerged as the most central pathway (highest Impact Value), underscoring its dominant hub role in the global metabolic network. Activation of this pathway enhances intestinal epithelial energy metabolism and barrier integrity by increasing glutamine availability, mitigating epithelial damage in STC rats. Enrichment of arginine biosynthesis further delineated a key motility regulation mechanism: RH enhances arginine biosynthesis to promote nitric oxide (NO) production, which relaxes intestinal smooth muscle and facilitates propulsive motility. Since impaired NO synthesis is a major contributor to STC-associated motility dysfunction, RH-mediated activation of arginine biosynthesis directly restores NO bioavailability. Additionally, enrichment of tryptophan metabolism confirmed RH’s regulation of the 5-HT pathway, providing metabolomic evidence for its pharmacological feature of “multi-target modulation of intestinal motility.”

Hierarchical visualization via the KEGG enriched pathway circular plot (Figure 9B) revealed distinct differences in pathway enrichment profiles between the STC model group and RH-treated groups. The model group exhibited enrichment in neurodegenerative disease pathways [e.g., Huntington’s disease (map05016), spinocerebellar ataxia (map05014)], indicating enteric neurodegenerative-like metabolic dysfunction in STC rats—which is consistent with well-established STC pathogenesis, where ENS degeneration drives motility impairment. In contrast, RH-treated groups were enriched in amino acid metabolism (map00220, map00250) and synaptic signaling pathways (map04724, map04727), demonstrating that RH reverses neurodegenerative metabolic dysfunction by restoring amino acid homeostasis and synaptic transmission, thereby re-establishing ENS regulatory control. These inter-group pathway differences further validate RH’s targeting efficacy, providing direct evidence for its “ENS function restoration” mechanism.

Collectively, RH intervenes in STC primarily via an axis-based mechanism: “amino acid metabolic reprogramming—neurotransmitter signal activation—intestinal motility restoration.” Specifically, RH reprograms the amino acid metabolic network by activating alanine-aspartate–glutamate metabolism and arginine biosynthesis, enhancing intestinal epithelial energy supply and NO production capacity; modulates neurotransmitter release and restores intestinal nerve regulation by targeting the tryptophan-5-HT pathway and glutamatergic/GABAergic synaptic signals; and alleviates intestinal motility retardation by repairing the intestinal epithelial barrier and regulating smooth muscle tone.

### 3.8 Combined analysis of metabolomics and metagenomics

**Figure 10:**
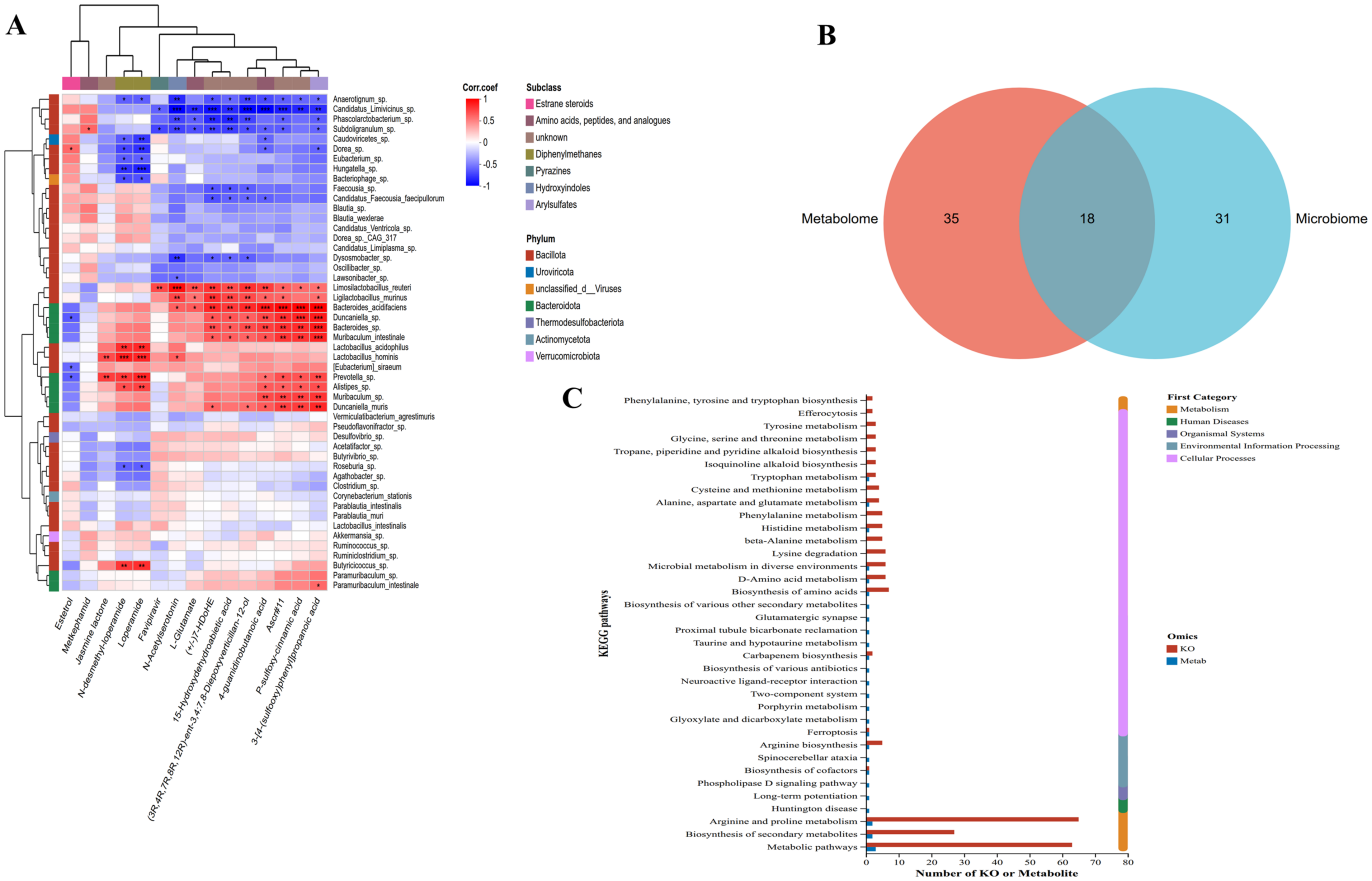
Multi-omics correlation analysis of microbiota-metabolite interactions in STC rats following RH intervention. **(A)** Spearman correlations between metabolites and the gut microbial communities at the species level. Red = positive correlation, blue = negative correlation; color intensity reflects correlation coefficient magnitude, ***p < 0.001, **p < 0.01, *p < 0.05, Kruskal–Wallis *H* test; **(B)** Venn diagram showing the numbers of common and specific pathways among metabolome and microbiome; **(C)** KEGG pathway enrichment bar plot (metabolome vs. microbiome).

The Spearman correlation heatmap (Figure 10A) constructed a fine-scale interaction network To characterize the associations between metabolites and gut microorganisms, Spearman’s rank correlation analysis (Figure 9) was performed, with results visualized as a clustered heatmap.

In the heatmap, L-glutamate exhibited strong positive correlations with *Bacillota*-phylum genera (e.g., *Ligilactobacillus* reuteri, *Bacteroides dorei*). This correlation indicates that these genera enhance L-glutamate biosynthesis by expressing glutamate dehydrogenase: as the primary energy substrate for intestinal epithelial cells and a key excitatory neurotransmitter in the ENS, elevated L-glutamate levels directly strengthen ENS synaptic transmission—reversing the hypoperistalsis caused by glutamate metabolic dysregulation in STC rats.

Similarly, 4-guanidinobutyric acid showed a significant positive correlation with *Ligilactobacillus* reuteri. This association implies that *L. reuteri* facilitates the conversion of arginine to 4-guanidinobutyric acid: as a precursor of γ-aminobutyric acid (GABA), 4-guanidinobutyric acid acts via GABAergic synapses to suppress excessive intestinal smooth muscle contraction, thereby alleviating motility retardation in STC models. These positively correlated microbial-metabolite modules directly validate the mechanism by which gut microbiota modulate metabolite production to improve intestinal motility.

In contrast, N-acetylserotonin (a tryptophan metabolite) displayed a strong negative correlation with *Anaerotruncus* sp. This negative association suggests that *Anaerotruncus* sp. inhibits tryptophan hydroxylase activity, reducing the conversion of tryptophan to N-acetylserotonin (a precursor of 5-HT). RH intervention alleviates this inhibition by decreasing *Anaerotruncus* sp. abundance, indirectly elevating intestinal N-acetylserotonin levels and subsequent 5-HT secretion—strengthening peristaltic signaling in STC rats.

Additionally, loperamide (the STC model inducer) showed a significant positive correlation with *Dorea* sp., indicating that *Dorea* sp. abundance increases in response to loperamide exposure and contributes to model-induced metabolic dysregulation. RH reverses this dysbiosis by downregulating Dorea sp. abundance, restoring metabolic homeostasis.

From the perspective of metabolite subclass clustering, the “Amino acids, peptides, and analogues” subclass showed the closest associations with *Bacillota*-phylum genera, confirming that amino acid metabolism is the core module of microbiota-metabolite interactions. In contrast, diphenylmethanes were specifically correlated with *Actinobacteriota*-phylum genera, suggesting this metabolite class regulates the secondary metabolism of gut microbiota. This subclass-phylum specificity refines the microbiota-metabolite interaction network, providing a precise target framework for RH-mediated regulation.

A Venn diagram of differential pathways between the metabolome and microbiome (Figure 10B) revealed 35 metabolome-unique pathways, 31 microbiome-unique pathways, and 18 co-enriched pathways (total 84 pathways). Most single-omics studies focus on independent regulation of either omics layer, but this study quantified the contribution of microbiota-metabolite interactions to RH intervention (18/84, 21.4%)—the first report of such a synergistic regulation ratio for RH.

The KEGG pathway enrichment bar plot (Figure 10C) further distinguished regulatory patterns between the metabolome (Metab, blue bars) and microbiome (KO, red bars) across functional categories. Unlike previous studies that focused on either neurotransmitter signaling (metabolome) or microbial metabolism (microbiome), this analysis identified “Metabolism” as the most prominently co-enriched category (62.5% of co-enriched pathways). For example, in arginine and proline metabolism, the microbiome contained ~70 differential genes (far more than the metabolome’s ~ 10 differential metabolites), confirming that gut microbiota act as upstream regulators of this pathway—consistent with RH’s ability to enhance arginine biosynthesis (via microbiota) to promote NO production and smooth muscle relaxation.

## 4 Discussion

STC is a refractory subtype of constipation characterized by delayed colonic transit, with a prolonged, recurrent course that severely impairs patients’ quality of life (Mingmin, 2020; Robin et al., 2018). Modern medical management of STC primarily relies on basic therapies, which are limited in scope and yield poor long-term outcomes (Koh et al., 2023; Lund et al., 2018). TCM posits that STC is localized to the large intestine, with pathogenesis centered on colonic transmission dysfunction. Accumulating evidence confirms that TCM-based interventions for STC offer advantages such as definite efficacy, safety, reliability, and sustained effects (Barbara et al., 2016; Zhao and Yu, 2016).

Gastrointestinal hormones are potent bioactive substances secreted by scattered endocrine cells in the gastrointestinal wall and pancreatic islet cells, with core functions in regulating gastrointestinal motility. Key gastrointestinal hormones include GAS, MTL, VIP, secretin, and SP. As polypeptides, these hormones are also referred to as gastrointestinal peptides; some are distributed in both the central nervous system and enteric nervous system (ENS), hence the term “brain-gut peptides.” To date, 20 brain-gut peptides have been identified (including GAS, SP, and somatostatin), and their dual distribution enables critical physiological roles in regulating animal gastrointestinal motility. SP, a neuropeptide, directly acts on intestinal smooth muscle in the gastrointestinal tract to promote peristalsis and accelerate intestinal content excretion. Elevated SP levels not only enhance intestinal peristalsis but also reduce gastrointestinal water absorption, increase fecal water content and volume, and shorten intestinal residence time, thereby alleviating constipation (Jin-hui et al., 2018). However, conflicting evidence suggests that elevated SP levels may exacerbate constipation in STC patients (Lingling, 2014). MTL, the most common gastrointestinal hormone, is a major excitatory peptide regulating gastrointestinal motility, named for its stimulatory effects on gastric and intestinal motility. MTL primarily increases intracellular inositol trisphosphate (IP3) concentration and cytosolic calcium levels, stimulating contractions of the gastric body and lesser curvature to promote gastric peristalsis, accelerate gastric emptying, enhance colonic peristaltic capacity, and increase gastrointestinal content transit speed—playing a key role in gastrointestinal motility disorders (Meijun et al., 2012).

In the present study, STC modeling resulted in decreased serum GAS and MTL levels and increased SP levels in rats. Following intervention with RH or mosapride, SP levels gradually decreased to normal, while MTL and GAS levels were restored to physiological ranges, confirming RH’s laxative effect.

Metabolomic analysis identified 15 differential serum metabolites across groups, with RH modulating pathways including nitrogen metabolism, retrograde endocannabinoid signaling, proximal tubule bicarbonate reabsorption, and the phospholipase D-signaling pathway. Abnormal arginine and proline metabolism is a well-recognized etiological factor in STC (Xie et al., 2021; Yan et al., 2020). Here, RH intervention increased serum levels of glutamate and 4-guanidinobutyric acid (terminal metabolites of arginine and proline metabolism) in STC rats, suggesting RH ameliorates arginine-proline metabolic dysregulation. Additionally, nitrogen metabolism abnormalities are closely linked to STC (Nengsheng and Chunyu, 2009; Tetens et al., 1996; Xie et al., 2021), as are tryptophan metabolism disorders (Xiaoyi, 2021) and taurine-hypotaurine metabolism abnormalities (Jinjiang et al., 2023).

Metagenomic analysis revealed distinct gut microbiota characteristics among the 6 groups. At the species level, RH reduced the abundance of multiple harmful bacteria and enriched beneficial bacteria. RH intervention significantly increased *Ruminococcus* sp. abundance in constipated rats—consistent with Li et al. (2025)—indicating this genus exhibits active carbohydrate fermentation, degrading complex starch into simple molecules. *Ruminococcus* sp. plays a key role in digesting starch, maltose, fucosylated glycans, and other substrates, producing acetate, propionate, formate, and ethanol (but not butyrate). *Eubacterium* sp. abundance was also elevated following RH treatment, aligning with Mukherjee et al.’s (2020) findings. Multiple *Eubacterium* species produce butyrate, a key metabolite involved in energy homeostasis, colonic peristalsis, immune regulation, and intestinal inflammation inhibition. Guo et al. (2011) identified *Bacteroides* sp. as a disease-associated flora (linked to ulcerative colitis and colorectal cancer), with significantly increased abundance in the model group and reduced levels after RH intervention. *Blautia* sp., a common gut genus, exhibits “dual-sided” roles in constipation: while some studies suggest it may act as a probiotic (decreased in elderly and colorectal cancer patients, increased in IBS patients) (Liu et al., 2021), its elevated abundance in constipated patients may indirectly exacerbate constipation by excessive consumption of SCFA precursors (e.g., dietary fiber) or production of motility-inhibiting substances (e.g., branched-chain amino acid derivatives). Other studies highlight its potential to promote carbohydrate metabolism, emphasizing the need for species-specific analysis (Liu et al., 2021). Furthermore, growing evidence links chronic constipation to intestinal inflammation relief and intestinal mucosal protection, with correlations to disease progression (Chen et al., 2020; Liu et al., 2020; Vandeputte et al., 2016).

In the current study, *Eubacteriales* and *Lachnospirales* were the dominant gut taxa—core flora for SCFA (butyrate and acetate) synthesis. SCFAs directly improve constipation by promoting intestinal peristalsis, regulating intestinal neurotransmitters (e.g., 5-HT), and maintaining intestinal barrier integrity (Fenglian and Zhifeng, 2025; Firth et al., 2025; Mukherjee et al., 2020; Vacca et al., 2020; Ying et al., 2022). Compared with the control group, the model group showed decreased *Eubacteriales*/*Lachnospirales* abundance (corresponding to the constipation phenotype), while RH intervention (particularly the medium-dose group) restored their abundances to normal levels—suggesting RH exerts laxative effects via gut microbiota modulation.

The study found that nitrogen metabolism and arginine and proline metabolism were significantly enriched (low *p*-values). Arginine metabolism can generate nitric oxide (NO), a key signaling molecule for intestinal peristalsis (capable of relaxing intestinal smooth muscle and promoting intestinal motility).

Nitrogen metabolism is associated with microbial protein decomposition and SCFA synthesis (indirectly affecting the intestinal environment). Arginine biosynthesis and tryptophan metabolism are key pathways: tryptophan metabolism can generate 5-HT, a core neurotransmitter regulating intestinal peristalsis (constipated patients often have reduced 5-HT levels); arginine biosynthesis supports NO production, further promoting intestinal motility. Changes in microbiota structure (restoration of Eubacteriales/Lachnospirales abundance) activate arginine/tryptophan metabolic pathways, generating intestinal motility molecules such as NO and 5-HT, which promote intestinal peristalsis and improve constipation. These findings indicate that these specific genera may play critical roles in the pathophysiology of functional constipation.

### 4.1 Limitations and future directions

Although this study has preliminarily revealed the mechanism by which RH exerts anti-STC effects by regulating intestinal microecology, alleviating metabolic pathway disorders, and modulating gastrointestinal hormone levels—using animal experiments, metabolomics, and metagenomics techniques—it has several notable limitations that need to be addressed in subsequent research.

First, the small sample size is a major limitation. The sample size of rats in each group was only 3. Although data reliability was ensured through quality control sample validation and repeated measurements, the small sample size may reduce statistical power. Particularly in gut microbiota differential analysis and metabolite correlation analysis, it may lead to detection bias for some low-abundance differential bacteria or metabolites, affecting the stability and reproducibility of the results. Future studies should expand the animal sample size and conduct multiple independent batches of experiments to verify the generalizability of the conclusions.

Second, the validation of underlying mechanisms is insufficiently in-depth. Based on multi-omics correlation analysis, this study speculates that RH may exert its effects by regulating the abundances of Eubacteriales and Lachnospirales, activating arginine/tryptophan metabolic pathways, and modulating molecules such as 5-HT and NO. However, direct mechanism validation experiments are lacking. For example, fecal microbiota transplantation (FMT), specific genus knockout, or pathway inhibitor intervention were not used to clarify the causal role of core microbiota (e.g., *Ruminococcus* sp., *Eubacterium* sp.) or key metabolic pathways (e.g., arginine-proline metabolism) in the anti-STC effect of RH. Meanwhile, the specific targets and molecular regulatory networks of active components in RH (e.g., terpenoids, lipids) have not been clarified, and the complete “component-microbiota-pathway-pharmacodynamic” regulatory chain remains unelucidated. In the future, *in vitro* cell experiments, genetically edited animal models, and molecular interaction technologies should be combined to further validate the causal relationships of the core mechanisms.

Finally, there is a lack of support from clinical translation research. This study was conducted using an STC rat model, and significant differences exist in pathophysiological status between animal models (e.g., single-factor induced constipation, standardized feeding environment) and clinical STC patients. Clinical patients often exhibit characteristics such as dietary heterogeneity, comorbidities (e.g., diabetes mellitus, anxiety, depression), and more complex gut microbiota diversity. Additionally, this study did not explore the safety, tolerability, or optimal dosage of RH in humans. Therefore, the current research conclusions cannot be directly extended to clinical applications. Future studies should conduct well-designed clinical cohort studies, enroll STC patients with different disease courses and subtypes, and systematically evaluate the clinical efficacy, adverse reactions of RH, as well as its effects on patients’ intestinal microecology and metabolic profiles—thus providing evidence-based medical evidence for its clinical translation.

Although the above limitations do not affect the reliability of the core conclusions of this study, they point out directions for future research. In the future, the theoretical system of RH against STC can be further improved by expanding the sample size, deepening mechanism validation, and promoting clinical translation, providing a more solid scientific basis for its development as a new therapeutic drug or functional food.

## Data Availability

The multi-omics data generated in this study have been deposited in relevant public databases. The metabolomics data are available in the MetaboLights database under accession number MTBLS13888. The metagenomic sequencing data are available in the NCBI SRA database under Bioproject number PRJNA1422082.

## Footnotes

1
https://metlin.scripps.edu/
