# Supplementary material for: Based on untargeted metabolomics and metagenomics: a study on the mechanism of Miao ethnomedicine Zingiber mioga (Thunb.) Rosc. in treating slow transit constipation
Source: Front Microbiol. 2026 Feb 27;17:1751739. doi: 10.3389/fmicb.2026.1751739 (PMC12982368; doi:10.3389/fmicb.2026.1751739)
Supplement: Supplementary file 1 [file Supplementary_file_1.pdf]

**审批号：20251111002**

# 贵州中医药大学实验动物伦理审查委员会审批表

**申请日期：2025年11月11日**

| | | | | | |
|---|---|---|---|---|---|
| 项目名称 | 基于苗药数据库构建对蘘荷安全性评价及减肥产品开发和那嘎青治疗癌症疼痛的网络药理学及代谢组学作用机制研究 | | | | |
| 动物实验执行人 | 杜宇涛 | 职称/学位 | 在读研究生/理学 | 邮箱 | 1937292216@qq.com |
| 项目负责人 | 胡成刚 | 职称/学位 | 教授/理学 | 邮箱 | 2274547063@qq.com |
| 申请单位及专业 | 贵州中医药大学药学院23级中药学专硕班 | | | | |
| 动物种系 | SD大鼠、KM小鼠 | 数量 | 112 | | |
| 实验种类 | 医学研究 | | | | |
| 审查依据 | 1、该项目是否必须用实验动物进行实验，即能否用计算机模拟、细胞培养等非生命方法替代动物或用低等动物替代高等动物进行实验。<br>2、表中所填实验相关人员资格和实验相关单位是否合适。<br>3、表中所填实验所用动物能否通过改良设计方案或用高质量的动物来减少所用动物的数量。<br>4、能否通过改进实验方法、调整实验观测指标、改良处死动物的方法，来优化实验方案、善待动物 。<br>5、实验设计、实验技术方法及用于本实验的动物数量是否合理可行。 | | | | |
| 伦理委员1审查意见 | 周谊霞 驳回 我校不用戊巴比妥钠作为麻醉药，需更换一下 | | 伦理委员2审查意见 | 王飞清 同意 | |
| 学校实验动物伦理委员会审查意见 | 同意 | | | | |
| 主任委员/副主任委员 | 吴曙光 | 审查日期 | 2025年11月19日 | | |

贵州中医药大学实验动物伦理审查委员会
