# Supplementary material for: Based on untargeted metabolomics and metagenomics: a study on the mechanism of Miao ethnomedicine Zingiber mioga (Thunb.) Rosc. in treating slow transit constipation
Source: Front Microbiol. 2026 Feb 27;17:1751739. doi: 10.3389/fmicb.2026.1751739 (PMC12982368; doi:10.3389/fmicb.2026.1751739)
Supplement: Supplementary file 3 [file Table_2.docx]

Supplementary Material

# Supplementary Data

| 样品编号 | P物质（SP）（pg/ml） | 胃动素（MTL）（pg/ml） | 胃泌素（Gas）（pg/ml） |
| --- | --- | --- | --- |
| 正雄1 | 48.19 | 413.49 | 421.29 |
| 正雄3 | 59.53 | 403.82 | 447.02 |
| 正雄4 | 49.66 | 393.70 | 465.01 |
| 模雄1 | 109.19 | 97.97 | 147.42 |
| 模雄3 | 118.79 | 50.03 | 139.30 |
| 模雄5 | 103.16 | 96.21 | 162.16 |
| 低雄1 | 81.99 | 244.00 | 276.61 |
| 低雄2 | 86.23 | 305.00 | 238.63 |
| 低雄4 | 71.69 | 309.98 | 314.96 |
| 中雄2 | 71.55 | 250.16 | 292.22 |
| 中雄5 | 84.59 | 298.10 | 340.58 |
| 中雄7 | 63.85 | 267.61 | 252.74 |
| 高雄2 | 62.98 | 243.86 | 325.71 |
| 高雄3 | 58.32 | 224.94 | 365.69 |
| 高雄5 | 58.23 | 306.31 | 360.82 |
| 阳雄1 | 63.51 | 331.68 | 295.35 |
| 阳雄3 | 53.89 | 361.30 | 350.07 |
| 阳雄6 | 72.94 | 372.29 | 262.11 |

|  | SP（pg/ml） | MTL（pg/ml） | Gas（pg/ml） |  |
| --- | --- | --- | --- | --- |
| CON | 52.46±6.17 | 403.67±9.9 | 444.44±21.97 |  |
| MOD | 110.38±7.88 | 81.4±27.18 | 149.63±11.59 |  |
| RH-L | 79.97±7.48 | 286.33±36.74 | 276.73±38.17 |  |
| RH-M | 73.33±10.48 | 271.96±24.26 | 295.18±43.99 |  |
| RH-H | 59.84±2.72 | 258.37±42.58 | 350.74±21.81 |  |
| PC | 63.45±9.53 | 355.09±21.01 | 302.51±44.41 |  |

|  | Mucosal layer thickness（μm） | Crypt depth（μm） |
| --- | --- | --- |
| mod | 202.81 | 160.78 |
|  | 187.52 | 140.35 |
|  | 252.96 | 162.20 |
| con | 219.97 | 203.99 |
|  | 305.47 | 182.56 |
|  | 277.84 | 202.89 |
| RH-Low | 244.57 | 150.71 |
|  | 213.67 | 174.71 |
|  | 235.47 | 182.04 |
| RH-Middle | 308.71 | 179.37 |
|  | 237.03 | 161.44 |
|  | 296.43 | 160.51 |
| RH-High | 282.96 | 182.38 |
|  | 272.87 | 171.43 |
|  | 280.99 | 163.03 |
| PC | 371.60 | 194.52 |
|  | 359.98 | 220.79 |
|  | 313.02 | 245.91 |

# Supplementary Figures and Tables

## Supplementary Figures


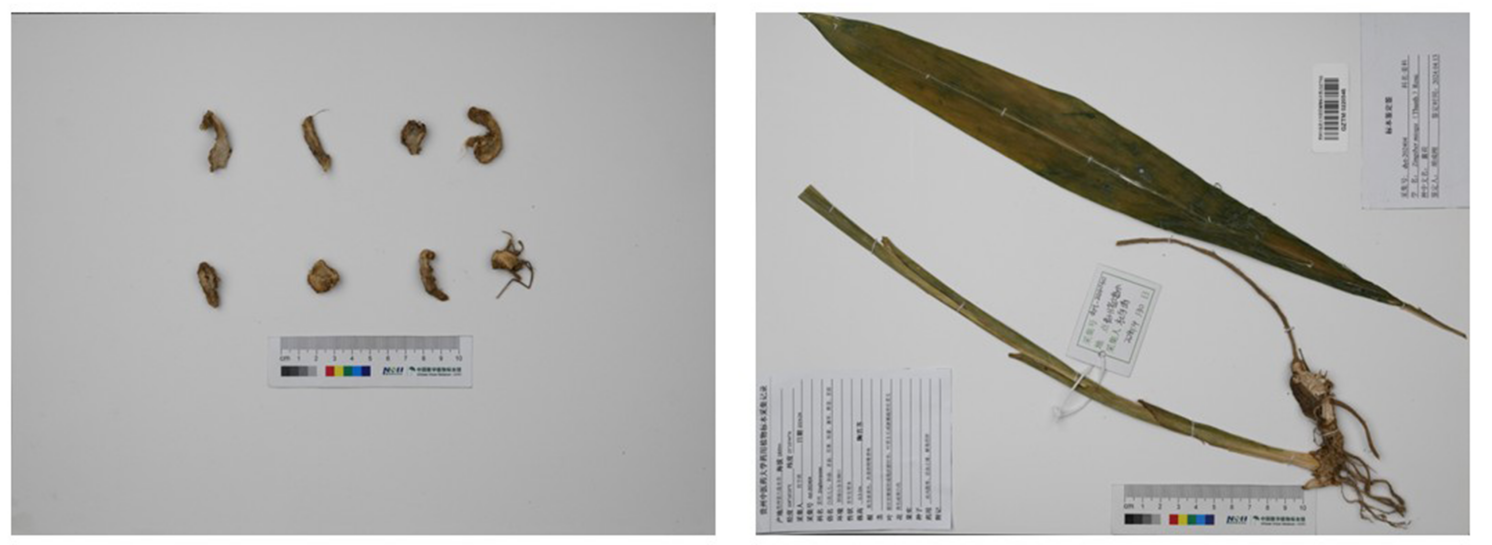


Figure 1. Specimen of*Zingiber mioga* (Thunb.) Rosc.(Left), Root (Right)


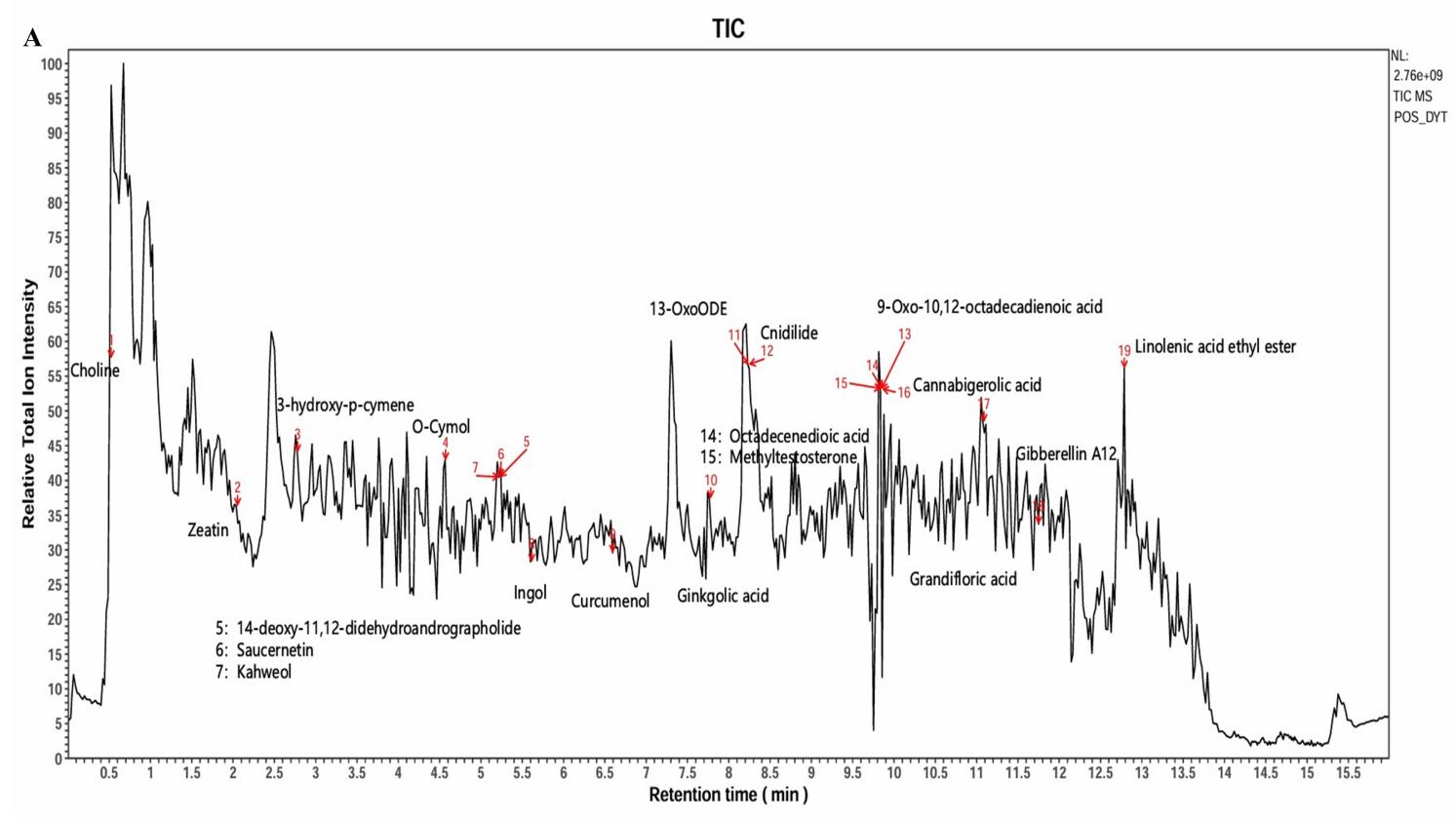

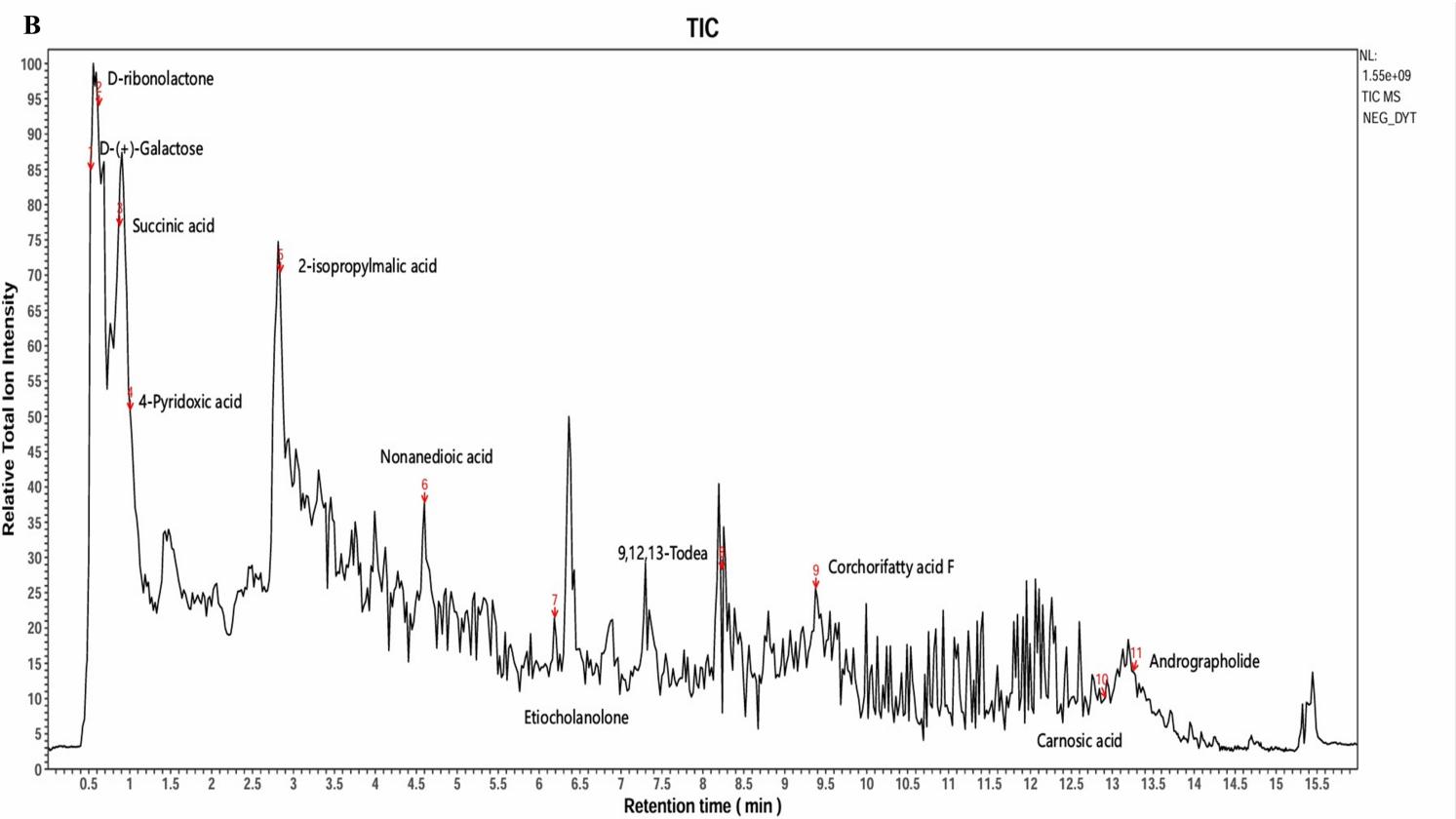



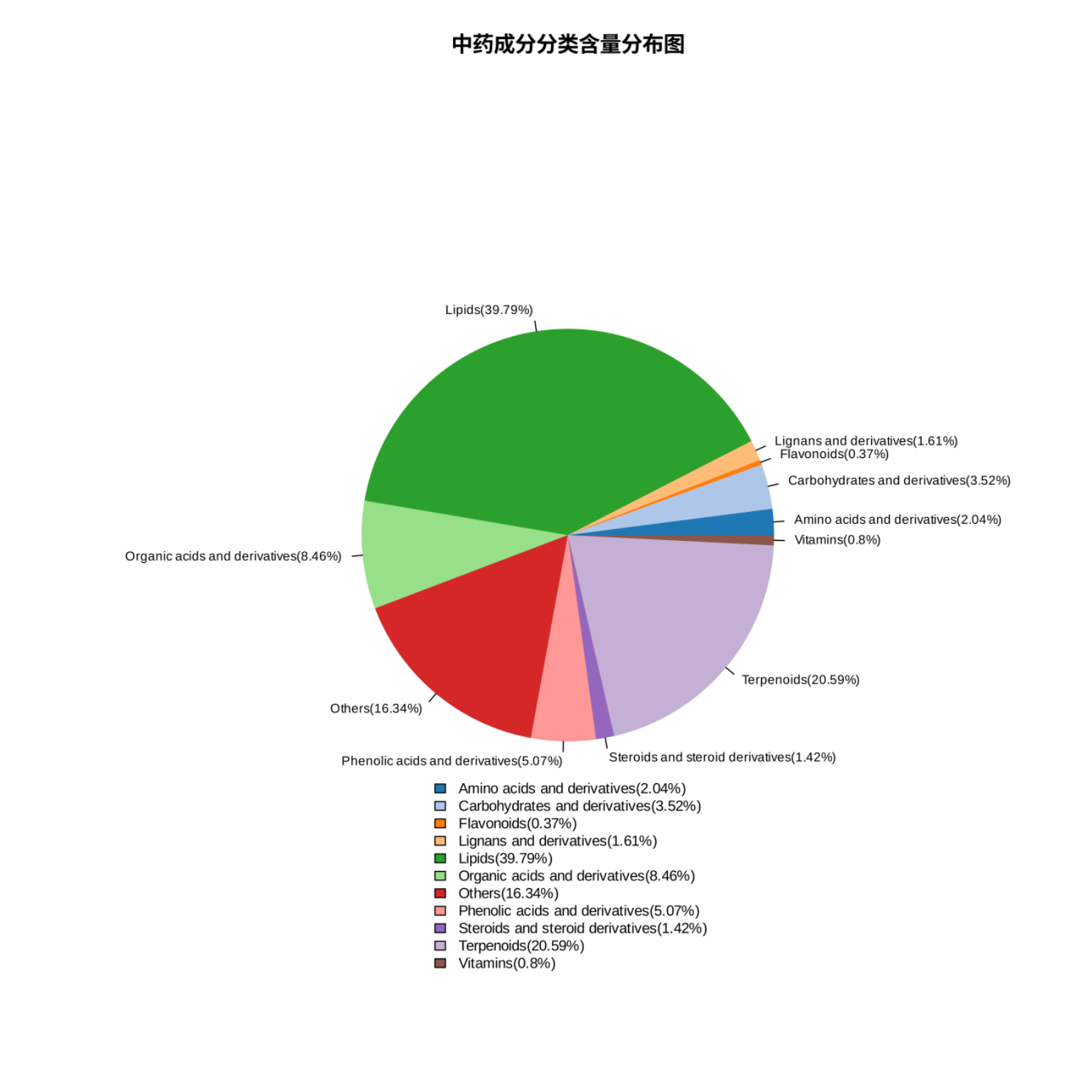
Figure 2. Identification results of the aqueous extract of RH. (A) Content distribution by component class of RH ingredients; (B) Quantity distribution by component class of RH ingredients.


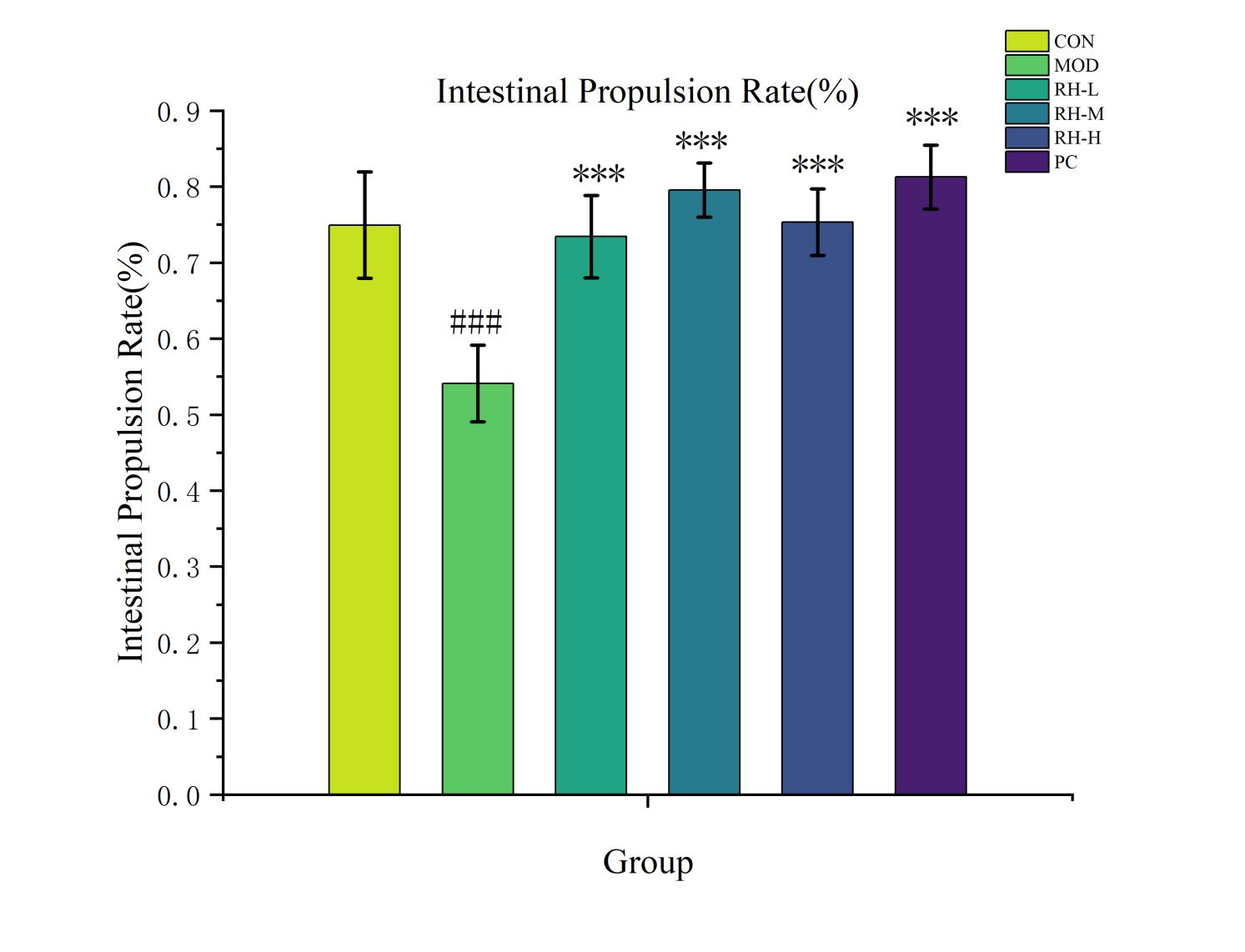


Figure 3.Figure of Intestinal Propulsion Rate.Carbon powder propulsion rate of each group(n=6). Compared with the normal group, ###P < 0.001; compared with the model group, ***P < 0.001.


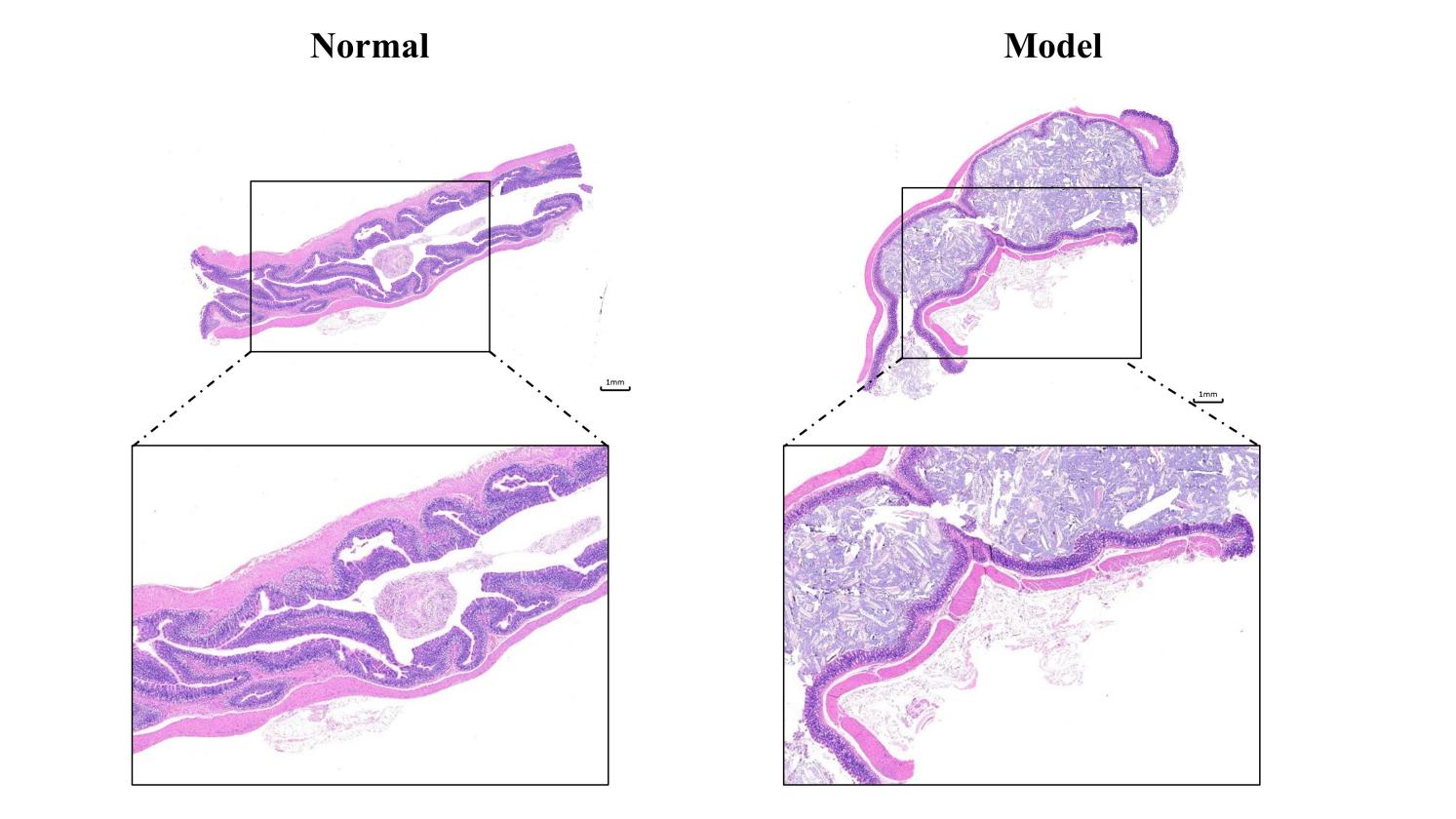


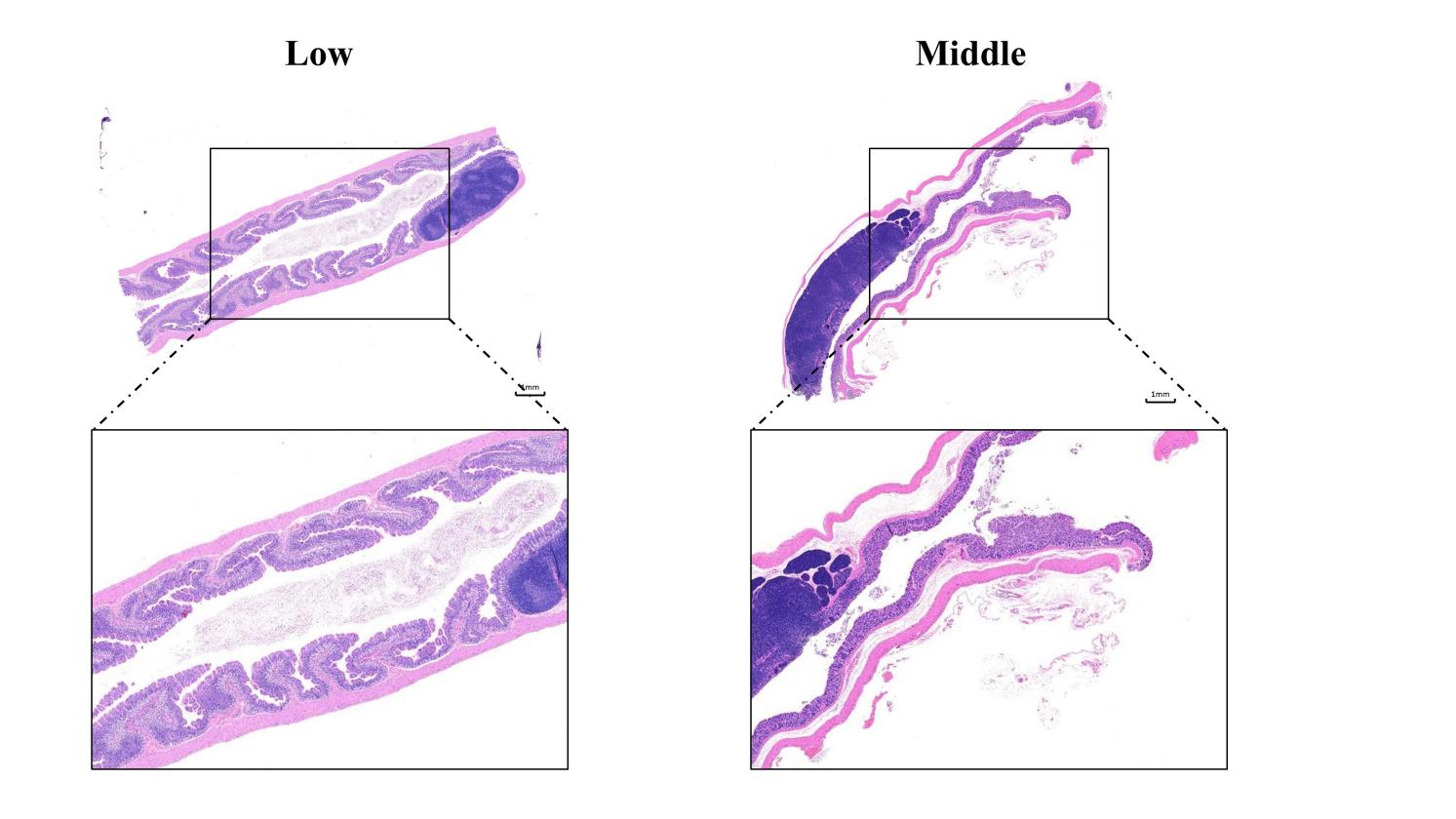

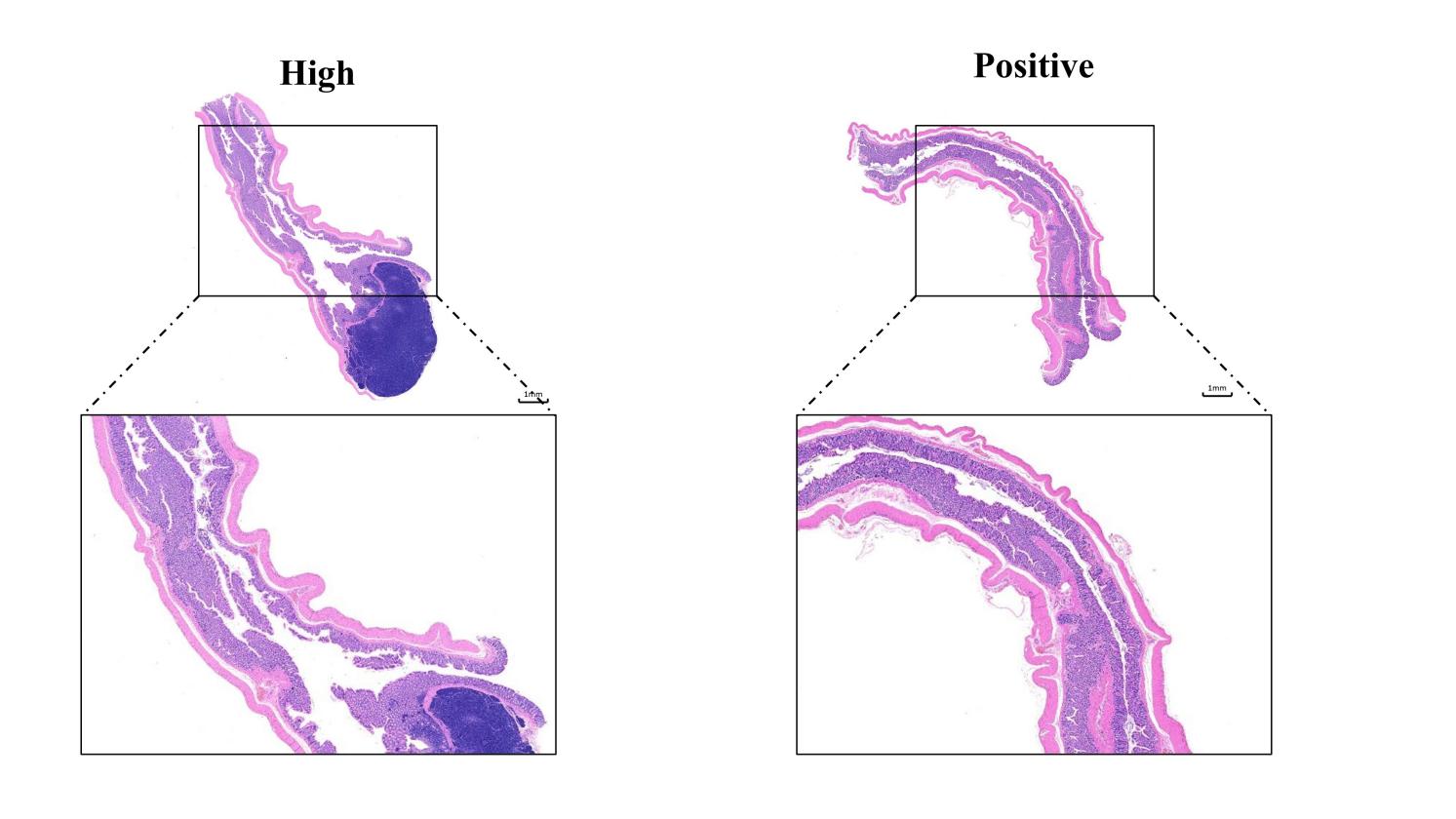

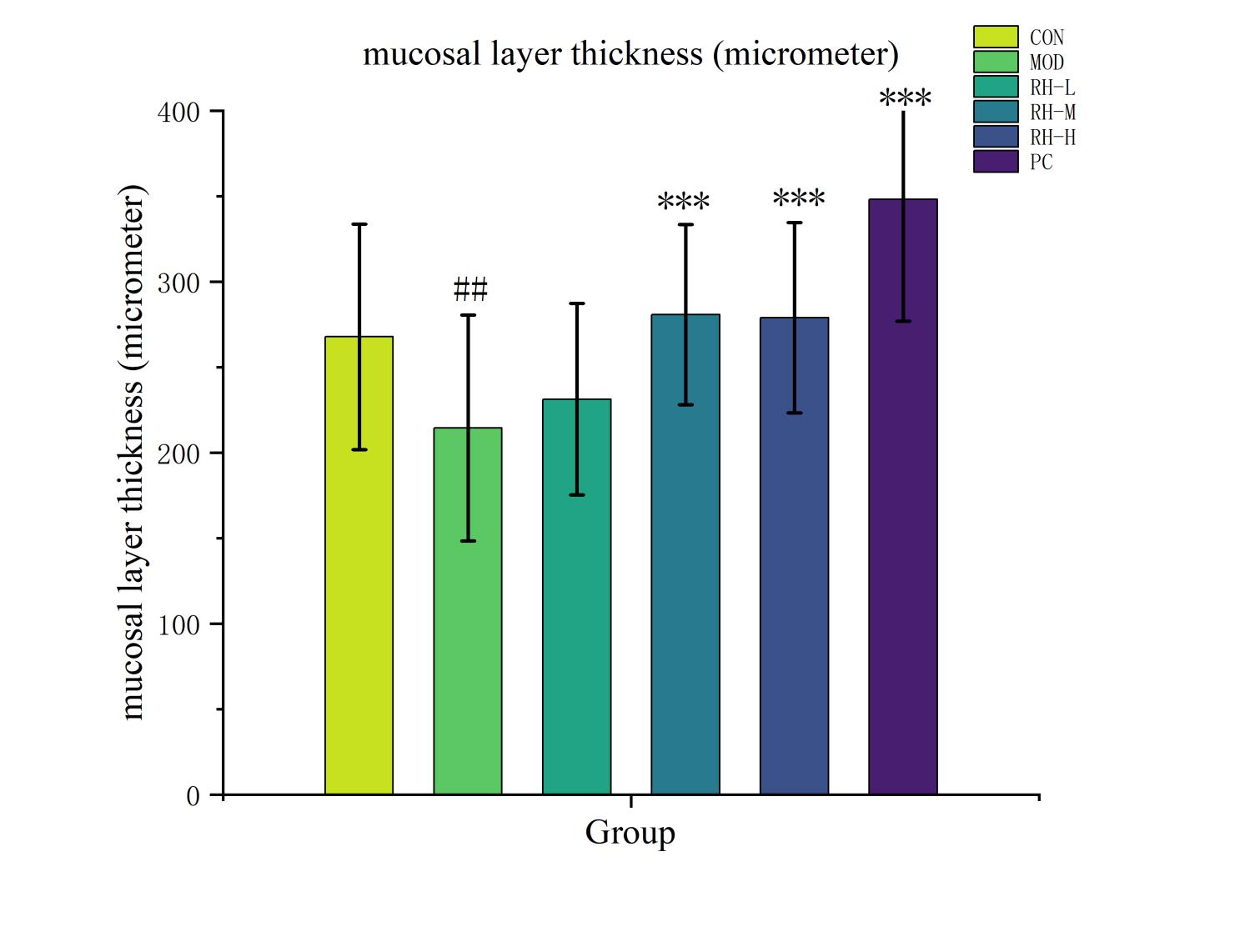

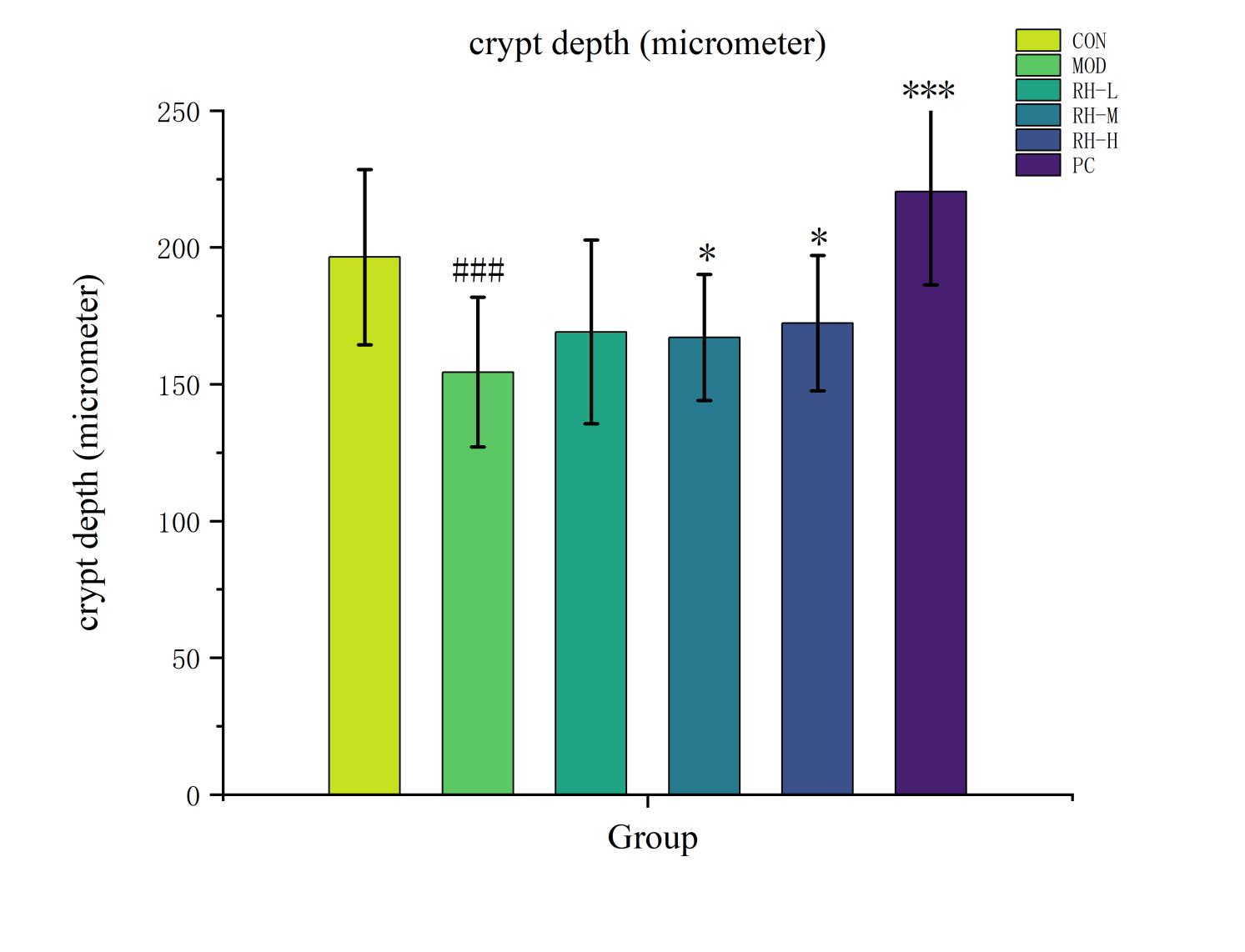


Figure 4.Figure of HE Staining Results of Rat Colon.


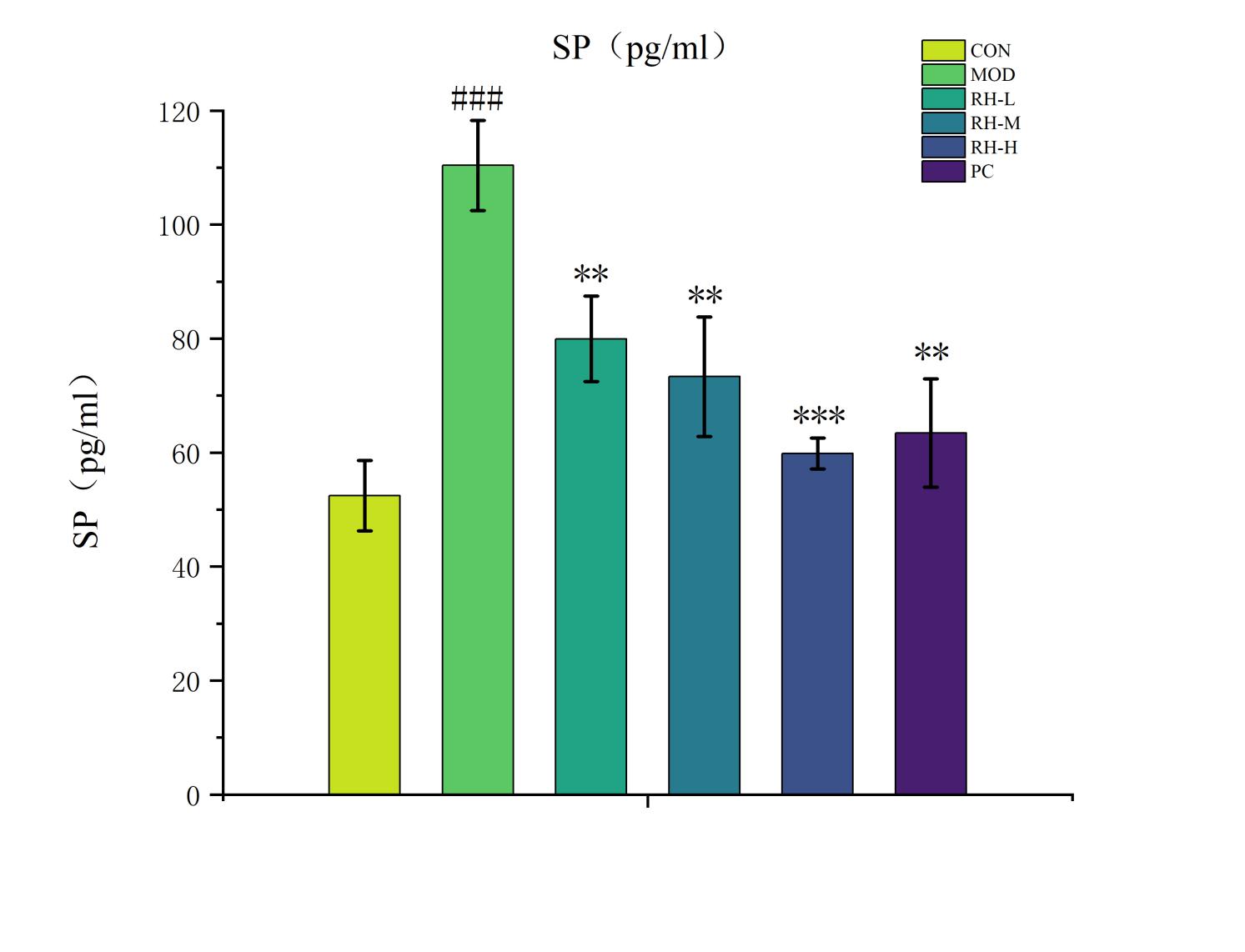

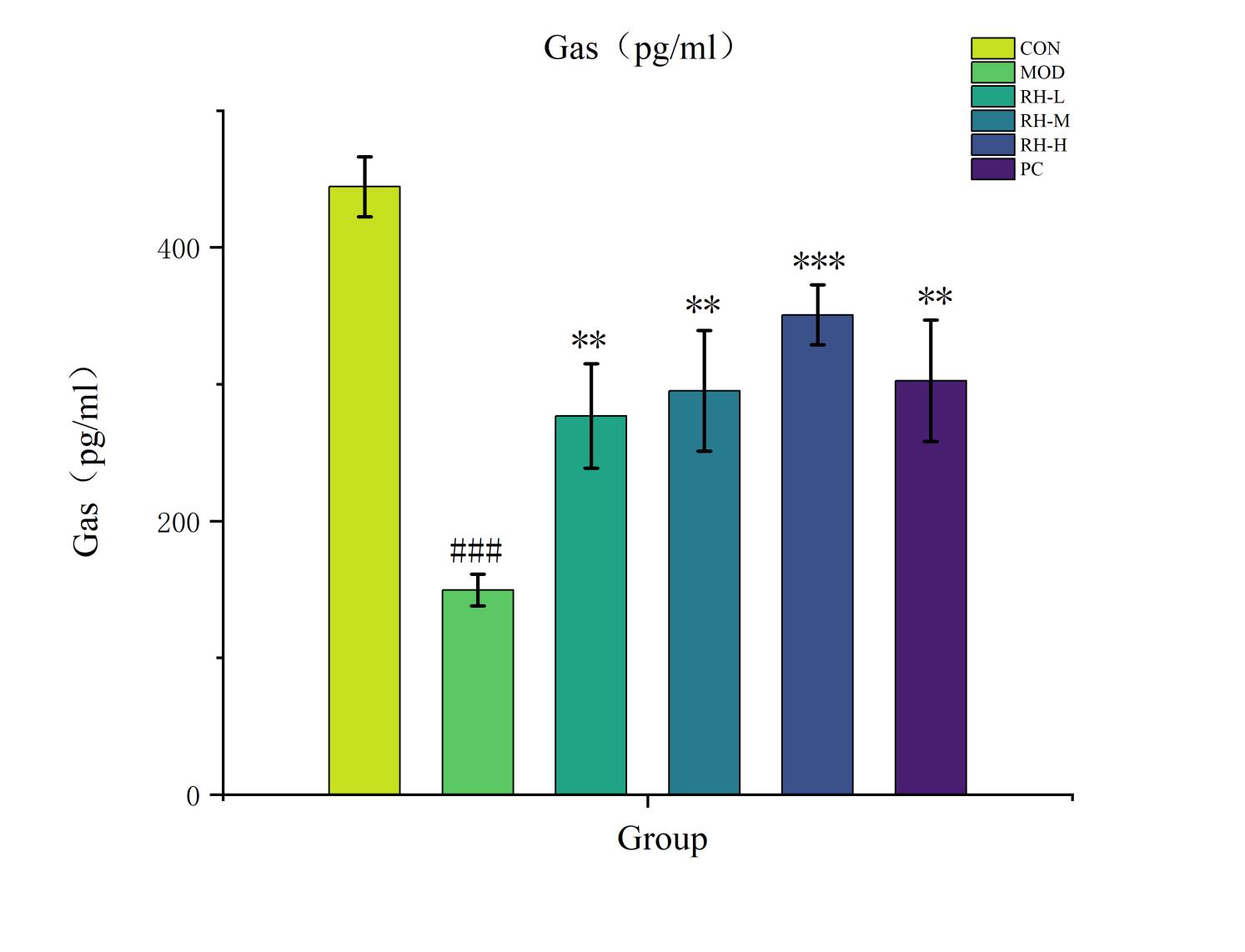
Figure 5. Serum SP, MTL, and GAS levels in each group.


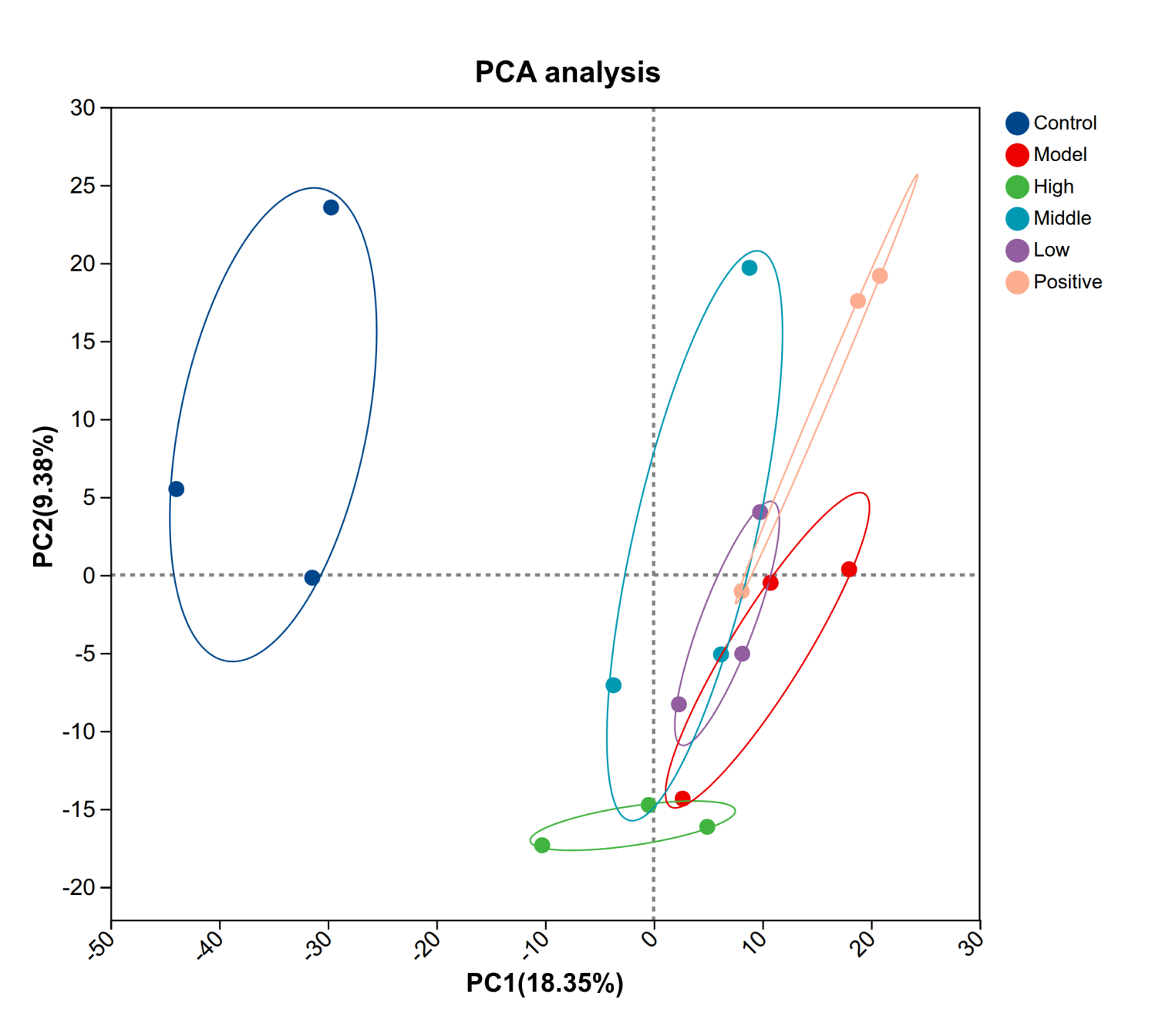

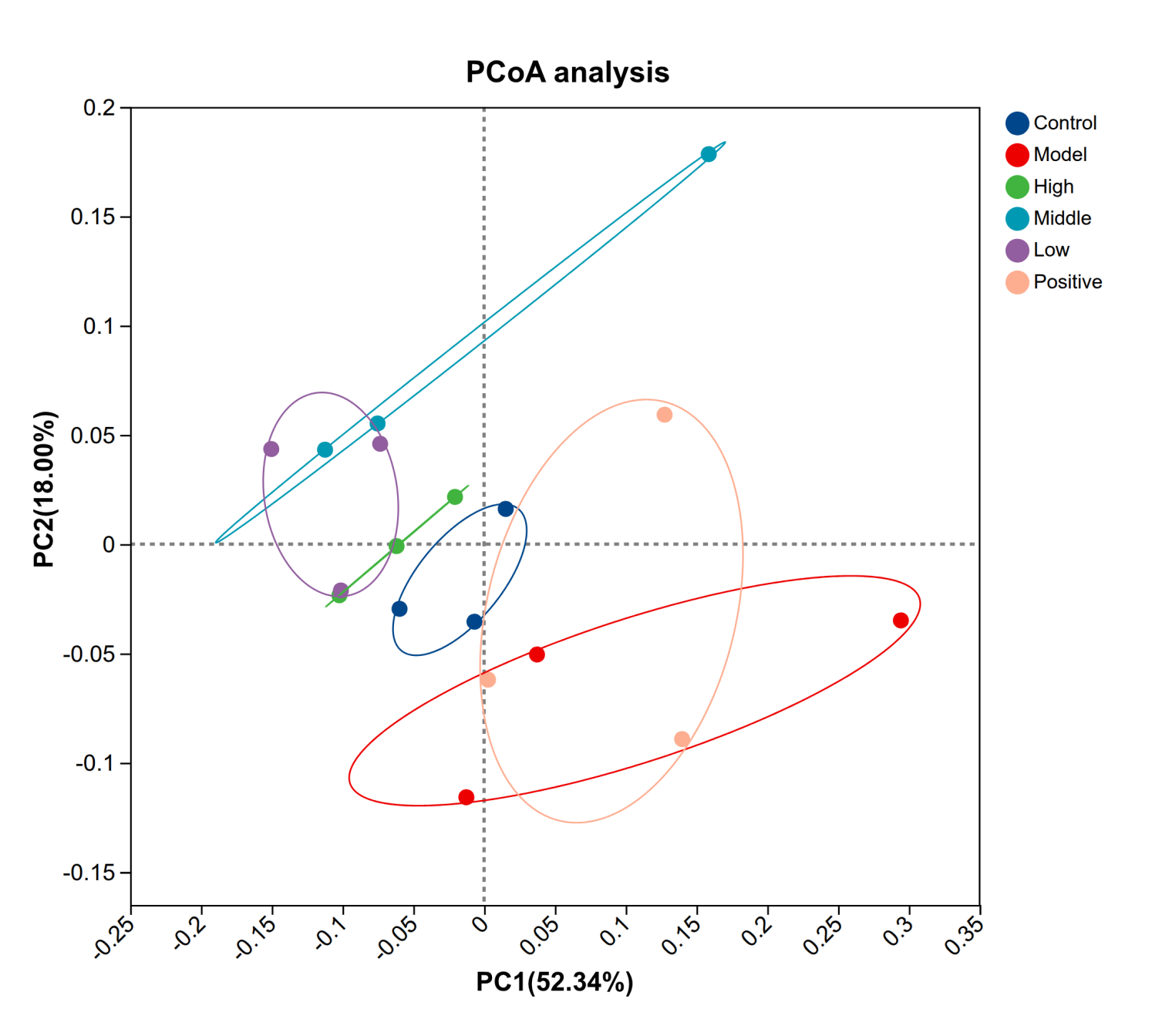

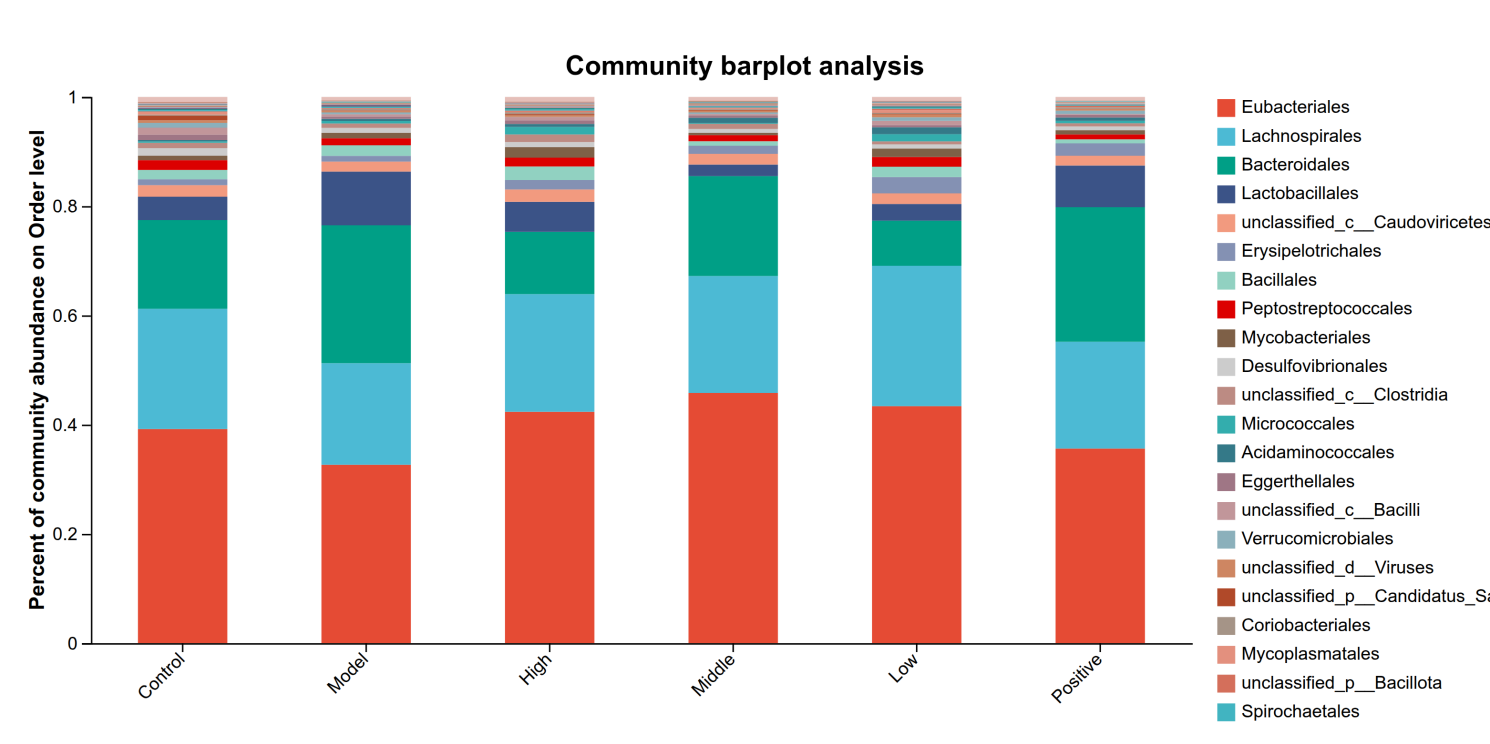

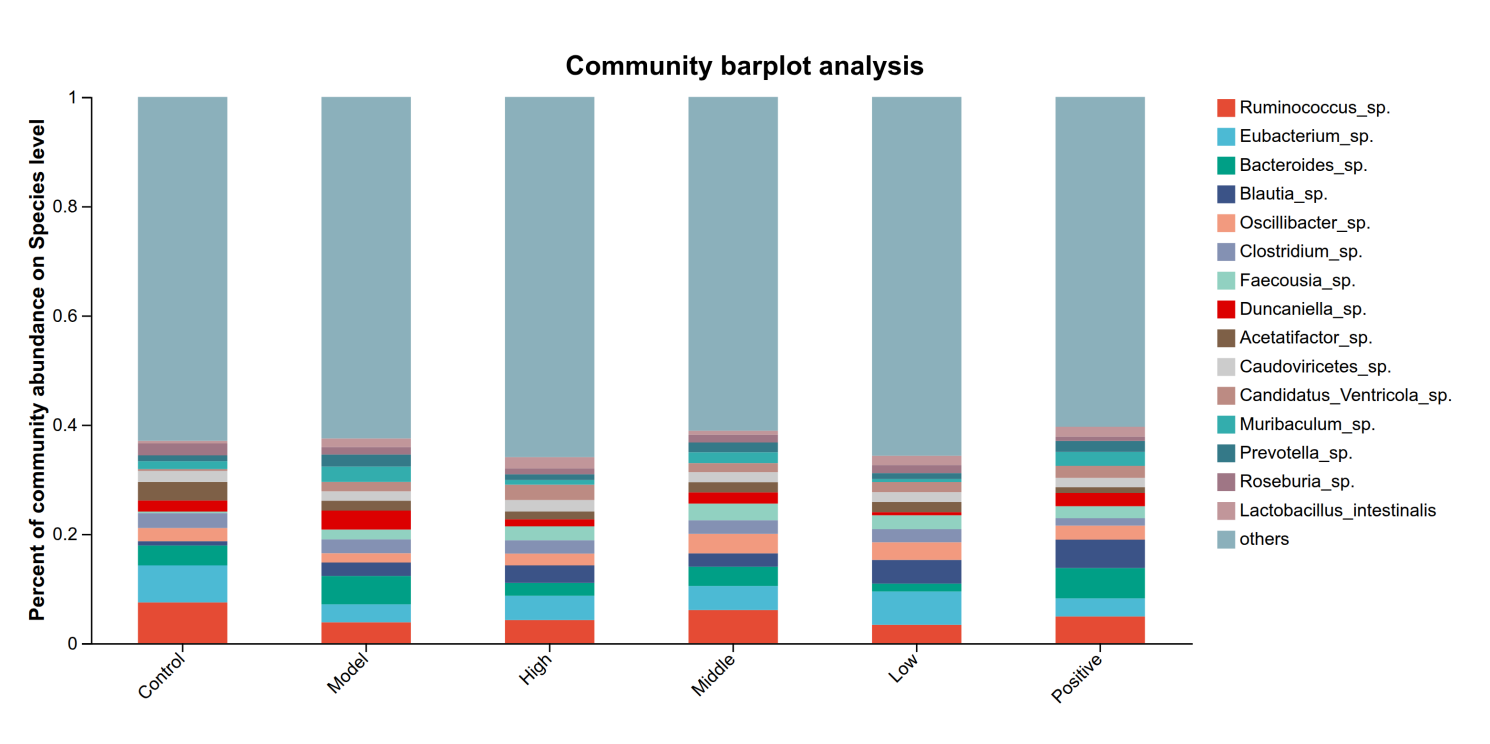

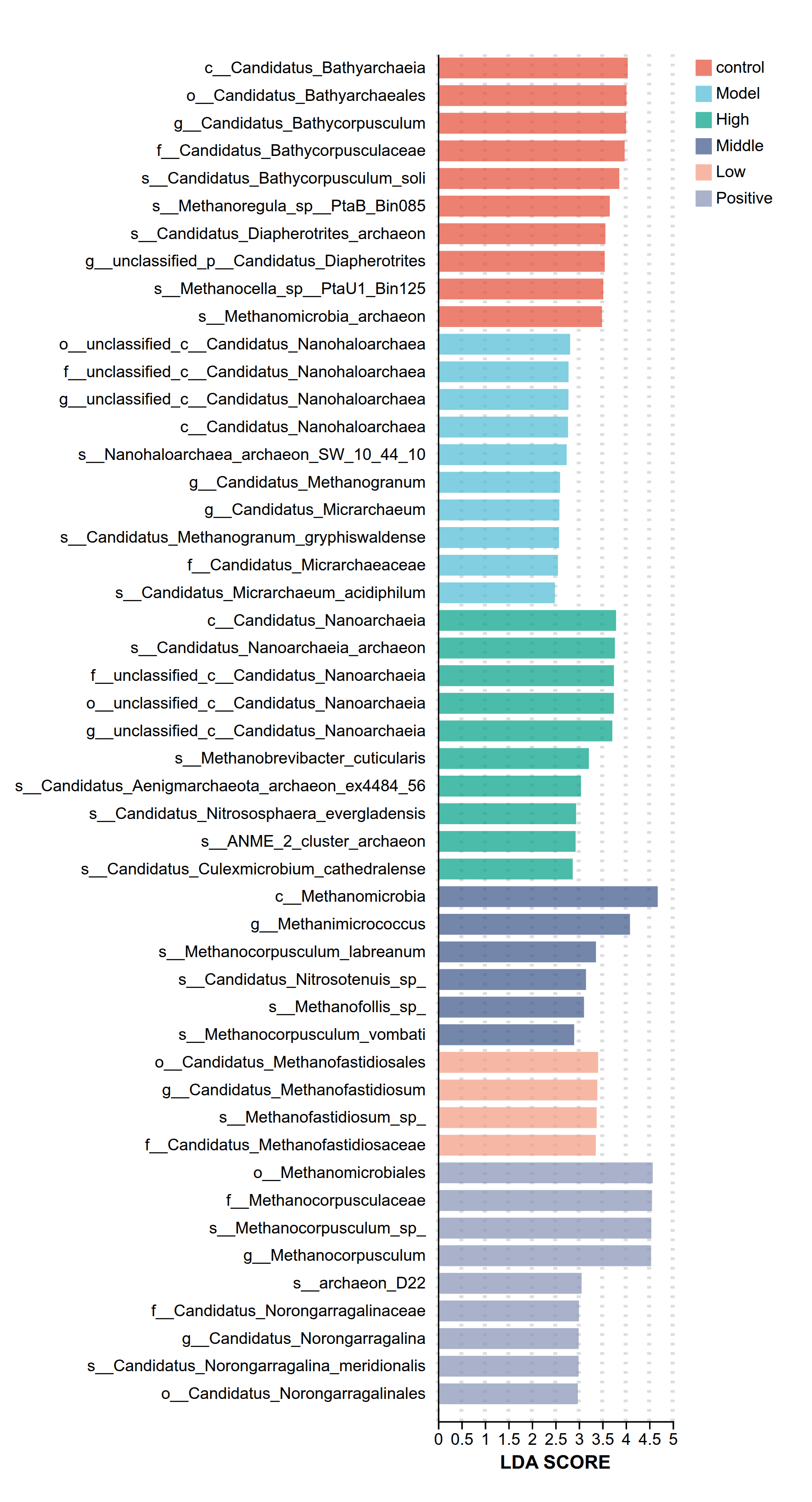

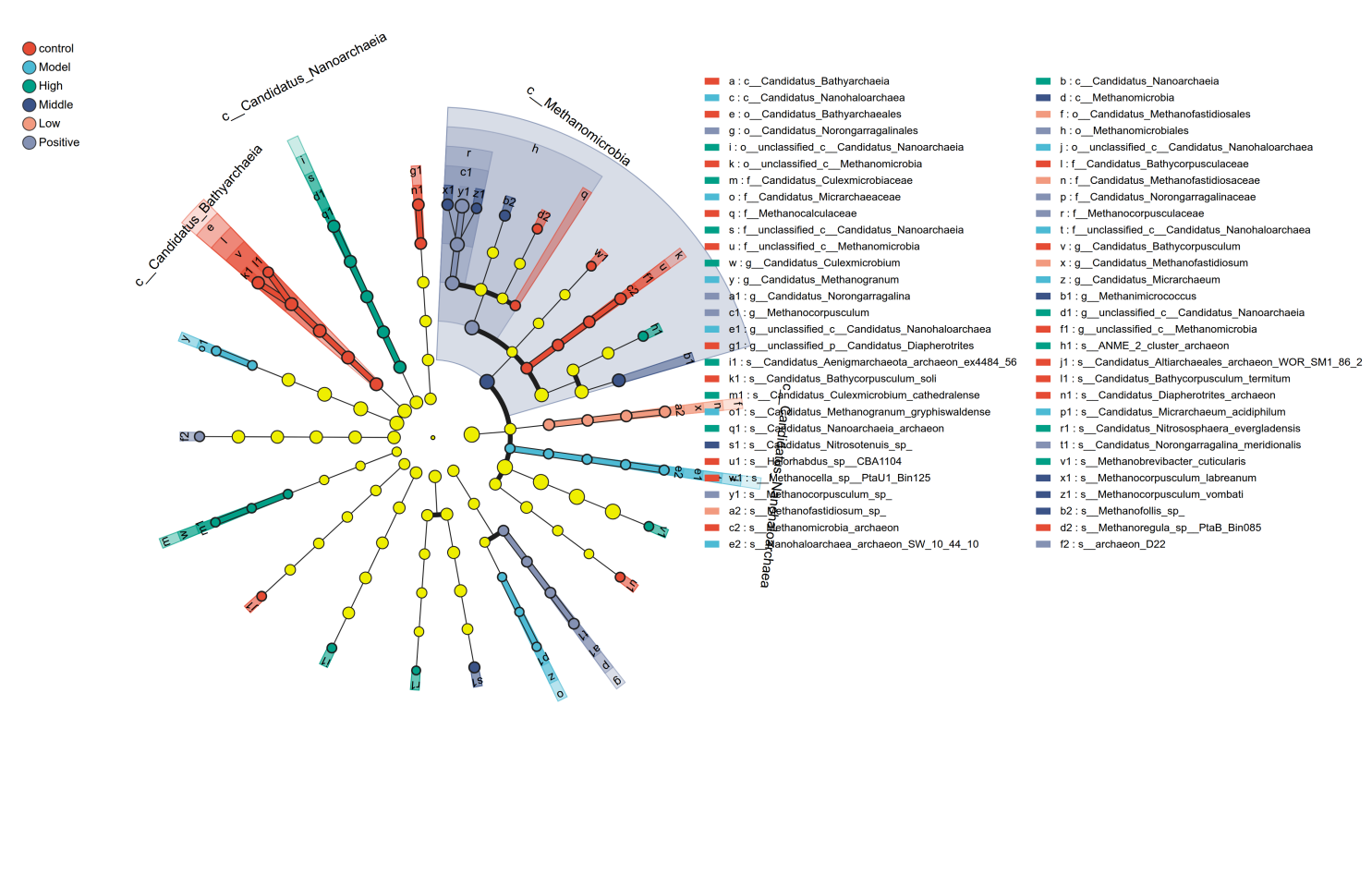

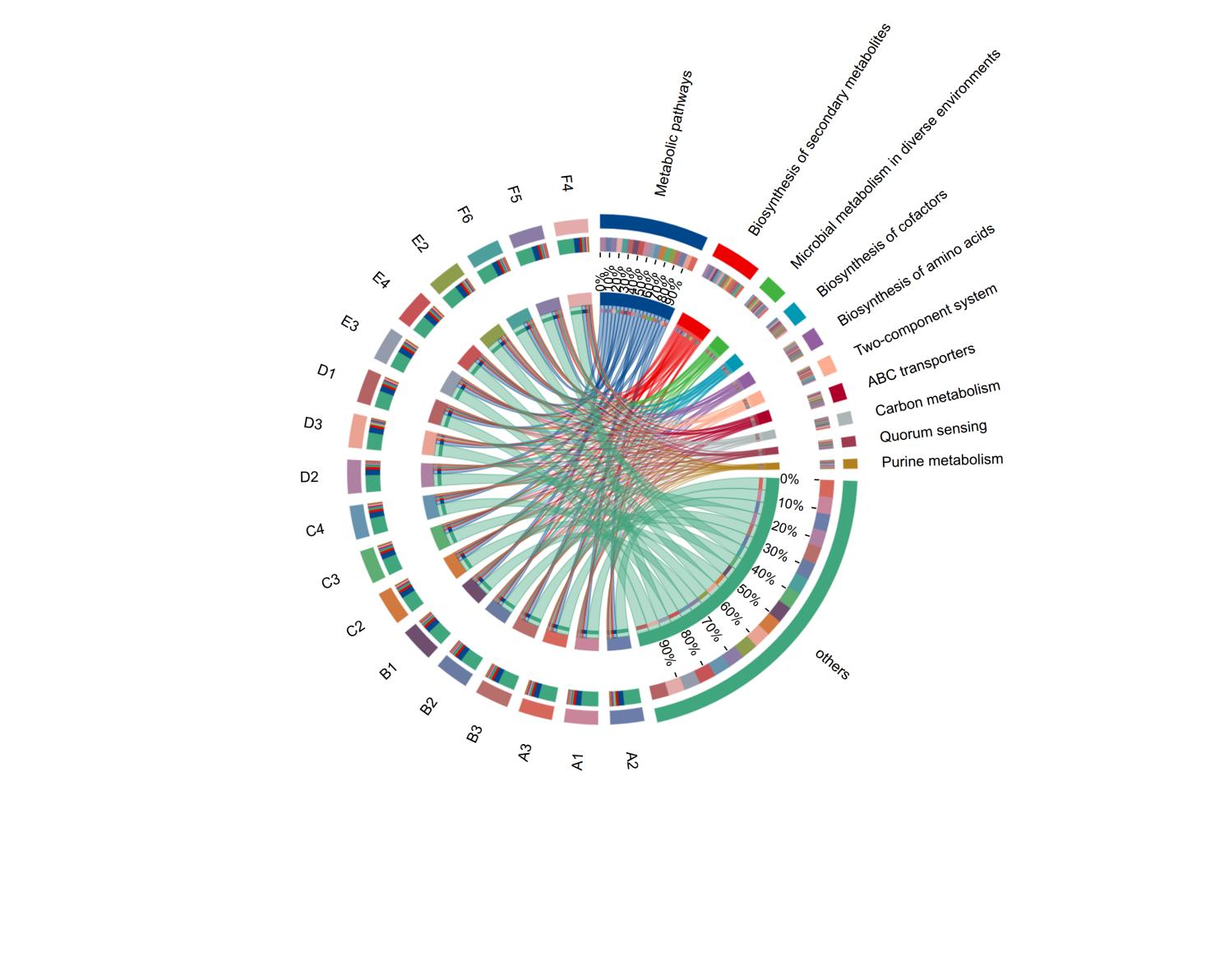
Figure 6. Metagenomic analysis of intestinal microbiota in STC rats following RH intervention. **
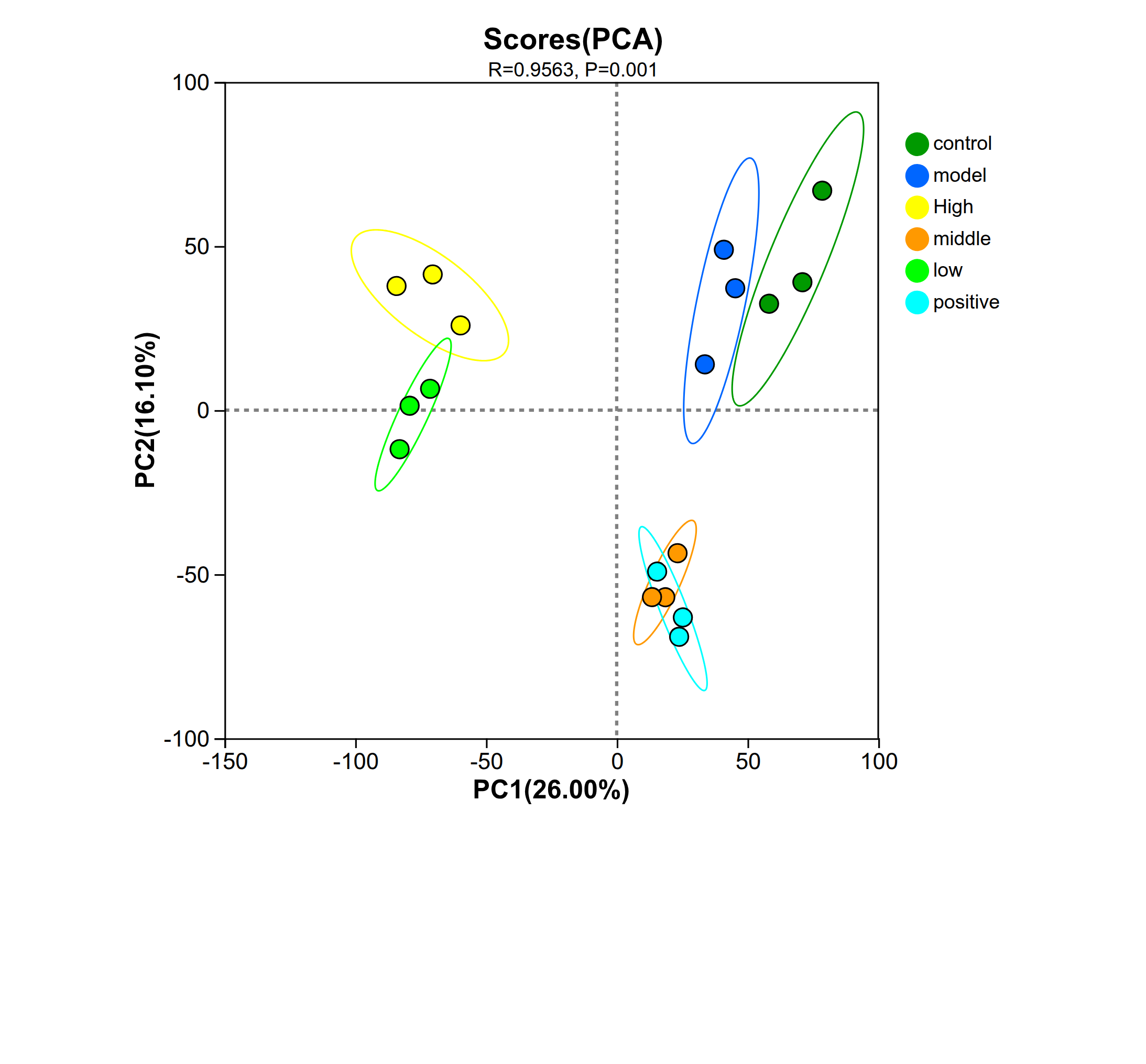

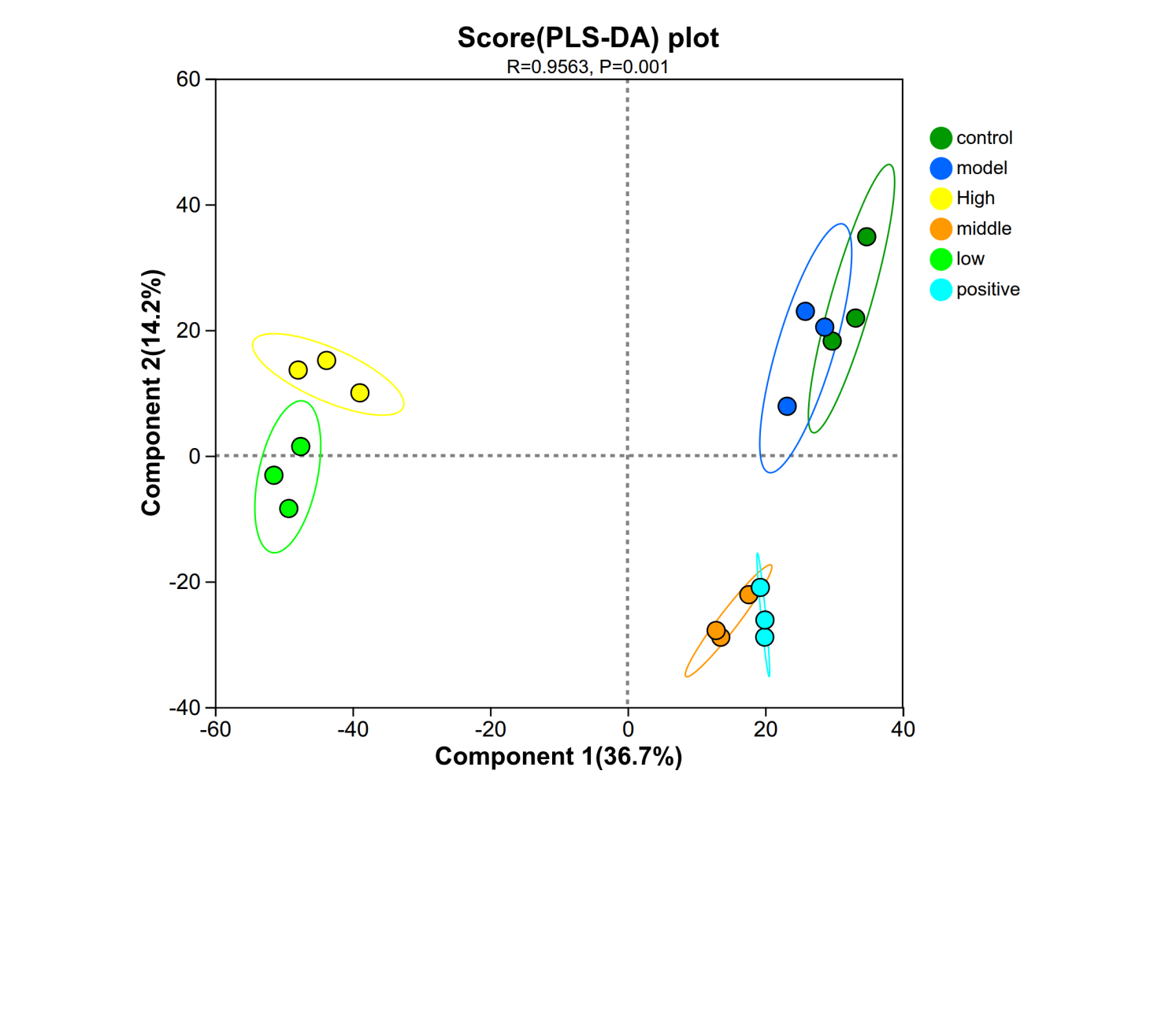

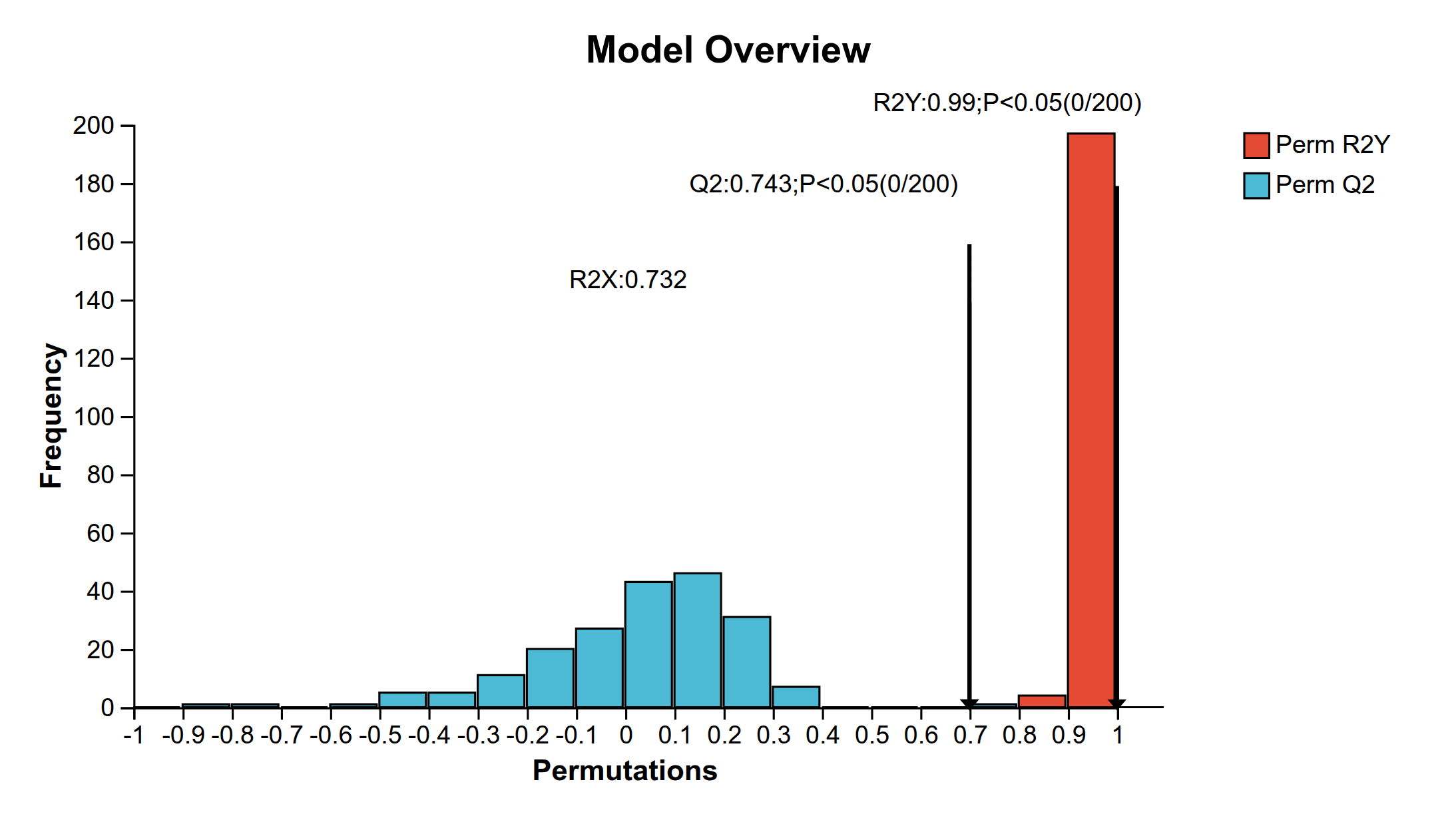
**Figure 7. Multivariate statistical analysis of serum metabolomics in STC rats following RH intervention.**
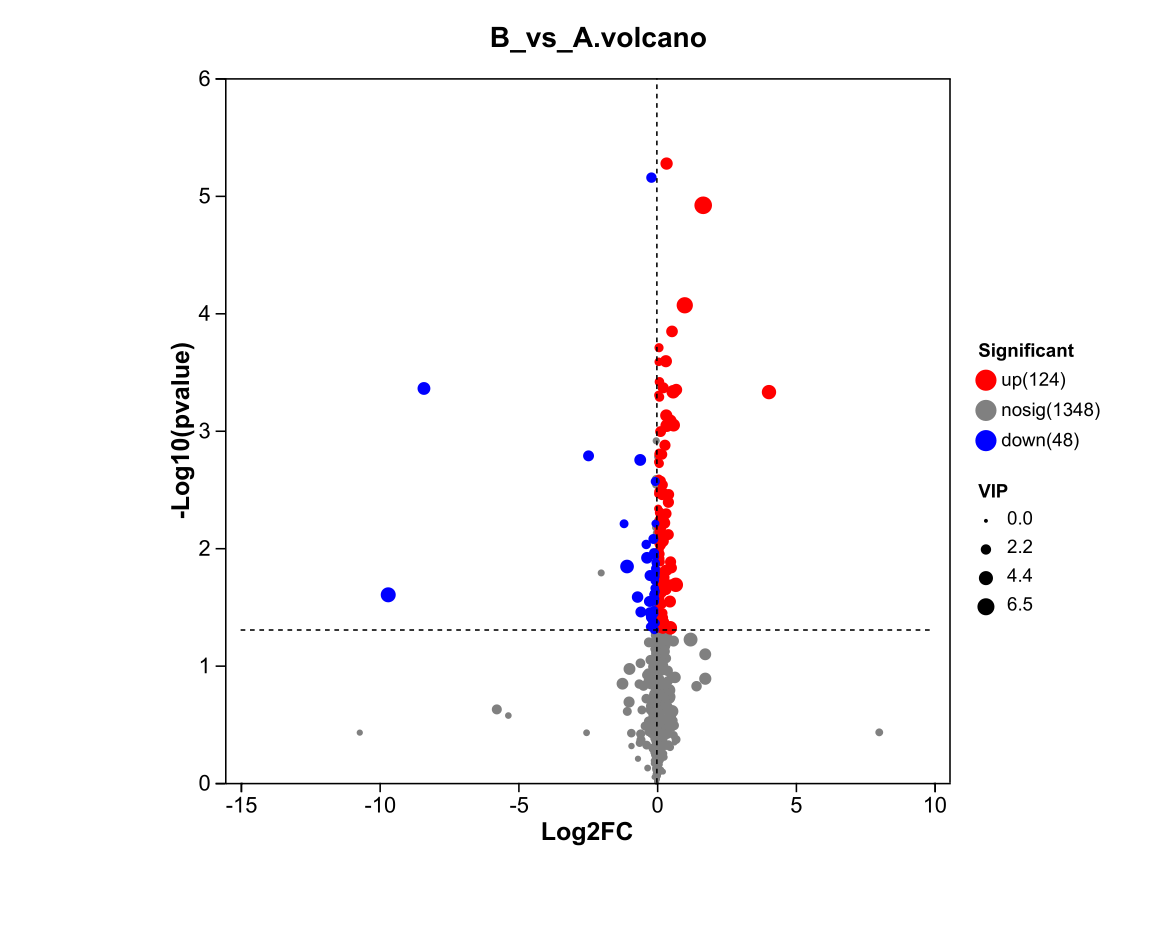

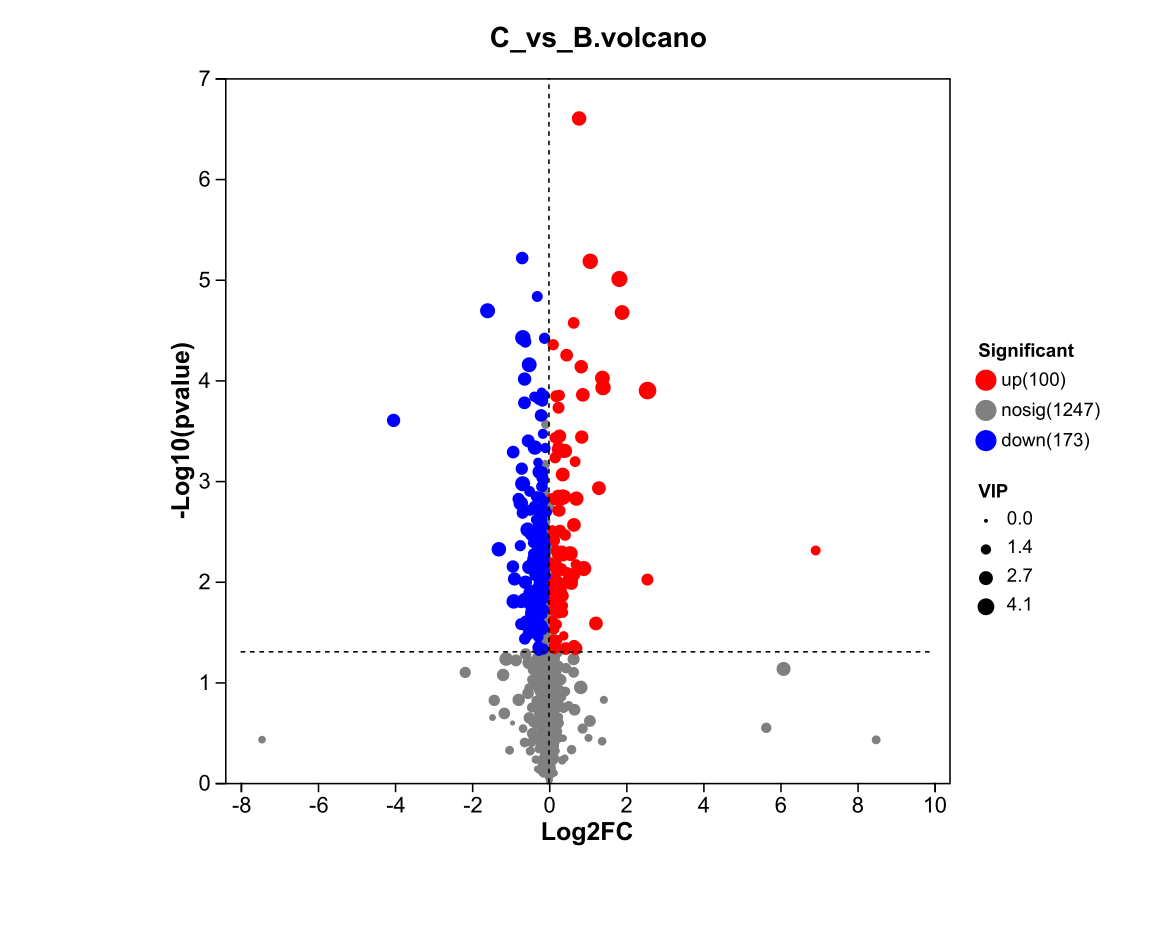

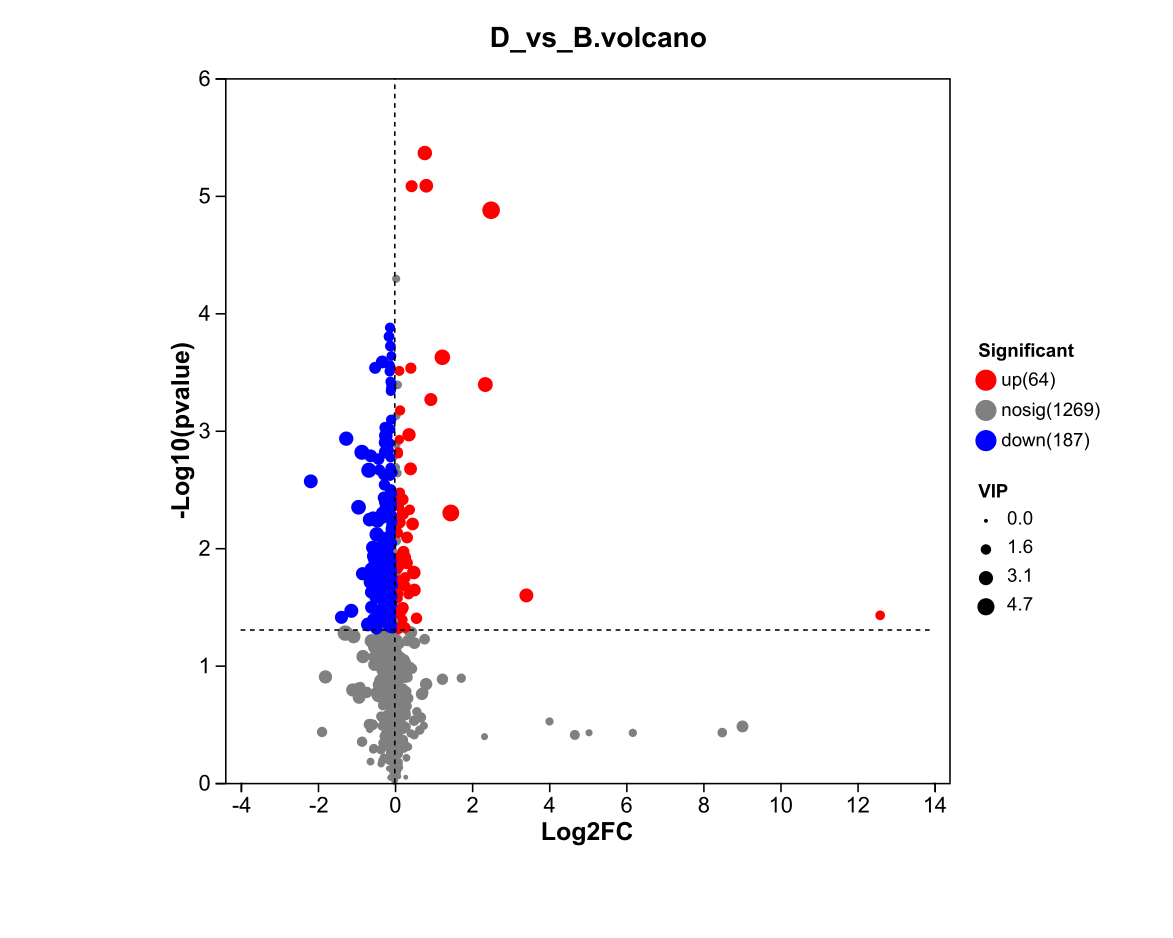

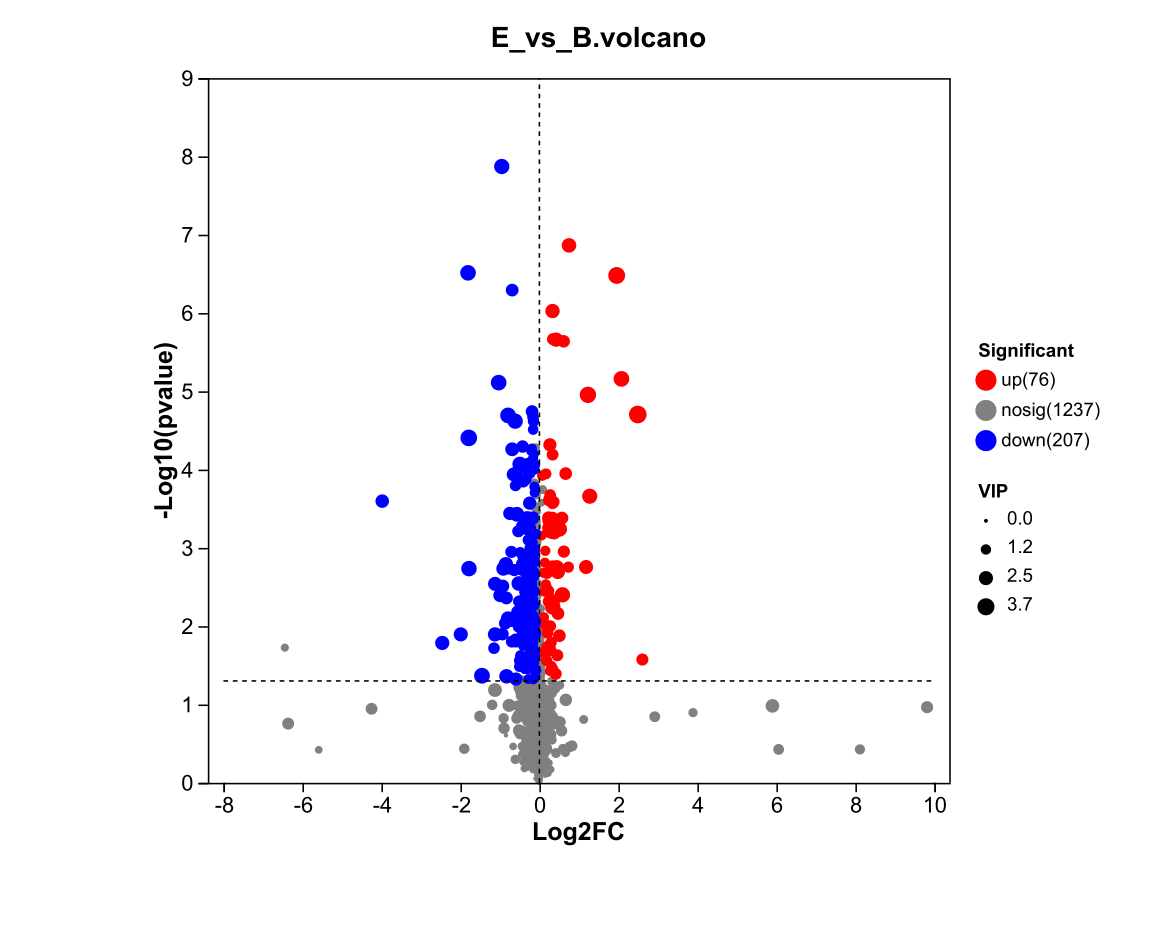

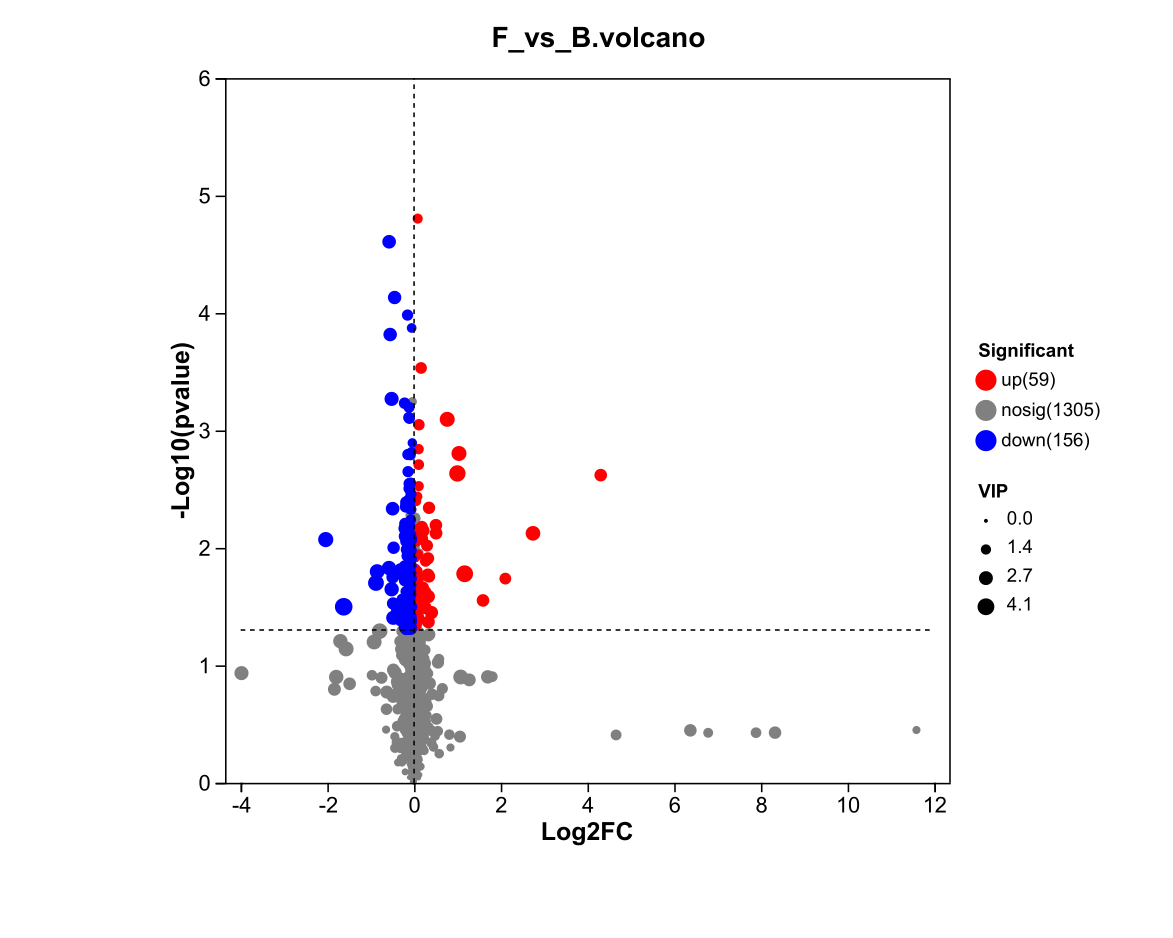
**Figure 8. Statistical analysis of serum differential metabolites in STC rats following RH intervention.


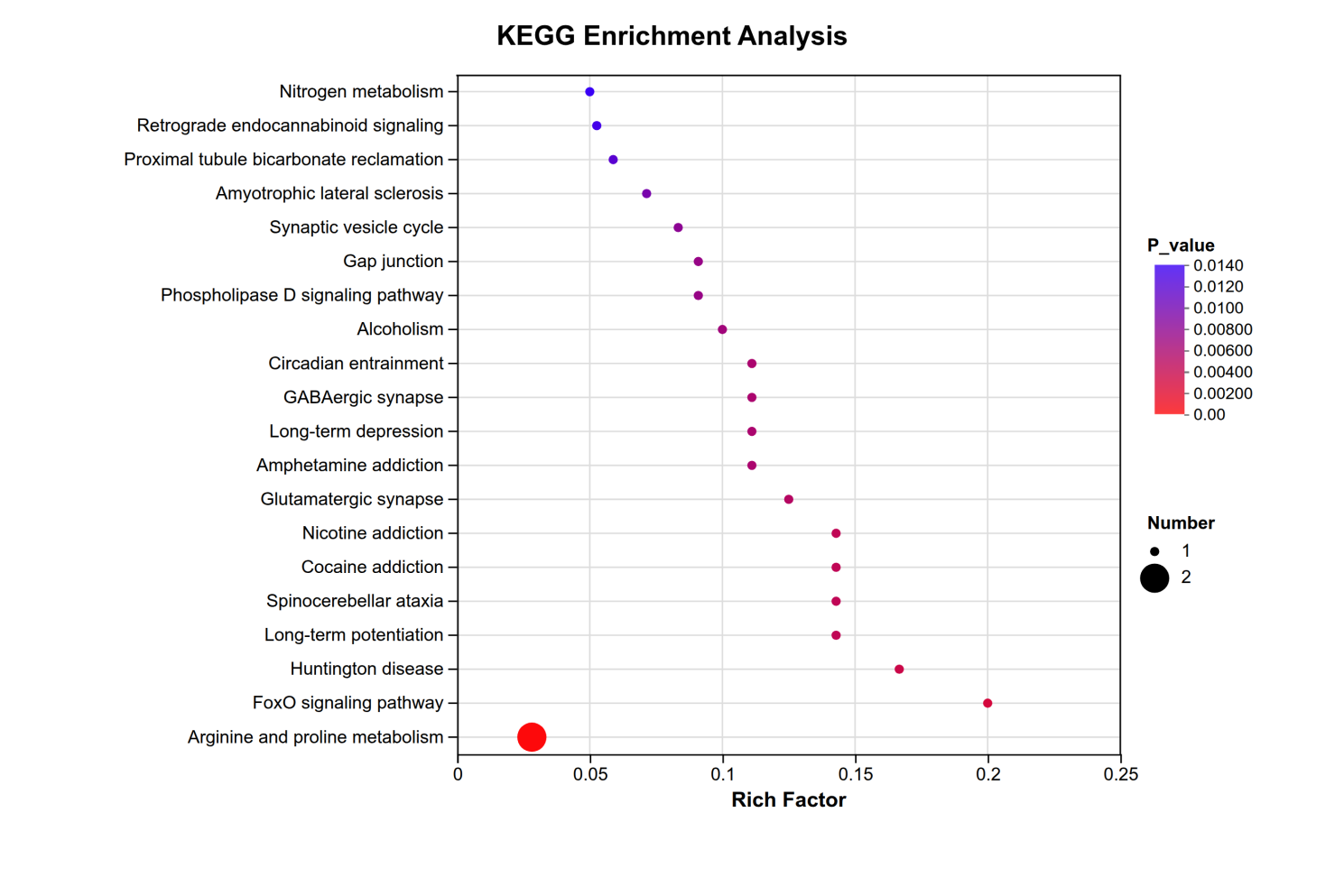

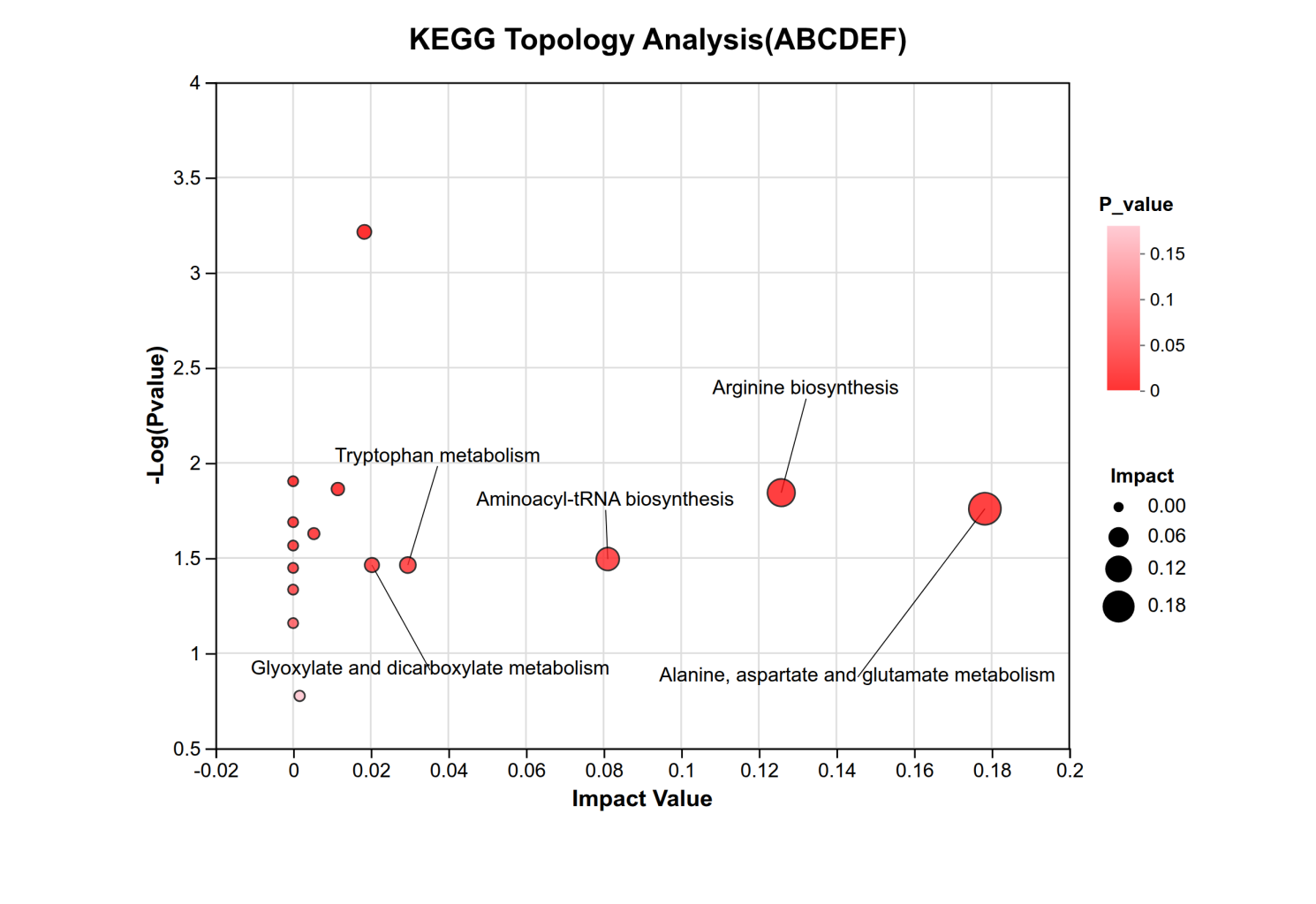

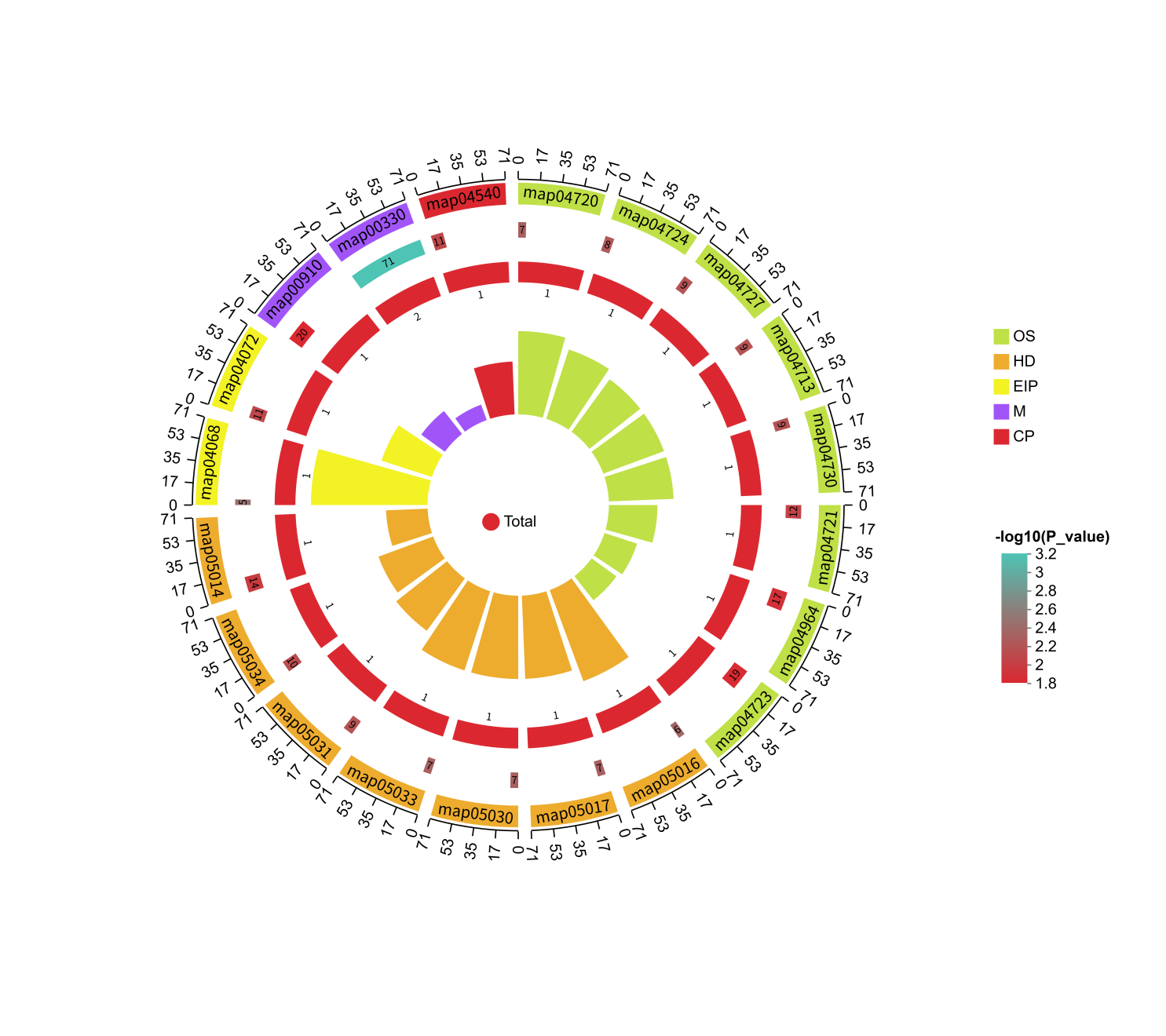

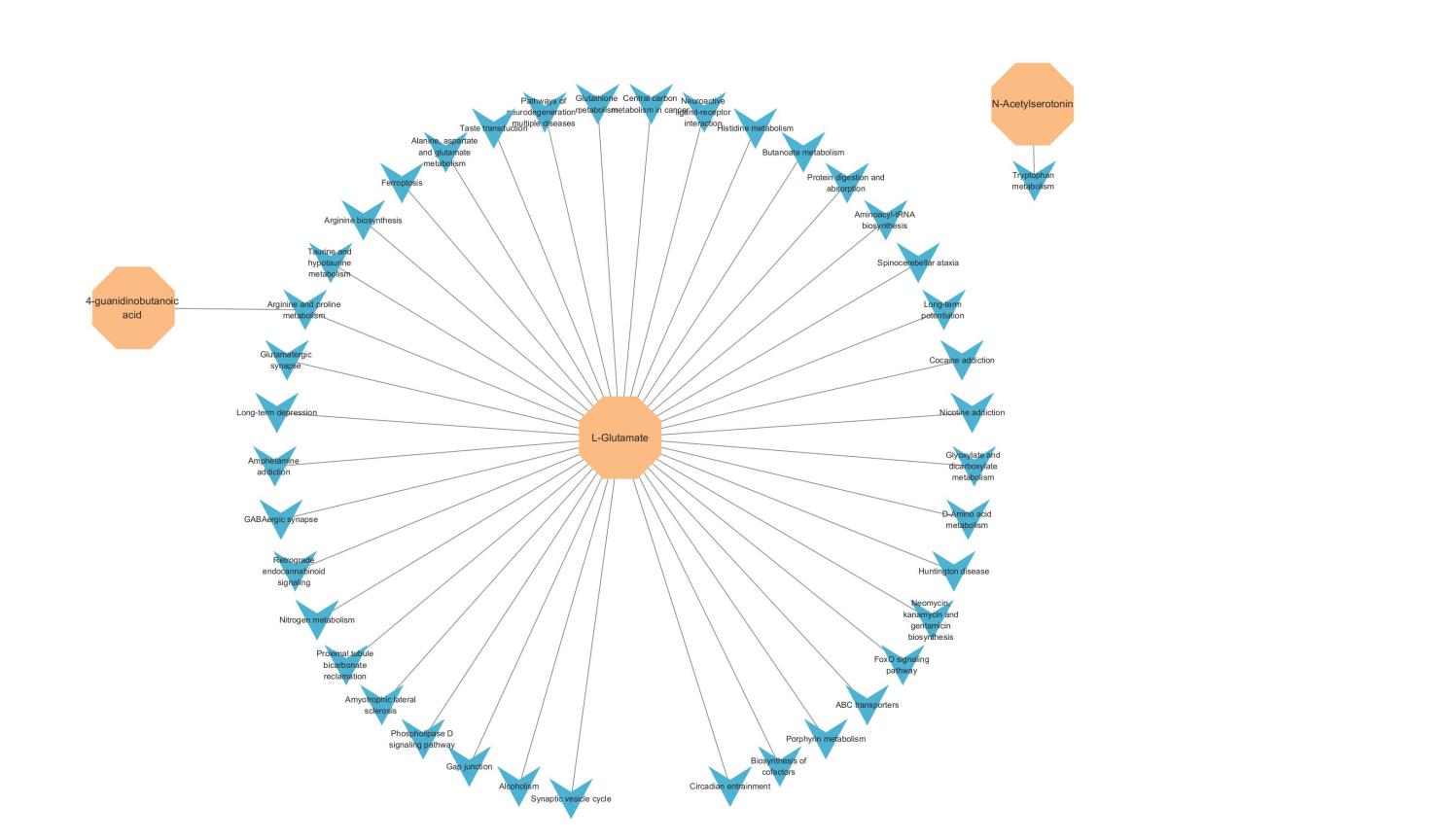
Figure 9. KEGG pathway enrichment and correlation network analysis of serum differential metabolites in STC rats following RH intervention.


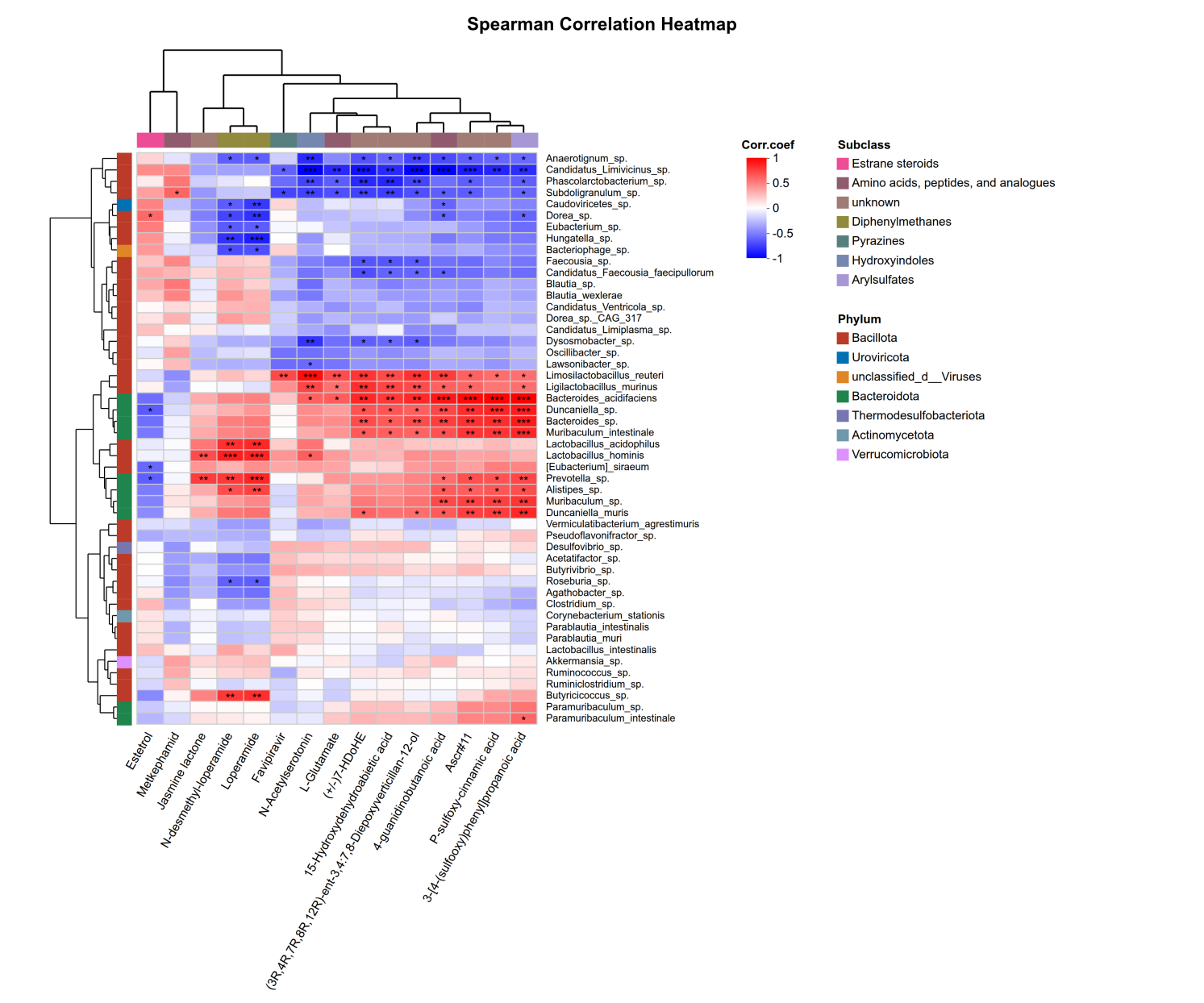

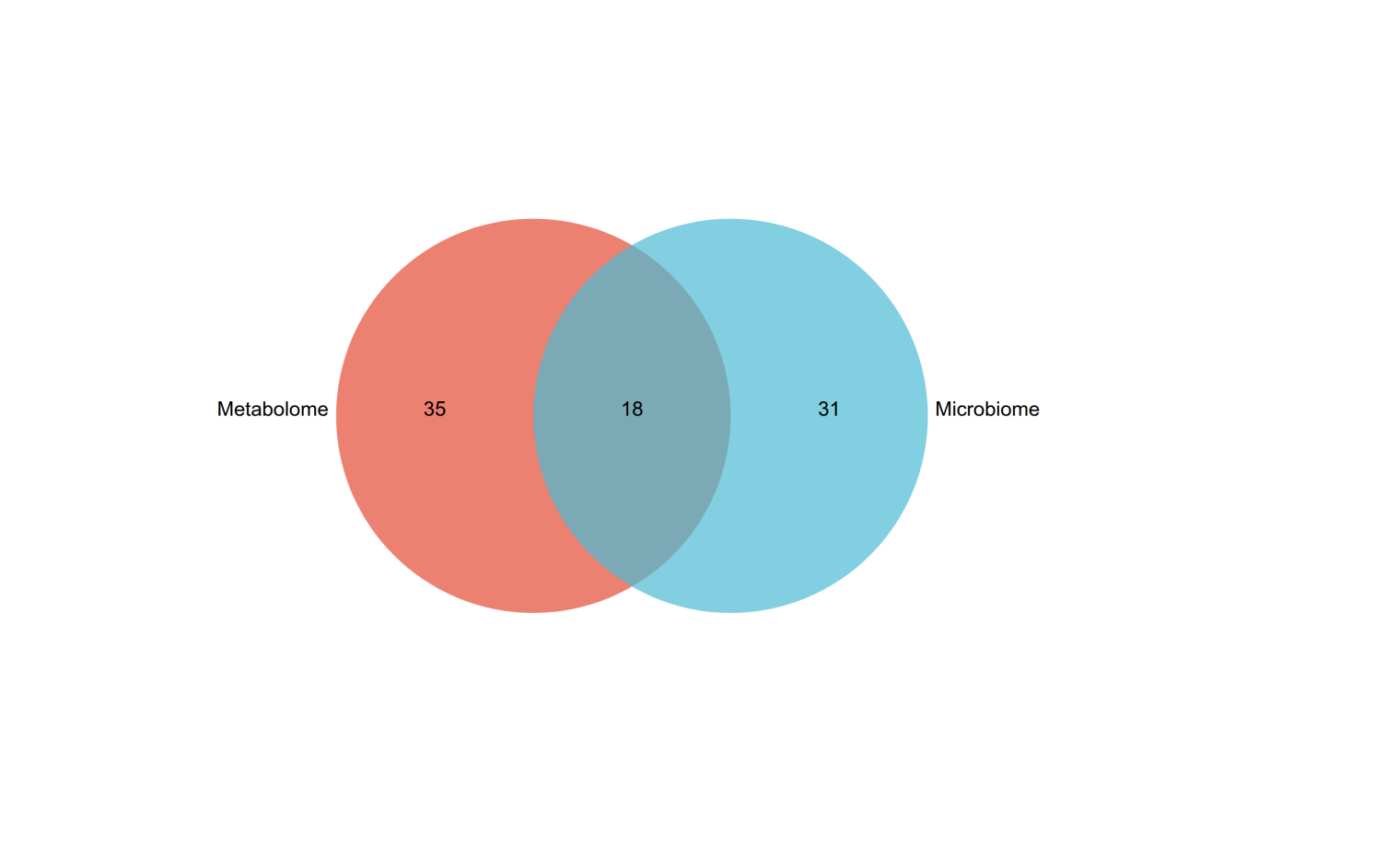

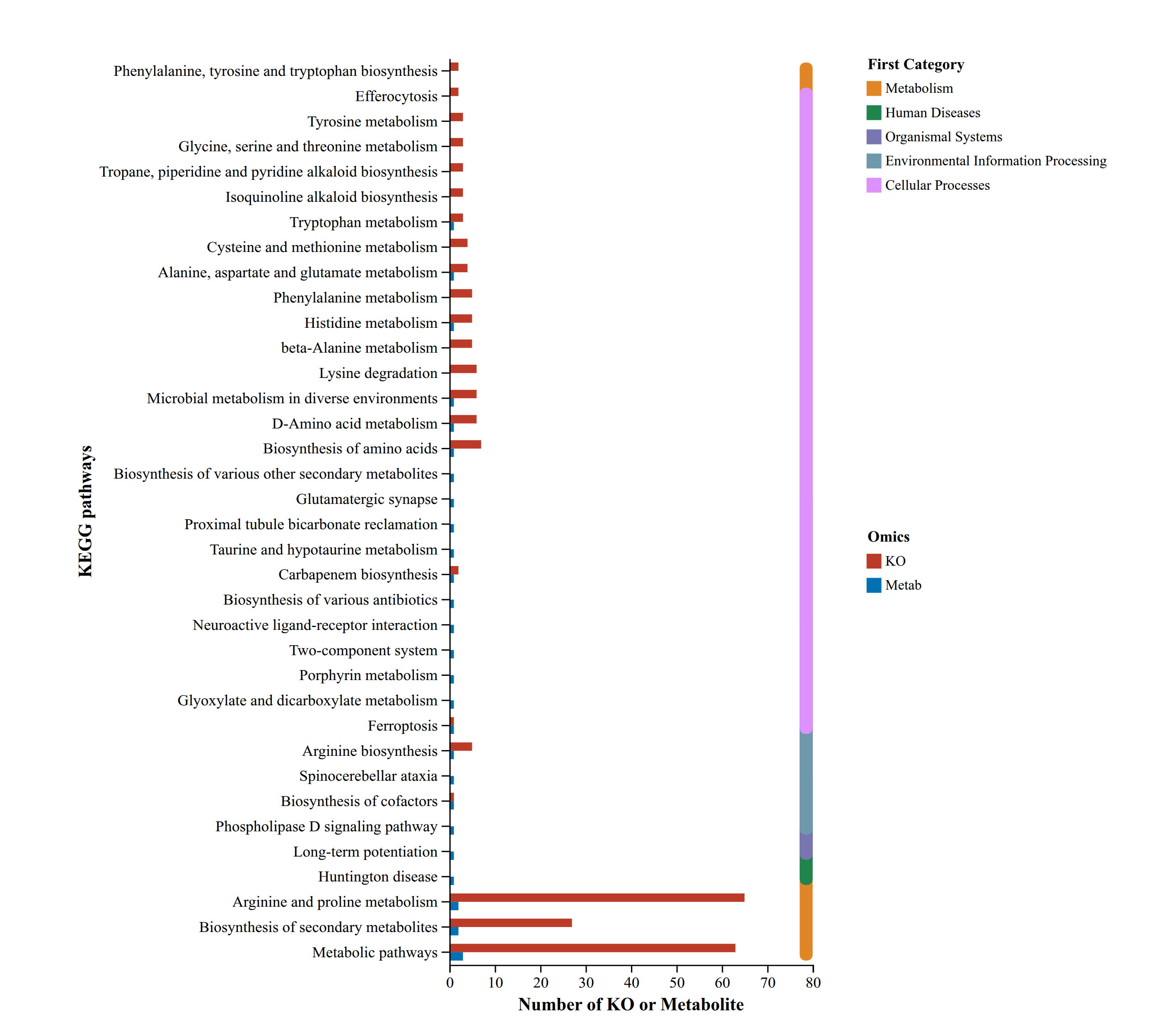
Figure 10. Multi-omics correlation analysis of microbiota-metabolite interactions in STC rats following RH intervention.

**
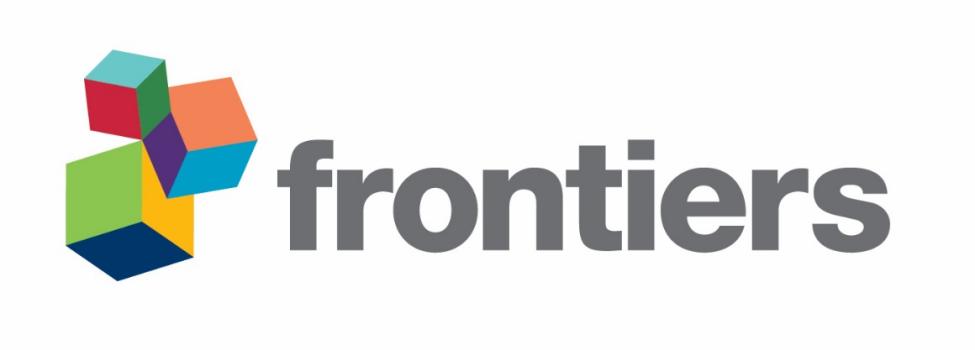
**

**Supplementary Figure 1.** The figure legends are required to have the same font as the main text, 12 point normal Times New Roman, single spaced. Please use a single paragraph for each legend and prepare the figures keeping in mind the PDF layout.
